# Metal Borohydrides beyond Groups I and II: A Review

**DOI:** 10.3390/ma14102561

**Published:** 2021-05-14

**Authors:** Karina Suárez-Alcántara, Juan Rogelio Tena García

**Affiliations:** Morelia Unit of Materials Institute Research, National Autonomous University of Mexico, Morelia, Michoacán 58190, Mexico; kse_91@hotmail.com

**Keywords:** borohydrides, transition metals, lanthanides

## Abstract

This review consists of a compilation of synthesis methods and several properties of borohydrides beyond Groups I and II, i.e., transition metals, main group, lanthanides, and actinides. The reported properties include crystal structure, decomposition temperature, ionic conductivity, photoluminescence, etc., when available. The compiled properties reflect the rich chemistry and possible borohydrides’ application in areas such as hydrogen storage, electronic devices that require an ionic conductor, catalysis, or photoluminescence. At the end of the review, two short but essential sections are included: a compilation of the decomposition temperature of all reported borohydrides versus the Pauling electronegativity of the cations, and a brief discussion of the possible reactions occurring during diborane emission, including some strategies to reduce this inconvenience, particularly for hydrogen storage purposes.

## 1. Introduction

**Figure 1:**
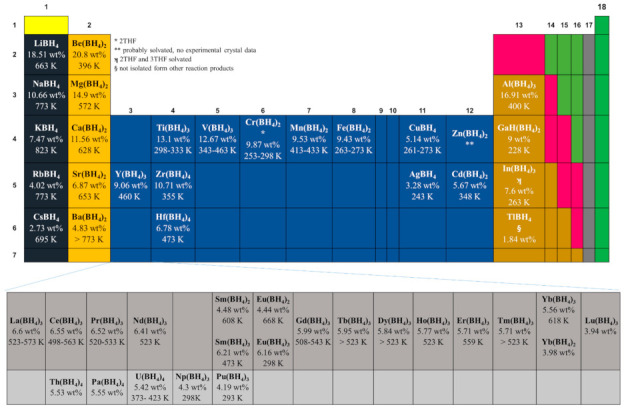
“Periodic table” of experimentally reported single homoleptic borohydrides showing hydrogen content in wt.% and decomposition temperature (or range). An editable version is in the supplementary file.

**Figure 2:**
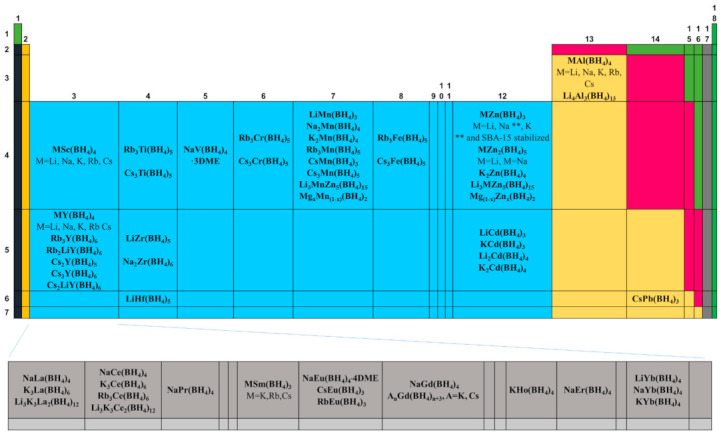
“Periodic table” of experimentally reported mixed-cation homoleptic borohydrides of transition metals, main group, lanthanides, and actinides. An editable version is in the supplementary file.

To date, several reviews on the alkali or alkali earth metal borohydrides for hydrogen storage have been published [1,2]. This great interest is due to the high hydrogen content of some of them, e.g., LiBH_4_. However, other metal borohydrides can be important for several applications, including, but not limited to, hydrogen storage. For example, the transition metal borohydrides present dehydrogenation temperatures which are usually lower than those of Group I and Group II borohydrides. In some cases, the hydrogen content is interesting (6–8 wt.%) due to the oxidation states (+2, +3) that translate into a high number of [BH_4_]^−1^ ions (ligands) [3] (See Figure 1). Recently, bimetallic and trimetallic materials with high hydrogen content have emerged (See Figure 2). Non- alkali or alkali earth metal borohydrides have many exciting characteristics that can help design new materials and applications beyond hydrogen storage. For example, lately, borohydrides have gained attention as high-temperature conductors in the solid-state or even when melted [4,5,6]. Borohydrides can also act as precursors to the corresponding metal boride [7,8,9,10] for the CVD-growing of thin films [11,12]. Rare earth metal borohydrides are emerging as new materials [13] for ion conductivity [5,14], catalysis [15,16], luminescence [17,18], or magnetic [19,20] applications.

In the late 1970s, a timely and comprehensive review of transition metals, lanthanide, and actinide borohydride complexes was reported [21]. In 2015, an update which specialized in crystal chemistry was published [22]. Since 2017, an important review of borohydrides and derivates (including heteroleptic materials of the type M_x_(BH_4_)_y_(NH_2_)_z_ and M_x_(BH_4_)_y_·nNH_3_) has been available for consultation [23]. In 2020, a highly informative paper on several hydrogen storage materials, including some borohydrides, was published [24]. In the last decade, research on the synthesis and characterization of bimetallic, trimetallic, anion-substituted (halide-substituted being more specific), and reactive composite mixtures of borohydrides has emerged, and it deserves a compilation. This work is a comprehensive review of the homoleptic borohydrides of transition metals, lanthanides, actinides, and main group block elements (Figure 1 and Figure 2, editable versions of these figures are in the supplementary file), focusing on the hydrogen storage area, yet covering (when available) data for other applications. When considered necessary, halide-substituted borohydrides are also mentioned.

In the last sections, an overview of the thermodynamics (stability) of borohydrides is included. A section called “the boron problem” is included to discuss the contradictory problem of desirable low-temperature dehydrogenation and the release of B_x_H_y_ compounds.

## 2. Synthesis

Details of the synthesis are presented for each borohydride; however, in this short section, a recount of the generals of borohydrides’ syntheses is presented. Synthesis methods can be divided roughly into two types: (i) “wet” synthesis methods in different organic solvents, and (ii) ball-milling synthesis. Wet synthesis has the advantages of better controlling the reaction conditions and products with higher purity, particularly with the new tendency of using mixtures of solvents or co-solvents during reactions and for product extraction. Some borohydrides have actually been produced only by this type of synthesis. Its drawbacks are the use of non-ecofriendly solvents and that some solvated materials are difficult to dry without them decomposing. For its part, ball-milling techniques produce impure borohydrides due to side reactions, by-products, and co-products (e.g., in the metathesis reactions). All these impurities may not always be inert during further reactions (i.e., during dehydrogenation reactions). The advantages of ball-milling are fast production, the possibility of scaling-up, and the chance to control temperature, pressure, and atmospheres during gas–solid reactions.

As additional types of synthesis method, we can include the “combined methods.” Combined methods generally start with the pre-milling of raw materials, followed by their reaction in solvents and solvent extraction or, recently, the use of S(CH_3_)_2_⋅BH_3_ as both a solvent and a reactant [13,25]. In all cases, precautions must be taken due to the materials’ sensitivity to oxygen and moisture; handling, synthesis, storage, and characterization must be performed under protective atmospheres (argon, nitrogen, etc.) of high purity.

The general synthesis routes of homoleptic borohydrides can be classified as follows [23,26,27]:

1. Direct synthesis from elements. Usually, this kind of synthesis requires high pressure and temperature. Reducing particle size can (but not always) accelerate reaction kinetics. An example is the synthesis of LiBH_4_ from Li, B, and H_2_ at 150 bar and a temperature of 975 K [26].
2. Syntheses using B_2_H_6_: For example, during wet synthesis, reagents such as Mg(CH_2_CH_3_)_2_ and B_2_H_6_ can react to produce the corresponding Mg-borohydride [28]. In solid–gas-type reactions, a metal hydride and B_2_H_6_ can react during ball milling. Notice that B_2_H_6_ is a toxic and unstable gas.
3. Reactive composites of metals or metal hydrides and metal borides. This type of synthesis has been widely demonstrated for Li, Na, and Ca-borohydrides. The metal borides serve as a B-source, enhancing the kinetics and reducing thermodynamic constraints. Importantly, this kind of synthesis has demonstrated some degree of reversibility, particularly if MgH_2_ and MgB_2_ are involved.

2MH + MgB_2_ + 4H_2_ → 2MBH_4_ + MgH_2_, for M = Li, Na (1)

CaH_2_ + MgB_2_ + 4H_2_ → Ca(BH_4_)_2_ + MgH_2_ (2)

2LiH + AlB_2_ + 3H_2_ → 2LiBH_4_ + Al (3)

4. Metathesis. This type of reaction is the most widely used synthesis method for borohydrides beyond Groups I and II, both by wet chemistry and ball-mill assisted synthesis. Usually, LiBH_4_ or (to a minor extent) NaBH_4_ are the preferred reagents due to their commercial availability. However, considering hydrogen storage applications, this kind of synthesis is useful only if the new material has better dehydrogenation kinetics, low dehydrogenation temperature, or better reversibility than the original LiBH_4_ or NaBH_4_. This statement is made because, normally, the hydrogen content of the new borohydride decreases compared to LiBH_4_; thus, other advantages over LiBH_4_ must be necessarily obtained. Additionally, unless proper separation of other metathesis products is accomplished, a significant decrease of the full mixture’s hydrogen content is unavoidable. The general reaction is:

*n*MBH_4_ + M′X_n_ → M′(BH_4_)_n_ + *n*MX (4)

A recent variation of this method involves the use of bulky anions and cations in solvents such as toluene, CH_2_Cl_2_, or CHCl_3_. For example [29]:

ZnCl_2_ + 2NaBH_4_ + [cat]BH_4_ → [cat][Zn(BH_4_)_3_] + 2NaCl (5)

M[an] + [cat][Zn(BH_4_)_3_] → M[Zn(BH_4_)_3_]↓ + [cat][an] (6)

where [cat] = [Ph_4_P]^+^ or [nBu_4_N]^+^; [an] = [Al{OC-(CF_3_)_3_}_4_]^−^ or [B{3,5-(CF_3_)_2_C_6_H_3_}_4_]^−^, M = Li, Na, K.

5. Metathesis-addition [23]. As described in the next sections, there is a relationship between metal electronegativity and dehydrogenation temperature [30]. Thus, in principle, the production of bimetallic and trimetallic borohydrides opens the way to tune the thermodynamics of dehydrogenation. For this purpose, the metathesis-addition reaction can be a useful tool.

*(x + ny)*MBH_4_ + *y*M′X_n_ → M_x_M′_y_(BH_4_)_x+ny_ + *ny*MX (7)

6. Synthesis using metal hydrides and S(CH_3_)_2_⋅BH_3_. This type of synthesis has evolved mainly for rare-earth (RE) metal borohydrides (RE(BH_4_)_x_). The synthesis reaction is preceded by the formation or conditioning of the corresponding metal hydride by mechanical milling. The reaction itself is carried out for a long time (even days of stirring with moderate heating) in toluene. Co-solvents such as dimethyl sulfide (DMS) can be used [13,17,31]. This reaction can be described as a nucleophilic addition mechanism [17]. Finally, extraction of the solvating S(CH_3_)_2_ molecules is performed by careful drying [13,31].

REH_3_ + 3S(CH_3_)_2_⋅BH_3_ → RE(BH_4_)_3_ S(CH_3_)_2_ + 2S(CH_3_)_2_ (8)

Special care must be taken during the drying process of borohydrides prepared in THF or ether. Some materials are simply delicate. In others, such as transition metals and RE-borohydrides, the solvent molecules cannot be removed without decomposing the borohydride. The use of low-coordination ability solvents [32] might be an option [24].

## 3. Bonding and Structure of Metal Borohydrides

**Figure 3:**
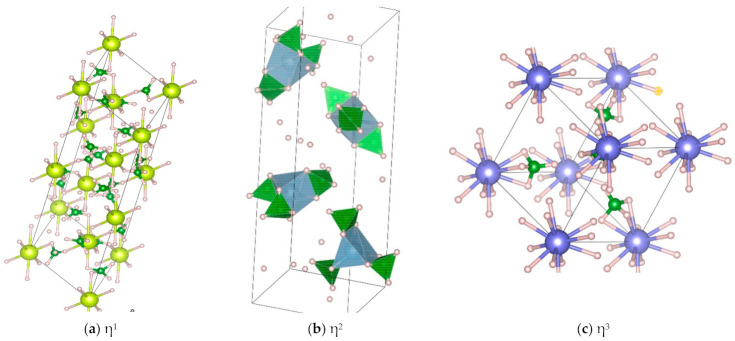
Examples of η^1^, η^2^, and η^3^ bonding between metals and [BH_4_]^−1^ ions: (**a**) crystal structure of r-Ce(BH_4_)_3_; (**b**) crystal structure of α-Al(BH_4_)_3_; (**c**) crystal structure of Zr(BH_4_)_3_. Yellow balls—Ce, grey balls—Al, purple balls—Zr, green balls—B, and light pink balls—H.

The bonding and structure details of each borohydride are given, when available, in each element subsection. In this section, general trends are commented on only in terms of tetrahydroborates complexes (only [BH_4_]^−1^ as ligands), monometallic (i.e., no bimetallic or trimetallic) compounds, and without bridging between two different metal centers. All these exceptions reflect the richness of the chemistry of metal borohydrides. A significant difference in comparison to the alkaline and alkaline earth borohydrides must be mentioned: for metals in Groups I and II, the bonding between [BH_4_]^−1^ and the metal ions is considered of an ionic nature [33]. This bonding type translates, as in any ionic compound, to highly stable materials, i.e., compounds with high dehydrogenation temperatures (Figure 1). In the transition metal borohydrides, the bonding spans from ionic to covalent coordination, which gives interesting properties of hapticity, H-mobility, H-exchange within the [BH_4_]^−1^ ligand or with other H-containing ligands, and reduced thermal stabilities in general (Figure 1 and Figure 2) [34,35,36]. The interaction of the [BH_4_]^−1^ ion with the transition metals occurs through bridging H atoms; thus, the hapticity can be η^1^, η^2^, or η^3^ (Figure 3). Due to the closeness of B atoms with the central metal in some η^3^ compounds, even an η^4^ interaction has been proposed [34]. The structures of the borohydride complexes around the metal center can have trigonal-planar, tetrahedral, square planar, trigonal-bipyramidal, or square-pyramidal octahedral geometry, especially if other ligands are present or if some H atoms of the [BH_4_]^−1^ are considered as a ligand. η^1^-, η^2^-, or η^3^-[BH_4_]^−1^ ligands are considered as two-, four-, or six-electron donor ligands in the borohydride metal–ligand interactions; therefore, some compounds such as M(η^3^-BH_4_)_4_ (M = Zr, Y or Hf) do not “follow” the 18-electron rule [37]. However, the 18-electron count is still obtained when symmetry restrictions account for orbital interactions [34]. The formation of compounds that exceed 18-electrons preferentially produces ionic complexes [37]. Another point to consider is the mean distance between the metal center and the B atoms: in general, for the three η^1^, η^2^, or η^3^ interaction modes, metals on the right side of the periodic table show larger distances than those on the left [34]. Moreover, first-row transition metals form shorter distances than those of the third row [34]. The integration of ligands different from [BH_4_]^−1^ can reduce hapticity, and change the symmetry and the ionic or covalent bond nature. Manipulation of all these factors may prove useful in the hydrogen storage area.

Vibrational spectroscopy (infrared and Raman) is very useful for characterizing the structure of all borohydrides [38], i.e., for characterization of the coordination modes of borohydrides. Marks et al. resumed the observed bands in mononuclear borohydrides, as shown in Table 1 [21]:

An excellent source (downloadable library) of IR-spectra of many borohydrides is the work of D´Anna et al. [39].

## 4. Transition Metal Borohydrides

### 4.1. Group 3: Scandium and Yttrium

In 2006, Nakamori et al. reported the formation of **Sc**(**BH_4_**)**_3_** in the solid-state by a metathesis reaction between LiAlH_4_ and ScCl_3_, activated by mechanical milling (5 h) [30]:

3LiBH_4_ + ScCl_3_ → Sc(BH_4_)_3_ + 3LiCl (9)

The calculated structural parameters of Sc(BH_4_)_3_ are collected in Table 2 [30]. In the report by Nakamori, the onset and peak dehydrogenation temperatures of the mixture 3LiBH_4_ + ScCl_3_ were 450 K and 550 K (as read approximately from a thermal desorption profile plot) [30]. The equivalent of Equation (9) in THF produced the mono-solvated material [40]. Further experiments indicated that the scandium borohydride reported in [30] was most probably **LiSc**(**BH_4_**)**_4_**, because no confirmation of the Sc(BH_4_)_3_ was reported. Data published in 2008 demonstrated that a change in the molar ratio of reaction Equation (9) produced LiSc(BH_4_)_4_ (Table 2) instead of Sc(BH_4_)_3_ [41,42]:

4LiBH_4_ + ScCl_3_ → LiSc(BH_4_)_4_ + 3LiCl (10)

**Figure 4:**
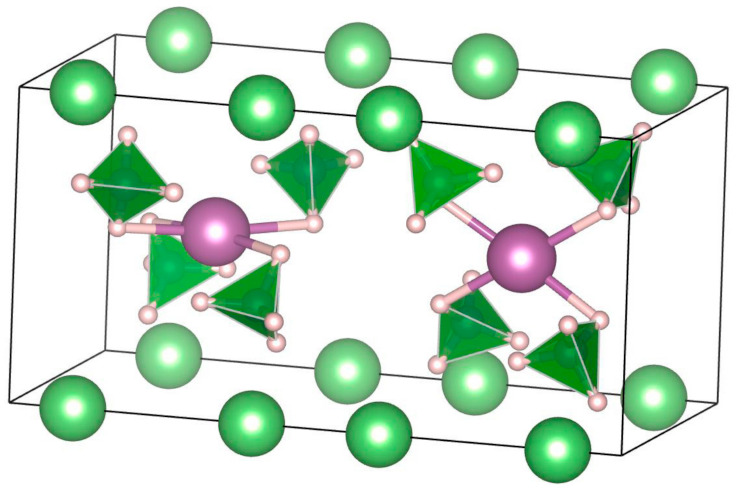
Crystal structure of LiSc(BH_4_)_4_, highlighting the [Sc(BH_4_)_4_]^−1^ complex ion: [BH_4_]^−1^—green tetrahedra, Sc—pink ball, and Li—green ball. Detailed crystallographic information is available in Table 2.

In LiSc(BH_4_)_4_, the scandium borohydride is, in fact, a complex ion: [Sc(BH_4_)_4_]^−1^ (See Figure 4). The formation of complex borohydride ions is a characteristic shared with transition metal borohydrides, frequently facilitated by changes in the precursors’ molar ratio.

Černý et al. found a significant mass loss (decomposition) of LiSc(BH_4_)_4_ in a range between 415 K and 535 K [43]. Employing DFT modeling, Kim et al. confirmed the stability of LiSc(BH_4_)_4_ at room temperature [44]. They proposed a complex multistep dehydrogenation reaction in which LiBH_4_, LiB_12_H_12,_ and ScH_2_ would act as intermediaries, while LiH, ScB_2_, and ScB_12_ (in addition to H_2_) would be the final products [44]. Kim et al. confirmed the formation of ScB_2_ upon heating to 673 K and between 0.1 and 0.3 MPa hydrogen pressure [44]. Re-hydrogenation at 673 K and 7 MPa H_2_ pressure increased the Li_2_B_12_H_12_ content while forming a small amount of LiBH_4_ [44]. No more recent reports were found describing the dehydrogenation/rehydrogenation reactions of LiSc(BH_4_)_4_.

**NaSc**(**BH_4_**)**_4_** and **KSc**(**BH_4_**)**_4_** also have been produced, and their crystallographic properties characterized (Table 2). For the production of NaSc(BH_4_)_4_, Černý et al. tried different stoichiometric ratios between NaBH_4_ and ScCl_3_. Independently of the ratio, the synthetic reaction promoted by ball-milling was described as [43]:

4NaBH_4_ + 2ScCl_3_ → NaSc(BH_4_)_4_ + Na_3_ScCl_6_ (11)

NaBH_4_ might be regenerated during the first dehydrogenation step of NaSc(BH_4_)_4_ (440–490 K) [43]. Na_3_ScCl_6_ participates in further dehydrogenation reactions. In the second step (495–540 K), NaBH_4_ (remanent from the synthesis and also formed during the first decomposition step) reacts with Na_3_ScCl_6_; a solid solution of Na(BH_4_)_1−x_Cl_x_ is proposed to be formed. NaCl and ScB_x_ are among the final dehydrogenation products [43].

Several theoretical studies [45,46,47,48] followed the experimental report of Černý et al. [43]. Kim proposed, based on first-principle calculations, that the decomposition reaction would be [47,48]:

NaSc(BH_4_)_4_ → ScB_2_ + 3/4 NaBH_4_+ 1/8 Na_2_B_10_H_10_ +47/8 H_2_ (12)

Alternative structures and dehydrogenation reactions of (12) have been reported; for example, H. D. Tran et al. reported that NaSc(BH_4_)_4_ with an orthorhombic *C222_1_* structure [45] would be more stable than the orthorhombic *Cmcm* structure reported by Černý et al. (Table 2) [43]. The formation enthalpy of orthorhombic *Cmcm* NaSc(BH_4_)_4_ was independently calculated as −72.69 kJ/mol H_2_ [46]. The theoretical calculations for NaSc(BH_4_)_4_ indicated an electrical insulator with a band gap of 5.055 eV [48].

**KSc**(**BH_4_**)**_4_** was reported in 2010; this material presents similarities with NaSc(BH_4_)_4_ but not with LiSc(BH_4_)_4_ [49]. The synthesis reaction promoted by ball-milling was:

4KBH_4_ + 2ScCl_3_ → KSc(BH_4_)_4_ + K_3_ScCl_6_ (13)

Several stoichiometric ratios between KBH_4_ and SCl_3_ were tested; the best ratio was 2:1 [49]. KSc(BH_4_)_4_ melts at ~405 K. The decomposition of KSc(BH_4_)_4_ at 460–500 K increases the amount of KBH_4_ (additional to that not reacted during synthesis) [49]. The second step at 510–590 K would involve the reaction of KBH_4_ with K_3_ScCl_6_, forming K(BH_4_)_1-x_Cl_x_. The crystal structure was determined and is reported in Table 2 [49]. No further experimental reports were found on KSc(BH_4_)_4_.

**RbSc**(**BH_4_**)**_4_** and **CsSc**(**BH_4_**)**_4_** were recently reported [50]. Both materials were produced by mechanical milling and solvent metathesis; thus, a direct comparison of the effects of the co-metathesis product was made. The synthesis reactions are [50]:

ScCl_3_ + 3MBH_4_ → 0.5MSc(BH_4_)_4_ + MBH_4_ + 0.5M_3_ScCl_6_ (14)

ScCl_3_ + 2MBH_4_ → 0.5MSc(BH_4_)_4_ + 0.5M_3_ScCl_6_ (15)

ScCl_3_ + MBH_4_ + 3LiBH_4_ → MSc(BH_4_)_4_ + 3LiCl (16)

where M = Rb, Cs. Pure borohydrides were extracted with dimethyl sulfide (DMS). RbSc(BH_4_)_4_ presents an orthorhombic *Pbcm* space group, while CsSc(BH_4_)_4_ is monoclinic *P2_1_/c* (Table 2) [50]. Starobrat et al. reported that, in RbSc(BH_4_)_4_, the Rb^+^ and [Sc(BH_4_)_4_]^−1^ ions create zig-zag lines of alternate ions along the c-axis [50]. Meanwhile, in CsSc(BH_4_)_4_, Cs^+^ and [Sc(BH_4_)_4_]^−1^ have three counterions as nearest neighbors [50]. Decomposition of pure and LiCl-mixed RbSc(BH_4_)_4_ is preceded by an endothermic event at 432 K, associated with a melting or phase transition. The main decomposition step was located at 510 K and was described as mainly endothermic but with an exothermic shoulder [50].

Decomposition of pure RbSc(BH_4_)_4_ was described as [50]:

RbSc(BH_4_)_4_ → RbBH_4_ + 6H_2_ + B + ScB_2_ (17)

However, a minor reaction associated with the diborane evolution was described as:

RbSc(BH_4_)_4_ → RbBH_4_ + 9/2H_2_ + 1/2 B_2_H_6_ + ScB_2_ (18)

In the sample containing LiCl, a third endothermic event at 559 K was reported. Finally, RbBH_4_ decomposed above 773 K [50].

CsSc(BH_4_)_4_ decomposition presents a melting or phase transition event at 480 K, a hydrogen release at 511 K, with minor B_2_H_6_ production (equations analogous to (17) and (18)), and probable formation of a ternary chloride at 532 K [50]:

CsSc(BH_4_)_4_ + 3LiCl → CsLi_2_Cl_3_ + 15/2 H_2_ + ScB_x_ + (4 − *x*)B + LiH (19)

CsBH_4_ decomposition into Cs, B, and H_2_ was observed at 695 K [50].

The first reports on yttrium borohydrides date to the 1960s. Back then, the compound was obtained as a THF abduct: Y(BH_4_)_3_(THF)_3_ [51,52] (and references therein). The usual reactants were YCl_3_ and LiBH_4_ in THF, but the reaction between Y(OCH_3_)_3_ with B_2_H_6_ in THF was also reported [51]. Later, in 2008, the metathesis synthesis of Y(BH_4_)_3_ was performed; it was assisted by ball-milling [53]:

YCl_3_ + 3LiBH_4_ → Y(BH_4_)_3_ + 3LiCl (20)

Alternatively, to avoid the formation of LiCl, **Y**(**BH_4_**)**_3_** can be produced by a gas–solid reaction promoted by mechanical milling [54,55]:

YH_3_ + 3/2 B_2_H_6_ → Y(BH_4_)_3_ (21)

Y(BH_4_)_3_ crystallizes in a cubic structure, *Pa−3 (205)* (low-temperature α-phase, Table 3); theoretical calculations give a formation enthalpy of −113 kJ/mol BH_4_ and predict Y(BH_4_)_3_ to be an insulator [53]. Y(BH_4_)_3_ (+3LiCl) has a solid (α to β) phase transition, the transition temperature was reported as 483 K [56] or 453 K [54,57]. In the high-temperature polymorph, the structure is cubic *Fm-3c (226)*; the [BH_4_]^−1^ ion is distorted from an ideal tetrahedron [58] and it can be considered an η^2^ type interaction with Y^+3^. However, Park et al. [55] and Remhof et al. [54] did not observe the α to β phase transition in Y(BH_4_)_3_ (from reaction (21)), i.e., without LiCl).

Dehydrogenation reactions have been described in several papers. In these published results stands out the multistep nature of the dehydrogenation reaction and some differences between the dehydrogenation pathway of pure Y(BH_4_)_3_ and Y(BH_4_)_3_ + 3LiCl (i.e., upon the synthesis method and purification). The decomposition of Y(BH_4_)_3_ + 3LiCl proposed by Jaroń et al. starts at 423–433 K with a small mass release (explained in that work as a probable melting phase transition), a second step at 533–678 K, and the last stage of dehydrogenation that starts above 698 K [56]. By its part, the dehydrogenation mechanism proposed by Ranvsbæk et al. first involved the Y(BH_4_)_3_ solid α to β phase transition, followed by the β-Y(BH_4_)_3_ decomposition, starting at 463 K and forming YH_3_, which then produced YH_2_ at 543 K [57]. The dehydrogenation mechanism also involved the evolution of an unidentified compound between 488 and 553 K and YB_4_ as a final decomposition product [57]. The occurrence of an unidentified peak during synchrotron radiation powder X-ray diffraction (SR-PXD) characterization was also observed by Frommen et al. between 473 and 520 K for a mixture of Y(BH_4_)_3_ + 3LiCl prepared by cryogenic ball milling [58]. Yan et al. proposed Y(B_3_H_8_)_3_ as the dehydrogenation intermediary’s possible identity [59].

Remhof et al. [54] and Park et al. [55] observed different decomposition pathways of Y(BH_4_)_3_ without LiCl. Y(BH_4_)_3_ (from Equation (21)) started decomposing at 460 K, with a maximum rate at 523 K. No indications of solid α to β phase transformation were found, but positive indications of melting right before decomposition were revealed by time-resolved in-situ XRD [54]. Park et al. proposed that LiCl slightly shifts the peak-temperature decomposition of Y(BH_4_)_3_ and promotes the formation of YB_4_ [55]. The decomposition products reported by Park et al. were YH_2_, YB_4_, Y, and Y_2_O_3_.

The decomposition pathway observed by Yan et al. in pure Y(BH_4_)_3_ (obtained by reaction (21) in ether, followed by precipitation and drying) comprises (a) phase transition, (b) melting, (c) decomposition of Y(BH_4_)_3_ into YH_3_, (d) decomposition of an intermediate phase, and (e) decomposition of YH_3_ into YH_2_ [60]. This pathway is more similar to the Y(BH_4_)_3_ + 3LiCl dehydrogenation studies reviewed above.

Lee et al., utilizing theoretical calculations, proposed a series of dehydrogenation reactions, where the products are a combination of YH_3_, YH_2_, YB_4_, YB_6_, B, Y, and H_2_ [61]. Given the experimental reaction products commented above, a possible first-step dehydrogenation reaction could be:

β-Y(BH_4_)_3_ → ¼ YH_3_ + ¾ YB_4_ + 45/8 H_2_ (22)

The calculated dehydrogenation temperature of (22) at P_H2_ = 0.1 MPa is ~195 K, ΔH^0^ = 22.5 kJ/mol H_2_ [61].

Another point to mention is the release of B-H compounds. The results are not unanimous; some reports indicate no release while others indicate the evolution of boron hydrides. On the other hand, the reported formation of YB_4_ [55] is good in terms of a possible rehydrogenation. Partial rehydrogenation was achieved at 35 MPa at 523 K and 573 K [60].

**LiY**(**BH_4_**)**_4_** and **NaY**(**BH_4_**)**_4_** were obtained by ball-milling of Li, Na, and Y-borohydrides, heating at 473 K and 453 K for the LiY(BH_4_)_4_ and NaY(BH_4_)_4_, respectively, and then quenching [62]. Another option is the use of weakly coordinating anions in weakly coordinating solvents such as CH_2_Cl_2_, i.e., equivalent to reactions (5) and (6) [63]. LiY(BH_4_)_4_ and NaY(BH_4_)_4_ are isostructural with the tetragonal *P-42c* LiSc(BH_4_)_4_, and orthorhombic *C222_1_* NaSc(BH_4_)_4_ (Table 3) [62]. Their values of ionic conductivities are 1.26 × 10^−6^ and 6.92 × 10^−7^ S cm^−1^ at room temperature, LiY(BH_4_)_4_ and NaY(BH_4_)_4_, respectively [62]. Dai et al., employing first-principles calculations, indicated that the formation of KY(BH_4_)_4_ is thermodynamically feasible; meanwhile, the formation of analogous materials with Li and Na is not possible [64]. LiY(BH_4_)_4_ and NaY(BH_4_)_4_ are, in fact, metastable materials that decompose during days or weeks at room temperature or upon heating to 402 K or 388 K, respectively [62].

**KY**(**BH_4_**)**_4_** was produced by the reaction between Y(BH_4_)_3_ (produced by reaction (20)) in a previous step, without elimination of LiCl) and KBH_4_ [65]:

Y(BH_4_)_3_ + KBH_4_ → KY(BH_4_)_4_ (23)

Reaction (23) was promoted by mechanical milling under a careful regime of alternating periods of 3 min milling/2–3 min resting. KY(BH_4_)_4_ is isostructural with NaY(BH_4_)_4_ (Table 3), and forms a complex anion [Y(BH_4_)_4_]^−1^ of a slightly distorted tetrahedral geometry [65]. Furthermore, [BH_4_]^−1^ ions have η^3^ interactions with Y [65]. The dehydrogenation of KY(BH_4_)_4_ is also a multistep process: (a) the first step is melting at 453 K; (b) from 463–483 K, an unidentified phase is detected; (c) the presence of KBH_4_ is confirmed and increases upon heating at higher temperatures (493 K); (d) a probable decomposition of YH_3_ into YH_2_ occurs at 543 K; and (e) two more TGA/DCS peaks are detected at 611 K and 632 K [65].

The mixture of NaBH_4_ and Y(BH_4_)_3_ (with LiCl) produced NaY(BH_4_)_2_Cl_2_ upon heating at 438 K [65]. One year later, in 2012, a detailed study of NaY(BH_4_)_2_Cl_2_ was published [66]. The synthesis reaction promoted by mechanical milling is [66]:

2NaBH_4_ + YCl_3_ → NaY(BH_4_)_2_Cl_2_ + NaCl (24)

However, side reactions produced Na_2_YCl_6_ and Na(BH_4_)_1-x_Cl. NaY(BH_4_)_2_Cl_2_ crystallizes in the monoclinic *P2/c* space group symmetry. DFT optimization of the geometry indicates that the [BH_4_]^−1^ ions interacted via η^3^ with Y and η^1^ with Na. NaY(BH_4_)_2_Cl_2_ decomposed at ~573 K without a significant release of borane gases [66].

Binary Y-borohydrides **RbY**(**BH_4_**)**_4_**, **CsY**(**BH_4_**)**_4_**, **Rb_3_Y**(**BH_4_**)**_6_**, and **Cs_3_Y**(**BH_4_**)**_6_**, and the ternary materials **Rb_2_LiY**(**BH_4_**)**_6_** and **Cs_2_LiY**(**BH_4_**)**_6_**, were reported recently (Table 3) [67,68,69]. RbY(BH_4_)_4_ and CsY(BH_4_)_4_ were produced by mechanical milling of Y(BH_4_)_3_ and the corresponding Rb or Cs-borohydride [67]. However, the Y(BH_4_)_3_ was not purified and contained LiCl. Thus, the ball-milled powders were a mixture of the bimetallic borohydrides, Cl-substituted borohydrides (in Cs materials), and LiCl. Diffraction analysis resulted in a monoclinic *P2_1_/c* RbY(BH_4_)_4_, but the Y-Rb sublattice can be symmetrized to orthorhombic *Pnma*. The CsY(BH_4_)_4_ structure was refined as a *I4_1_/a* space group [67]. Decompositions of RbY(BH_4_)_4_ and CsY(BH_4_)_4_ were multistep processes that involved the formation of Cl-substituted bimetallic borohydrides: Rb_2_Li[Y(BH_4_)_6-x_Cl_x_] and Cs_2_Li[Y(BH_4_)_6-x_Cl_x_], i.e., the LiCl was not inert. The quickest decomposition step of RbY(BH_4_)_4_/LiCl occurred at 544 K, and above 523 K for CsY(BH_4_)_4_/LiCl [67].

### 4.2. Group 4: Titanium, Zirconium, and Hafnium

Ti(BH_4_)_3_ is an unstable, volatile material that decomposes spontaneously at room temperature [70,71]. The first attempt to produce it involved the reaction between a bed of LiBH_4_ (in excess) and vapors of TiCl_4_, and it was performed by Hoekstra and Katz in 1949 [72]. Since then, it has been observed that Ti^+4^ reduced to Ti^+3^, and, thus, the impracticability of a tetra-borohydride of titanium has been apparent [72]:

2TiCl_4_ + 8LiBH_4_ → 2Ti(BH_4_)_3_ + 8LiCl + B_2_H_6_ + H_2_ (25)

The reaction in organic solvents (tetrahydrofuran or ether) between LiBH_4_ and TiCl_4_ ended in the formation of adducts such as Ti(BH_4_)_3_·2THF or Ti(BH_4_)_3_·2O(C_2_H_5_)_2_ [73]. In fact, several phosphines of titanium borohydrides (similarly produced to the latter compounds) are stable materials that can be used as a catalyst for polymerization and hydrogenation of olefins [21,74]. The only report on the synthesis of Ti(BH_4_)_3_ in the solid-state was published by Fang et al. in 2009 [75]. They proposed the in-situ formation of Ti(BH_4_)_3_ after ball-milling of 3LiBH_4_ and TiF_3_ and further heating up to 403 K. Fang et al. also recommend not using TiCl_3_, as the result is the decomposition of LiBH_4_ in the milling process [75]. The tested materials released over 5 wt% of hydrogen at about 343–363 K. In the same work, the identification of Ti(BH_4_)_3_ was achieved by in-situ infrared spectroscopy during heating. During these in-situ measurements, the emergence of IR bands around 1500–1600 and 2500 cm^−1^ at about 353 K, and further depleting at roughly 363 K, were critical to detect the formation of Ti(BH_4_)_3_. Further decomposition of Ti(BH_4_)_3_ led to B, TiH_2,_ and H_2_; the proposed complete reaction is: [75]

3LiBH_4_ + TiF_3_ → Ti(BH_4_)_3_ + 3LiF → 3B + TiH_2_ +5H_2_ + 3LiF (26)

Later in 2014, Callini et al. reported the formation of Ti(BH_4_)_3_ by milling or mixing (few details were reported) an excess of LiBH_4_ and TiCl_3_ [35]. In that work, the formation of Ti(BH_4_)_3_ occurred on the surface of the mixtures; then, the compound was expelled to the gas phase. The decomposition of Ti(BH_4_)_3_ started at room temperature, and essentially ended at 333 K. The decomposition was observed as follows [35]:

Ti(BH_4_)_3_ → B_2_H_6_ + TiH_2_ + B + 2H_2_ (27)

Interestingly, the stabilization of Ti(BH_4_)_3_ was achieved recently by its confinement in a Zr-based MOF (Zr_6_O_4_(DBC)_6_, DBC =1,4-benzenedicarboxylate)) [76]. In such a condition, Ti(BH_4_)_3_ is reported to decompose through an intermediary stage at 350–430 K without diborane release. Instead, pentaborane (B_5_H_9_) is a dehydrogenation product that recombines at higher temperatures to produce higher boranes [76]. The stabilization was attributed to the occupation of few (1–2) molecules of Ti(BH_4_)_3_ in the Zr-based MOF pores of comparable size. This stabilization reduces the interaction between Ti(BH_4_)_3_ molecules in the gas phase and increases the interaction of Ti(BH_4_)_3_ with the Zr-MOF due to strong host–guest connections [76].

No experimental results on the solid-state molecular structure of Ti(BH_4_)_3_ have been reported; instead, its molecular structure in the gas phase was reported in 1991 [71] and confirmed by Ab-initio studies in 1993 [77]. The Ti(BH_4_)_3_ molecule poses a *C_3h_* symmetry with a planar Ti-B_3_ skeleton, and the [BH_4_]^−1^ ions are η^3^ ligands [71] (Table 4). Dain et al. also suggested, based on vapor pressure measurements, the existence of a Ti(BH_4_)_3_ dimer having bridging and terminal borohydroborate groups [71]. However, no experimental confirmation has been reported.

In a recent review on several materials for hydrogen storage (including metal hydrides, alloys, high entropy systems, borohydrides, etc.), Rb_3_Ti(BH_4_)_5_ and Cs_3_Ti(BH_4_)_5_ are enlisted [24] (and references within). The original sources are two theses which are rather difficult to access. Both materials were prepared by mechanical milling (not many details are given in the review) [24]:

3MBH_4_ + M′Cl_n_ + *n*LiBH_4_ → M_3_M′(BH_4_)_3+n_ + *n*LiCl, (28)

where M = Rb, Cs and M´ = Ti. Rb_3_Ti(BH_4_)_5_ and Cs_3_Ti(BH_4_)_5_ crystalize in tetragonal *I4/mcm* space group. The cell dimensions are a = 9.214(3) Å, and c = 16.130(5) Å in Rb_3_Ti(BH_4_)_5_. For Cs_3_Ti(BH_4_)_5_, the cell dimensions are a = 9.644(8) Å, and c = 16.426(15) Å [24].

**Zr**(**BH_4_**)**_4_** was first reported in 1949 (reaction (29)) [72]. Later, Zr(BH_4_)_4_ was prepared by reactions (30), (31), and (32) in ether or THF [78]. However, the separation of the products proved difficult [79,80]. After that, the solid-state reaction (32) was implemented [81].

NaZrF_5_ + 2Al(BH_4_)_3_ → Zr(BH_4_)_4_ + 2AlF_2_BH_4_ + NaF (29)

ZrCl_4_ + 2Al(BH_4_)_3_ → Zr(BH_4_)_4_ + 2AlCl_2_BH_4_ (30)

3Zr(OC_4_H_9_)_4_ + 8B_2_H_6_ → 3Zr(BH_4_)_4_ + 4B(OC_4_H_9_)_3_ (31)

ZrCl_4_ + 4LiBH_4_ → Zr(BH_4_)_4_ + 4LiCl (32)

Recently, the preparation of Zr(BH_4_)_4_ by ball milling (Equation (32) and the homologous reaction with NaBH_4_ [79]) and recovering by means of sublimation at low temperature (243 K) was reported [82,83,84,85]. Zr(BH_4_)_4_ is a volatile compound at room temperature, and its gas-phase structure is monomeric η^3^, with a tetrahedral arrangement and a rotational barrier of the [BH_4_]^−1^ ions of 13.9 kJ/mol [79,81]. The sublimation heat is between 55.9 and 56.9 kJ/mol [86]. Zr(BH_4_)_4_ displays d-orbital covalency [87].

The crystal structure of Zr(BH_4_)_4_ was determined at 100 K as cubic space group *P-43m* (Table 5); in the solid-state, [BH_4_]^−1^ coordination to Zr is also η^3^ (Figure 3) [83]. Crystals of Zr(BH_4_)_4_ melt at 302–305 K [72,79]. Igoshkin et al., employing molecular dynamics calculations, located a phase transition (solid to liquid or amorphous) at 400 K [86]. According to Gennari et al., the decomposition overlaps with melting (355 K) to give ZrB_2_, B_2_H_6,_ and H_2_ as reaction products [79]. For their part, Nakamori et al. reported thermal decomposition at ~460 K [30]. To be commented on is that B_2_H_6_ formation was detected even during ball-milling synthesis [79].

Similar to other transition metal elements, Zr can form ions of the type [Zr(BH_4_)_5_]^−1^ [88]:

LiBH_4_ + Zr(BH_4_)_4_ → LiZr(BH_4_)_5_ (33)

However, **LiZr**(**BH_4_**)**_5_** is unstable and decomposes into the initial borohydrides at ~253 K.

In a DOE annual progress report, **Na_2_Zr**(**BH_4_**)**_6_** is briefly mentioned [89]: “unlike Zr(BH_4_)_4_, (Na_2_Zr(BH_4_)_6_) is non-volatile and undergoes rapid elimination of 2–3 wt% H_2_ at 40–110 °C with no detectable B_2_H_6_ contamination”. Other attempts to stabilize Zr(BH_4_)_4_ include: (a) formation of combined coordination compounds, with NH_3_ as a ligand [84,85]; (b) formation of coordination compounds with other ligands [90]; and (c) the formation of a composite with cross-linked poly(4-vinylpyridine), which is a non-hygroscopic material that is stable for months [91].

**Hf**(**BH_4_**)**_4_** and Zr(BH_4_)_4_ present similarities in chemistry and properties, such as crystal structure, bonding, and possible use as thin-film precursors. Hf(BH_4_)_4_ was also first produced in 1949 by a reaction analogous to reaction (29)) [72]. Later, the equivalent of reaction (32) in the solid-state was demonstrated [87,92]. Hf(BH_4_)_4_ is also a volatile compound at room temperature, and its gas-phase structure is monomeric η^3^, with tetrahedral arrangement and high vapor pressure (~2 kPa at RT) [11,72,81,92]. The crystal structure of Hf(BH_4_)_4_ was determined at 110 K: cubic space group *P-43m* in the solid-state (Table 6); [BH_4_]^−1^ coordination to Hf is η^3^, with d-orbital covalency [87,93]. As in the case of Zr, Hf can form a complex ion, [Hf(BH_4_)_5_]^−1^ [88]:

(34) $${\mathrm{LiBH}}_{4}+\mathrm{Hf}({\mathrm{BH}}_{4}{)}_{4}\;\overset{\mathrm{ether},\;195K\;}{\to }\;\mathrm{LiHf}({\mathrm{BH}}_{4}{)}_{5},$$

which is unstable at low temperature and decomposes (~253 K) to produce the precursor materials [88]. The decomposition of Hf(BH_4_)_4_ produces HfB_2_, B_2_H_6,_ and H_2_ [11]. A possible use of Hf(BH_4_)_4_, Zr(BH_4_)_4_, and Ti(BH_4_)_3_ is as precursors for metal borides thin films; the proved advantages are a low temperature of the CVD process and a high conductivity in the thin films [11,94,95].

### 4.3. Group 5, Vanadium, Niobium and Tantalum

Few reports on vanadium borohydrides were located during the preparation of this review. In 2011, Yang et al. reported the formation of **V**(**BH_4_**)**_3_** by employing mechanical milling of NaBH_4_ and VCl_3_ [96].

3NaBH_4_ + VCl_3_ → V(BH_4_)_3_ + 3NaCl (35)

It is worth mentioning that the temperature in Yang´s ball millings was carefully controlled at 258 K and 293 K by means of a cooling jacket [96]. In the same work, characterization by XRD after-milling did not give clear evidence of the formation of V(BH_4_)_3_; however, changes in the dehydrogenation pathway compared to pure NaBH_4_ served as an indirect proof [96]. The same low crystallinity of the milled products was observed by Korablov et al. in mixtures of LiBH_4_-VCl_2_ and NaBH_4_-VCl_2_ [97]. Dehydrogenation of V(BH_4_)_3_ by Yang et al. occurred in a three-step process, with main weight losses at 343 K, 416 K, and 518 K [96]. The dehydrogenation enthalpy was calculated as 25.4 kJ/mol V(BH_4_)_3_ [96]. In the report by Korablov et al., a new vanadium borohydride that decomposed at ~463 K was assumed [97].

A bi-cationic borohydride of Vanadium was reported as NaV(BH_4_)_4_·3DME (DME = 1,2-dimethoxyethane) [98]:

(36) $$5{\mathrm{NaBH}}_{4}+{\mathrm{VCl}}_{4}\;\overset{\mathrm{DME}}{\to }\;\mathrm{NaV}({\mathrm{BH}}_{4}{)}_{4}\cdot 3\mathrm{DME}+4\mathrm{NaCl}+½\;B_{2}H_{6}+½\;H_{2}$$

(37) $$4{\mathrm{NaBH}}_{4}+{\mathrm{VCl}}_{3}\;\overset{\mathrm{DME}}{\to }\;\mathrm{NaV}({\mathrm{BH}}_{4}{)}_{4}\cdot 3\mathrm{DME}+3\mathrm{NaCl}$$

NaV(BH_4_)_4_·3DME decomposes at 353–378 K to produce H_2_, B_2_H_6_, and DME [98]. Alternatively, Jensen et al. reported the reactions of VOCl_3_, VCl_4,_ or VCl_3_ with NaBH_4_ in an organic solvent that produced [Na(DME)][V(BH_4_)_4_] [99]. Treatment of that compound with PMe_3_, or the reaction of LiBH_4_ with VCl_3_(PMe_3_)_2_, produced V(BH_4_)_3_(PMe_2_)_3_ [99,100]. The analogous reaction between VCl_2_(dmpe)_2_ and NaBH_4_ produced V(BH_4_)_2_(dmpe)_2_ (dmpe = 1,2-Bis(dimethylphosphino)ethane) [100]. In the different reactions considered in all these reports, [V(BH_4_)_4_]^−1^ and V(BH_4_)_3_ seem to become stabilized with solvents and other ligands. Theoretical calculations indicate that NaV(BH_4_)_4_ and LiV(BH_4_)_4_ (i.e., [V(BH_4_)_4_]^−1^) would be stable [101,102].

In 1961, Nöth indicated that the reactions between alkali borohydrides (LiBH_4_, NaBH_4_) and halides of Hf, Th, V, Nb, Ta, Cr, Mo, W, and U, were not reported at that time [103]. Until now (2021), no homoleptic Nb-borohydrides have been reported. Compared to Nb-alanates, for which a relatively wide variety of materials have been reported [104] (and references within), the lack of homoleptic Nb-borohydrides is exceptional. Alanates of the same metal are in general less stable than the corresponding borohydrides. Concerning Ta, no reports on borohydrides of this metal were located during the preparation of this review.

### 4.4. Group 6: Chromium, Molybdenum and Tungsten

Some old reports on Cr(II) heteroleptic complexes with THF (2 solvent molecules) [105], TMDEA (Tetramethylethylenediamine), or Py (pyridine) [106] have been found. In particular, Cr(BH_4_)_2_·2THF was produced between 231 and 253 K, being unstable at room temperature [105]. Nakamori et al. reported the reaction between LiBH_4_ and chromium chloride; however, no clear indication of the formation of chromium borohydride was presented in the data from infrared spectroscopy and hydrogen release by thermal desorption [107]. A theoretical report indicates that a hypothetical Cr(BH_4_)_4_ is unstable, but [Cr(BH_4_)_4_]^−1^ might be stabilized by a bulky cation [102]. In fact, Rb_3_Cr(BH_4_)_5_ and Cs_3_Cr(BH_4_)_5_ were produced recently by mechanical milling (Equation (28) with M′ = Cr), and they crystalized in tetragonal *P4_2_/mbc* [24]. The cell size of Rb_3_Cr(BH_4_)_5_ was reported as a = 9.182(3) Å and c = 16.209(6) Å; while the cell size of Cs_3_Cr(BH_4_)_5_ corresponds to a = 9.578(4) Å and c = 16.544(12) Å [24].

To the best of our knowledge, no experimental reports of homoleptic Mo and W borohydrides have been published.

### 4.5. Group 7: Manganese, Technetium and Rhenium

Manganese borohydrides have generated a large interest in recent years (mainly from 2010 to 2015) due to their high hydrogen content (9.53 wt.%), a certain balance between stability and low dehydrogenation temperature, and their similarities with Mg(BH_4_)_2_ in chemistry and crystal structures. **Mn**(**BH_4_**)**_2_** can be produced by a metathesis reaction between 2MBH_4_ and MnCl_2_ (M = Li, Na or K) in organic solvents with further solvent extraction, or by solid-state metathesis promoted by ball milling, with or without further purification, using solvents such as S(CH_3_)_2_ [108,109]. These methods give different yields and purities [108,110]. Particularly, the presence of a second metathesis product (i.e., LiCl or NaCl) could be important; reports suggest the possibility of partial substitution of [BH_4_]^-^ by Cl^-^ ions [111,112]. Details of the synthesis technique are important: in the preparation using THF, the solvated material, Mn(BH_4_)_2_·(THF)_3_, was obtained [113]; attempts to remove the solvent led to decomposition. However, another researcher successfully used THF and NaBH_4_ plus MnCl_2_ as precursors [114]. Solvent-free Mn(BH_4_)_2_ can also be obtained by using anhydrous ether [111,114]. Using a mixture of toluene/S(CH_3_)_2_, followed by the extraction of the manganese borohydride with S(CH_3_)_2_, and, finally, proper drying, the synthesis of Mn(BH_4_)_2_ can be considered reproducible [109]. This last procedure seems to be the most reliable for obtaining pure Mn(BH_4_)_2_. In the ball-milling preparation, the ratio between milling time and pauses seems very important to achieve the desired product [115]. Additionally, this synthesis method can lead to the formation of nanometric materials (~10–20 nm) [116] (and references within). A formation enthalpy of −58.89 kJ/f.u. (f.u. = formula unit) and a half-metallic nature of Mn(BH_4_)_2_ were determined through first-principle calculations [117]. Additionally, a low thermodynamic barrier for the metathesis reaction between LiBH_4_ and MnCl_2_ was observed [118].

**Figure 5:**
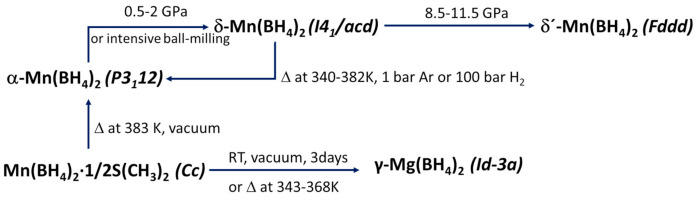
The interrelation of the phases of Mn(BH_4_)_2_, constructed with information from refs. [109,111,120].

Several polymorphs of Mn(BH_4_)_2_ have been reported (Figure 5, Table 7) [109,111,115]. The α-Mn(BH_4_)_2_ phase belongs to the trigonal *P3_1_12* space group symmetry, where Mn atoms are surrounded by four [BH_4_]^-^ ions in a distorted tetrahedral fashion [115], similar to *P3_1_12* Mg(BH_4_)_2_ [115,119]. Drying of Mn(BH_4_)_2_·1/2S(CH_3_)_2_ (after synthesis in toluene/2S(CH_3_)_2_) under vacuum at room temperature for three days produced γ-Mn(BH_4_)_2_ (cubic *Id-3a*), which is a nanoporous material similar to the zeolite-like γ-Mg(BH_4_)_2_ [109]. Some Mg(BH_4_)_2_ polymorphs are porous and can store small molecules in them [119]; thus, similar behavior can be expected from Mn(BH_4_)_2_. High-pressure polymorphs of Mn-borohydrides are: (i) δ-Mn(BH_4_)_2,_ which consists of two interpenetrating Mn(BH_4_)_2_ frameworks without voids, and (ii) δ′-Mn(BH_4_)_2_ [111]. The transition δ-Mn(BH_4_)_2_ to α-Mn(BH_4_)_2_ occurred upon heating at 340–382 K [111]. The possibility of a metastable Mn(BH_4_)_2_ phase with tetragonal *P-4n2* symmetry in the pressure range 0–1.5 GPa was also reported [111].

The dehydrogenation temperature of α-Mn(BH_4_)_2_ (without LiCl) was established between 413 and 433 K, depending on pressure conditions (vacuum and 1 bar Ar, respectively) [109]. Hydrogen release at higher temperatures is usually due to the presence of residual precursors, i.e., LiBH_4_ or NaBH_4_ [112,118,121]. Tomanov et al. reported a slightly wider range of dehydrogenation temperatures, between 403 and 473 K (without NaCl) [111]. The dehydrogenation reaction of α-Mn(BH_4_)_2_ can occur even at 100 bar hydrogen pressure [111]. Meanwhile, in a ball-milled sample of α-Mn(BH_4_)_2_ (+LiCl), manganese borohydride was found to melt at 450 K [115]. Varin et al. demonstrated good dehydrogenation kinetics under isothermal conditions (373–473 K) of mixtures of Mn(BH_4_)_2_ + LiCl (from ball-milled 2LiBH_4_ + MnCl_2_) [118]. The activation energy was between 70 and 59 kJ/mol, depending upon the milling conditions and the molar ratio of the precursors LiBH_4_ and MnCl_2_ [118]. A material stored at room temperature registered a release of 0.5 wt.% within a period of 80 days [118].

There is no consensus on the nature of the dehydrogenation products, particularly about the evolution of diborane and related compounds. Several dehydrogenation reactions have been proposed [112,118,121]:

Mn(BH_4_)_2_ → Mn + 2B + 4H_2_ (38)

Mn(BH_4_)_2_ → MnB_2_ + 4H_2_ (39)

3Mn(BH_4_)_2_ → 3Mn + 4B + B_2_H_6_ + 9H_2_ (40)

Regarding the diborane evolution, the terms used to describe the release range from practically undetectable to substantial amounts [109,116,122]. Liu et al. observed a peak of B_2_H_6_ evolution at 421 K [112], which is practically simultaneous to the hydrogen release. Recently, the addition of filamentary Ni, graphene, or LiNH_2_ (5 wt.%) reduced the B_2_H_6_ release [123].

Several authors agreed about the amorphous nature of the solid products of the dehydrogenation process, particularly in manganese borides. In this way, an interesting X-ray absorption spectroscopy study was performed by Guda et al. [124]. In this study, the Mn K-edge spectra of dehydrogenated samples are better described by the existence of Mn_2_B, MnB, and MnB_4_ than MnB_2_ and Mn_3_B_4_ compounds. The authors proposed the formation of the former borides and metallic Mn as the main dehydrogenation products [124]. However, in a recent report, Pankin et al. indicated that metallic Mn does not exceed 5% [121]. Unfortunately, the only attempt to re-hydrogenate at 473 K and 100 bar H_2_ (flowing at 100 mL/min in DSC) did not succeed [112].

Frequently, in addition to the formation of Mg(BH_4_)_2_, other compounds were obtained, such as bimetallic or solvated materials K_2_Mn(BH_4_)_4_ or M(Et_2_O)_2_Mn_2_(BH_4_)_5_ (M = Li, Na) [110,123]. Extraction of the solvent collapses the latter materials to Mn(BH_4_)_2_ and MBH_4_ (M = Li, Na) [110]. The solvated materials of (M(Et_2_O)_2_Mn_2_(BH_4_)_5_ and (M = Li, Na)) are insulators, with band gaps of 2.1 and 1.9 eV, respectively [110]. Dehydrogenation of M(Et_2_O)_2_Mn_2_(BH_4_)_5_ (M = Li, Na) involves the evolution of the solvent at 383 K, and the decomposition of Mn(BH_4_)_2_ at 433 K [110].

Bimetallic and trimetallic Mn-borohydrides can be of interest. **LiMn**(**BH_4_**)**_3_** was reported by Choudhury et al. in 2009. This material was produced by mechanical milling [125]:

3LiBH_4_ + MnCl_2_ → LiMn(BH_4_)_3_ + 2LiCl (41)

Being an amorphous material, its existence was demonstrated utilizing infrared spectroscopy [125,126,127]. LiMn(BH_4_)_3_ melts at about 367 K and undergoes dehydrogenation between 408 and 428 K [125]. As with other borohydrides, diborane release is a matter of controversy, from pure H_2_ [125] to 4 mol% [126]. Doping with transition metals (nano- Ni, Co, Fe, Ti Zn, Cu, Pd) [125] or Ti-compounds, such as TiF_3_, TiC, TiN, and TiO_2_, leads to a small reduction in the activation energy and the dehydrogenation temperature, particularly with Ni and TiF_3_ [125,126]. Activation energy is reduced from 130.64 kJ/mol to 111.55 kJ/mol in the Ni-doped material [125]. For the TiF_3_ doped LiMn(BH_4_)_3_ material, activation energy was reported as 114 kJ/mol. The reason for the reduction in activation energy by TiF_3_ is the partial formation of Ti(BH_4_)_3_, which decomposes at room temperature [126]. This supports the in-situ formation of Ti(BH_4_)_3_ observed by Fang et al. in 2009 [75]. Unfortunately, dehydrogenated LiMn(BH_4_)_3_ seems to be irreversible towards hydrogen uptake [125,126,127].

Severa et al. mentioned that **Na_2_Mn**(**BH_4_**)**_4_** may be formed during ball milling of NaBH_4_ with MnCl_2_ [114]. However, more research about the existence and properties of this material should be performed.

**K_2_Mn**(**BH_4_**)**_4_** was produced as a minor product in a metathesis type reaction in Et_2_O [110], but this material can also be obtained by ball milling of KBH_4_-MnCl_2_ or Mn(BH_4_)_2_ and KBH_4_ [108]:

4KBH_4_ + 2Mn(BH_4_)_2_ → 2K_2_Mn(BH_4_)_4_ (42)

K_2_Mn(BH_4_)_4_ has a monoclinic *P2_1_/n* symmetry (Table 7), and the [Mn(BH_4_)_4_]^−2^ environment was described as a penta-capped trigonal prism [108]. The decomposition of K_2_Mn(BH_4_)_4_ (420 K) produced KBH_4_ and KMn(BH_4_)_3_; the crystal structure of the latter material was not solved unambiguously but proposed as tetragonal *P4/mbm* [108]. DFT calculations on [Mn(BH_4_)_4_]^−2^ indicate bidentate [BH_4_]^−1^ interaction with Mn [114].

**CsMn**(**BH_4_**)**_3_** was briefly mentioned in a recent report; it crystallizes in the monoclinic *Cc* space group symmetry [69]. **Rb_3_Mn**(**BH_4_**)**_5_** and **Cs_3_Mn**(**BH_4_**)**_5_** were produced by mechanical milling (Equation (28) with M′ = Mn), and they crystalized in tetragonal *I4/mcm* [24]. The cell size is a = 9.2963 (19) Å, c = 16.101(3) Å for Rb_3_Mn(BH_4_)_5_, and a = 9.716(2) Å, c = 16.354 4) Å for Cs_3_Mn(BH_4_)_5_[24].

As with other interesting borohydrides, the formation of reactive mixtures or composites with Mn(BH_4_)_2_ is the subject of current research. Mixtures of Mn(BH_4_)_2_ + M(BH_4_)_x_, M = Li, Na, Mg, and Ca can be prepared by ball-milling from pure borohydrides. For M = Mg, a solid solution was also formed. Mn(BH_4_)_2_ and Mg(BH_4_)_2_ can form a solid solution of Mg_x_Mn_(1-x)_(BH_4_)_2_ in the range x = 0–0.8 from the mixing of MgCl_2_, MnCl_2,_ and LiBH_4_ [128]. This solid solution crystallized in the trigonal form of Mn(BH_4_)_2_, and it has the advantage of reducing the dehydrogenation temperature as compared with Mg(BH_4_)_2_ (425–450 K), while keeping a hydrogen content close to Mg(BH_4_)_2_ [128]. Mg_x_Mn_(1-x)_(BH_4_)_2_ can decompose with very slow kinetics at room temperature [128]. During decomposition, up to 7.5 mol.% of diborane can be released [128].

For the rest of the Mn(BH_4_)_2_ + M(BH_4_)_x_, M = Li, Na, and Ca composites, the decomposition of the components is not significantly affected by each other. Only small differences in the onset dehydrogenation temperature of Mn(BH_4_)_2_ were observed: 381 K in the mixture with LiBH_4_, 379 K with NaBH_4_, and 356 K with Ca(BH_4_)_2_ [120].

The composites of Mn(BH_4_)_2_ + MH_x_, M = Li, Na, and Ca, exhibit the same dehydrogenation mechanism; the decomposition of Mn(BH_4_)_2_ overlapped with the formation of M(BH_4_)_x_ [120]:

(43) $$\mathrm{Mn}({\mathrm{BH}}_{4}{)}_{2}+\frac{2}{3x}{\mathrm{MH}}_{x}\to \mathrm{Mn}+yB+3H_{2}+\frac{2}{3x}M({\mathrm{BH}}_{4}{)}_{x}$$

For the mixture of Mn(BH_4_)_2_/MgH_2_, no formation of Mg(BH_4_)_2_ was observed, and the dehydrogenation of the components appears to be independent of each other: 373–394 K for Mn(BH_4_)_2_ and 503–615 K for MgH_2_ [120].

**Li_3_MnZn_5_**(**BH_4_**)**_15_** (Table 7) can be produced by mechanical milling of LiBH_4_ and some salts [129]:

15LiBH_4_ + 5MnCl_2_ + 5ZnCl_2_ → Li_3_MnZn_5_(BH_4_)_15_ + 4LiCl + 4Li_2_MnCl_4_ (44)

13LiBH_4_ + Mn(BH_4_)_2_ + 5ZnCl_2_ → Li_3_MnZn_5_(BH_4_)_15_ + 10LiCl (45)

Reaction (45) can be preferred over the reaction (44) due to a competing reaction to form LiZn_2_(BH_4_)_5_ in the last one. Decomposition of Li_3_MnZn_5_(BH_4_)_15_ occurs at 385 K to produce LiZn(BH_4_)_5_, LiBH_4_, and Mn(BH_4_)_2_ [129].

No reports on technetium and rhenium borohydrides have been published.

### 4.6. Group 8: Iron, Ruthenium, and Osmium

**Fe**(**BH_4_**)**_2_** has a total hydrogen content of 9.42 wt.%. Schaeffer et al. prepared the mixture of LiBH_4_ and FeCl_3_ in ether at 228 K [130]. After the evaporation of the solvent, the filtrate was consistent with Fe(BH_4_)_2_, and the reduction of Fe(III) to Fe (II) was proposed [130]:

3LiBH_4_ + FeCl_3_ → Fe(BH_4_)_2_ + ½ H_2_ + ½ B_2_H_6_ + 3LiCl (46)

However, reaction (46) was not easily reproducible. The competing reaction of the reduction of Fe(III) to Fe (II), consuming 1 mol of LiBH_4_, was proposed to occur:

LiBH_4_ + FeCl_3_ → FeCl_2_ + 2H_2_ + B + LiCl (47)

Fe(BH_4_)_2_ was reported to decompose upon heating between 263 K and 273 K; possible decomposition reactions are [130]:

Fe(BH_4_)_2_ → Fe + H_2_ + B_2_H_6_ (48)

Fe(BH_4_)_2_ → Fe + 2B + 4H_2_ (49)

In turn, Varin et al. mixed 2LiBH_4_ + FeCl_2_ by means of milling in “soft conditions” (2–30 min at room temperature, under ultra-high purity H_2_ atmosphere and magneto ball-milling) [131,132]; infrared spectroscopy and X-ray diffraction results indicated the formation of a disordered Fe(BH_4_)_2_ that can decompose easily [131,132]. Recently, Rb_3_Fe(BH_4_)_5_ and Cs_3_Fe(BH_4_)_5_ were produced by mechanical milling (Equation (28), M’ = Fe), and they crystalized in tetragonal *I4/mcm* [24]. The cell parameters of Rb_3_Fe(BH_4_) are a = 9.171 (4) Å, and c = 15.833(6)Å, while the cell size of Cs_3_Fe(BH_4_)_5_ involves a = 9.619(11) Å, and c = 16.014 (19) Å [24].

To the best of our knowledge, no reports on ruthenium and osmium borohydrides have been published.

### 4.7. Group 9: Cobalt, Rhodium, and Iridium

In 1956, Stewart and Scheaffer indicated that the reaction of CoBr_2_ and LiBH_4_ in ether at liquid nitrogen temperature produced a greyish-white precipitate. This precipitate was presumed to be cobalt borohydride; this material turned black (decomposed) in a few minutes when warmed to room temperature, accompanied by the release of hydrogen and the formation of LiBr and CoB_2_ [133]. No characterization of the greyish-white precipitate was reported. Recently, a DFT screening of ternary alkali-transition metal borohydrides indicated that **KCo**(**BH_4_**)**_3_** and **NaCo**(**BH_4_**)**_3_** would be more stable than **Co**(**BH_4_**)**_2_**. Both binary Co-borohydrides were remarked as promising materials [101].

To the best of our knowledge, no reports on homoleptic rhodium and iridium borohydrides have been published.

### 4.8. Group 10: Nickel, Palladium, and Platinum

To the best of our knowledge, no reports on homoleptic borohydrides of nickel, palladium, and platinum have been published. However, heteroleptic (with chelates type-ligands) Ni-borohydrides are stable and can be used as catalysts [134].

### 4.9. Group 11: Copper, Silver, and Gold

Essentially, no experimental advances of the group 11 borohydrides have been reported since the 1960s. **CuBH_4_** was first reported by Wiberg; this material was produced by the metathesis reaction between CuCl and LiBH_4_ in a THF-ether (1:1) solution at 253 K [135]. However, CuBH_4_ decomposes in solution at 261–273 K, producing CuH, B, B_3_H_6_, and H_2_ [135,136]. An alternative is the use of CuCl_2_ in ether at 228K [136]:

CuCl_2_ + 2LiBH_4_ → CuBH_4_ + ½B_2_H_6_ + ½H_2_ + 2LiCl (50)

**AgBH_4_** can be produced by the following reaction in ether at 193 K [137]:

AgClO_4_ + LiBH_4_ → AgBH_4_ + LiClO_4_ (51)

AgBH_4_ decomposed at 243 K to produce Ag, H_2,_ and BH_3_; Wiberg proposed that Ag can catalyze the decomposition of BH_3_ to B and H_2_ [137].

**AuBH_4_** was proposed to be unstable by Wiberg, i.e., it will not form even at low temperature [138], but it was mentioned that Au(BH_4_)_3_ could be prepared by the reaction between AuCl_3_ and LiBH_4_ in ether at 153 K [139] (and ref. 39 within), but that it decomposed upon an increase in temperature. Unfortunately, no other references to Au(BH_4_)_3_ have been located. Theoretical calculations supported the statement of Wiberg; AuBH_4_ is not stable, while CuBH_4_ and AgBH_4_ would be stable with a rapid exchange of the tridentate and bidentate coordination of the [BH_4_]^−1^ ion [139].

### 4.10. Group 12: Zinc, Cadmium, and Mercury

**Zn**(**BH_4_**)**_2_** (8.48 wt.% hydrogen content) was first reported by Wiberg and Henle in 1952 [140]. Zn(BH_4_)_2_ was prepared by the metathesis reaction between ZnCl_2_ and LiBH_4_ in ether at room temperature [140]. Since then, it has become clear that Zn(BH_4_)_2_ decomposes at about 358 K to produce Zn, B, and H_2_ [140]. Other synthesis routes in the organic solvent THF were published in 1969 [141]:

2ZnBr_2_ + 2KBH_4_ → Zn(BH_4_)_2_ + K_2_ZnBr_4_ (52)

3Zn(OCH_3_)_2_ + 4(BH_3_)_2_ → 3Zn(BH_4_)_2_ + 2B(OCH_3_)_3_ (53)

Later, the preparation of Zn(BH_4_)_2_ by mechanical milling gained popularity. Beyond possible hydrogen storage applications, Zn(BH_4_)_2_ can be used in the reduction reaction of aldehydes and ketones [142] or as a source of B_2_H_6_ [143]. Choudhury et al. calculated that Zn(BH_4_)_2_ is an insulator material with a band gap of 3.529 eV [144].

**Figure 6:**
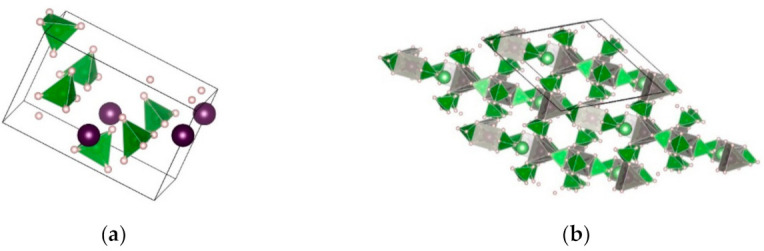
Crystal structure of (**a**) Zn(BH_4_)_2_ and (**b**) LiZn(BH_4_)_5_. The structures become complex with the formation of double metal borohydrides. Detailed crystallographic information can be found in Table 8.

The crystal structure of Zn(BH_4_)_2_ has only been calculated theoretically, with some degree of controversy (Table 8). Nakamori et al. determined the Zn(BH_4_)_2_ crystal structure as triclinic *P-1* (Figure 6) [30]. Choudhury et al., utilizing DFT calculations, determined that the orthorhombic *Pmc2_1_* (Mg(BH_4_)_2_ model) structure is the most stable at 0 K and finite temperatures [144]. For their part, Doan Huan et al. calculated that a tetragonal *I4_1_22* Zn(BH_4_)_2_ structure would be the most stable [145]. In another DFT calculation, Aidhy and Wolverton indicated that the tetragonal *I4m2* and orthorhombic *F222* structures of Zn(BH_4_)_2_ are near in energy, and have the lowest energies compared with other proposed structures [146]. The results of several stable structures point to the possible existence of polymorphism and low energies associated with transitions between them [145]. However, no experimental confirmation of the crystal structure of Zn(BH_4_)_2_ has been reported up to now. As in the case of Sc-borohydride, Zn-borohydride can be mistaken by a solvated product in wet synthesis, or as a bi-metallic borohydride in mechanically milled materials [147].

Zn(BH_4_)_2_ (with 2NaCl, from mechanical milling synthesis) melts at 358 K, followed by the dehydrogenation reaction, which is registered 358–413 at K [148]. However, a considerable evolution of diborane was recorded. Thus, the dehydrogenation reaction was described as [148,149]:

Zn(BH_4_)_2 (solid)_ → Zn(BH_4_)_2 (liquid)_ → Zn + B_2_H_6_ + H_2_ (54)

Due to the formation of B_2_H_6_ at low temperature, Zn(BH_4_)_2_ can be used as a diborane source for other reactions. For example, Friedrich et al. used diborane from Zn(BH_4_)_2_ into LiH to test the regeneration of LiBH_4_ in soft T and P conditions [143]. Srinivasan et al. did not limit the formation of B_2_H_6_ in their discussion of results, and proposed the possible formation of B_4_H_10_, B_5_H_9_, and B_6_H_12_ [150]. Srinivasan et al. demonstrated that 1.5 mol% nano-Ni particles reduced the formation of diborane [149,150]. Other additives such as TiCl_3_, TiF_3_, nano-Fe, Ti, nano-Ti [150], or carbon nanotubes [151] have demonstrated a small-to-moderate reduction in the decomposition temperature of Zn(BH_4_)_2_.

Interestingly, in Mg-Zn(BH_4_)_2_ and MgH_2_-Zn(BH_4_)_2_ (materials composed of two layers, and 1:1 mixtures, respectively) doped with Nb_2_O_5_, a reaction between Mg-Zn(BH_4_)_2_ and MgH_2_-Zn(BH_4_)_2_ occurred below 373K [152]. The authors proposed a reaction between MgH_2_ and B_2_H_6_ due to the appreciable reduction in diborane evolution and the change from the endothermic decomposition of Zn(BH_4_)_2_ to the exothermic decomposition of Mg-Zn(BH_4_)_2_ and MgH_2_-Zn(BH_4_)_2_ [152]. These reactions have the characteristics of a reactive hydride composite. Conversely, Zn(BH_4_)_2_ was tried as an additive for MgH_2_, with no significant improvement for MgH_2_ dehydrogenation kinetics [153,154].

The formation enthalpy of Zn(BH_4_)_2_ was calculated as −66.003 kJ/mol H_2_ at 300 K, while the dehydrogenation enthalpy (Zn, B, and H_2_ as the products) was calculated as 59.90 kJ/mol H_2_ at 0 K [144]. Such formation/dehydrogenation enthalpy is above the target of 20–50 kJ/mol H_2_. Thus, cation and anion substitution have been tried as strategies to tailor the formation or dehydrogenation thermodynamics. Bimetallic compounds have been reported in research on Zn-borohydrides as early as 1950–1969 [141]:

ZnCl_2_ + 3NaBH_4_ → NaZn(BH_4_)_3_ + 2NaCl (55)

**NaZn**(**BH_4_**)**_3_** has a monoclinic space group symmetry *P2_1_/c* (Table 8), where the Zn atoms present a distorted tetrahedral coordination, and metal atoms and [BH_4_]^−^ groups form a 3D framework [147]. It must be mentioned that different stoichiometric ratios can also produce **MZn**(**BH_4_**)**_3_** (**M = Li, Na**) (Table 8) [147,155]. A by-product, M_2_ZnCl_4_, plays an important role in dehydrogenation reaction [147].

**LiZn_2_**(**BH_4_**)**_5_** was produced by the reaction between LiBH_4_ (or Li^11^BD_4_) and ZnCl_2_ utilizing mechanical milling (either cryogenic or room temperature milling) [156]. The LiZn_2_(BH_4_)_5_ structure consists of two interpenetrating 3D frameworks of complex [Zn_2_(BH_4_)_5_]^−1^ ions and Li ions, with no bonds between the two different 3D frameworks, similar to some MOFs structures (Figure 6) [156].

**NaZn_2_**(**BH_4_**)**_5_** can also be produced by mechanical ball milling, with the generalized reaction of Li- and Na- Zn-borohydrides being [147,155,157]:

5MBH_4_ + 2ZnCl_2_ → MZn_2_(BH_4_)_5_ + 4MCl, (M = Li or Na) (56)

MZn_2_(BH_4_)_5_ (M = Li or Na) has a 3D framework structure, with two interpenetrating frameworks composed of an alkali-metal cation and a complex anion [Zn_2_(BH_4_)_5_]^-^, and built from two triangularly coordinated Zn atoms (Table 8) [147,155,157].

A first-principle study (DFT + PEGS (prototype electrostatic ground state)) indicated that LiZn(BH_4_)_3_ would have a triclinic *C1* space group symmetry [146]. LiZn(BH_4_)_3_ is calculated to be more stable (about −8 kJ/mol cation) as compared to the parent borohydrides of Li and Zn [146], but unstable towards decomposition to LiBH_4_ and LiZn_2_(BH_4_)_5_ (from DFT along with grand canonical linear programming method) [158]. In turn, LiZn_2_(BH_4_)_5_ is calculated to decompose in a rather complex pathway [158]:

LiZn_2_(BH_4_)_5_ → 2Zn + 1/5 LiBH_4_ + 2/5 Li_2_B_12_H_12_ + 36/5 H_2_ (57)

→ 2Zn + 1/6 LiH + 5/12 Li_2_B_12_H_12_ + 89/12 H_2_ (58)

→ 11/6 Zn + 1/6 LiZn + 5/12 Li_2_B_12_H_12_ + 15/2 H_2_ (59)

→ Zn + 5B + LiZn + 10H_2_ (60)

Ravnsbæk et al. agreed about the complex nature (coupled reactions) of dehydrogenation reactions and summarized them as [147]:

2MZn_2_(BH_4_)_3_ + M_2_ZnCl_4_ → 5Zn + 4MCl + 3B_2_H_6_ + 3H_2_ M = Li (at 400 K), Na (at 368 K) (61)

The same type of DFT + PEGS calculations on the analogous Na-Zn borohydrides indicated that NaZn(BH_4_)_3_ is unstable towards the decomposition of Na and Zn-borohydrides, while NaZn_2_(BH_4_)_5_ would be more stable than the parent borohydrides [146]. Xia et al. suppressed the production of diborane by infiltrating NaZn(BH_4_)_3_ in SBA-15 (prepared by wet-ball milling, dissolution in THF, infiltration, and drying) [159]. Additionally, a slight reduction in the dehydrogenation peak temperature was observed in the infiltrated material compared to the pure material (385 K versus 393 K, respectively) [159]. Under those circumstances, the decomposition of NaZn(BH_4_)_3_ produced NaBH_4_, Zn, B, and H_2_ [159].

**KZn**(**BH_4_**)**_3_** was prepared by the ball-milled assisted reaction between ZnCl_2_ and KBH_4_ [160]:

ZnCl_2_ + 3KBH_4_ → KZn(BH_4_)_3_ + 2KCl (62)

Then, a side-reaction might also occur:

ZnCl_2_ + 2KCl → K_2_ZnCl_4_ (63)

In Equation (63), a partial substitution of Cl^-^ by [BH_4_]^−1^ (K_2_ZnCl_4-x_(BH_4_)_x_) was registered and linked to the dehydrogenation reaction [160].

KZn(BH_4_)_3_ presents a trigonal space group symmetry *R3* (Table 8); the Zn atom is coordinated with three [BH_4_]^−1^ groups in a triangular planar configuration, suggesting the existence of an anionic complex [Zn(BH_4_)_3_]^−1^ located inside a rhombohedron of K atoms [160].

KZn(BH_4_)_3_ decomposes at ~385 K to produce a gas mixture of H_2_ and diborane, and another double cation borohydride [160]:

2KZn(BH_4_)_3_ → K_2_Zn(BH_4_)_4_ + Zn + B_2_H_6_ + H_2_ (64)

Further heating leads to multistep dehydrogenation reactions, involving an exchange of Cl^-^ and [BH_4_]^−1^ ions. For K_2_ZnCl_4-x_(BH_4_)_x_, an increase in [BH_4_]^−1^ content was registered, first at ~385 K but, upon further heating, [BH_4_]^−1^ content decreased [160].

In general, in the double cation M-Zn- borohydrides (MZn(BH_4_)_3_ and M_2_Zn(BH_4_)_4_, M = Li, Na and K), their synthesis by ball-milling, and the dehydrogenation reactions present many similarities.

**Mg**_(**1−x**)_**Zn_x_**(**BH_4_**)**_2_** (solid solution) was prepared by the mechanical milling of Mg(BH_4_)_2_ and ZnCl_2_ at room and liquid-N_2_ temperatures [161,162]. Decomposition was reported to start at 373–398 K with the release of hydrogen and diborane [161,162]. Careful control of the milling conditions and stoichiometry are important for the successful formation of the solid solution Mg_(1-x)_Zn_x_(BH_4_)_2_, dehydrogenation temperature, and products [161,162]. Cryogenic ball-milling offered a slight advantage in the preferential release of hydrogen over diborane [162]. A periodic DFT study of Mg_(1-x)_Zn_x_(BH_4_)_2_ (x = 0.2–0.3) indicates an energetically favorable mixing of Zn and Mg borohydrides and a decomposition enthalpy of about 30 kJ/mol H_2_ [163]. The DFT studies were performed by systematically replacing Mg atoms with Zn atoms in the α-Mg(BH_4_)_2_ structure (both metals present +2 oxidation state and similar ionic size, *r*_Mg_ = 0.065 nm and *r*_Zn_ = 0.074 nm [146]). Mg_0_._8_Zn_0_._2_(BH_4_)_2_ was claimed to be a promising composition [163].

Trimetallic borohydrides **Li_3_MZn_5_**(**BH_4_**)**_15_, M = Mg, and Mn**, were produced by mechanical milling [129], reactions (44) and (45). In both cases, the formation of LiZn_2_(BH_4_)_5_ as a by-product was observed. Li_3_MZn_5_(BH_4_)_15_, M = Mg and Mn, also crystalizes in a hexagonal *P6_3_/mcm* structure that contains channels built up from face-sharing (BH_4_)_6_ octahedra [129]. The Li_3_MZn_5_(BH_4_)_15_ materials, M = Mg and Mn, are interesting as solid-state electrolytes, and their decomposition (at 368–393 K for Mg, and 385 K for Mn) produces the related borohydrides LiBH_4_, Mg(BH_4_)_2_, Mn(BH_4_)_2_ and LiZn_2_(BH_4_)_5_. These latter borohydrides decompose at higher temperatures [129].

Chlorine-substituted Zn-borohydrides, such as LiZn(BH_4_)_2_Cl and Zn(BH_4_)Cl, were observed as early as 1952, with the latter compound decomposing at 393 K to produce ZnCl_2_, metallic Zn, B, and H_2_ [140]. KZn(BH_4_)Cl_2_ was reported in 2011, and it decomposes at about 383 K to produce K_2_ZnCl_4_, metallic Zn, boranes ((BH_3_)_n_), and H_2_ [164].

**Cd**(**BH_4_**)**_2_** was first reported in 1952 by Wiberg et al. [165]. At the time, cadmium borohydride was produced in ether:

CdCl_2_ + 2LiBH_4_ → Cd(BH_4_)_2_ + 2LiCl (65)

In ether, Cd(BH_4_)_2_ can also be obtained as [166]:

3Cd(OCH_3_)_2_ + 4B_2_H_6_ → 3Cd(BH_4_)_2_ + 2B(OCH_3_)_3_ (66)

Later, in 2013, the reaction (65) without solvents was reported, i.e., ball milling synthesis [167,168]. Two polymorphs were identified: α-Cd(BH_4_)_2_ and β-Cd(BH_4_)_2_ [167,168]. The temperature of the polymorph transition was located at ~328 K [167]. The formation of the polymorphs demonstrated a dependency on the milling temperature: the material prepared by cooling the milling vial (with liquid nitrogen) was mainly α-Cd(BH_4_)_2_, while room temperature milling was associated with the formation of β-Cd(BH_4_)_2_ [168]. However, one side reaction was observed in the solid-state preparation reaction that reduced the quantity of Cd(BH_4_)_2_ formation [168]:

LiBH_4_ + CdCl_2_ → 0.5Cd(BH_4_)_2_ + 0.5Li_2_CdCl_4_ (67)

The decomposition of Cd(BH_4_)_2_ was observed at room temperature [165], and even at lower temperatures [169]. Thus, cadmium borohydride is an unstable material. In a more detailed study, Cd(BH_4_)_2_ decomposed at 348 K with fast kinetics and produced B_2_H_6_ and H_2_ in a mole ratio of 1:1 [168]:

Cd(BH_4_)_2_ → Cd + B_2_H_6_ +H_2_ (68)

The formation of cation- and anion-substituted Cd(BH_4_)_2_ has been observed since the first reports [165] in organic solvents, such as ether or DMF (dimetylformamide) [170]:

CdCl_2_ + 2LiBH_4_ → LiCdCl(BH_4_)_2_ + LiCl (69)

CdCl_2_ + 3LiBH_4_ → LiCd(BH_4_)_3_ + 2LiCl (70)

Reactions (65) and (69) in ether, and particularly reaction (70), can also occur in the systems MBH_4_/CdX_2_, M = Li, Na and X = Cl, Br, I [166,170]. The complex ions [CdCl(BH_4_)_2_]^−1^ and [Cd(BH_4_)_3_]^−1^ were observed during the solvent synthesis, and the formation of adducts was demonstrated [169,170,171]. The addition of an equivalent of LiBH_4_ to LiCd(BH_4_)_3_ in diglyme can produce a precipitate soluble in THF [171]:

LiCd(BH_4_)_3_ + LiBH_4_ ↔ Li_2_Cd(BH_4_)_4_ (71)

The **Li_2_Cd**(**BH_4_**)**_4_** precipitated as an adduct with four molecules of diglyme [171]. The ball milling of MBH_4_/CdCl_2_, M = Li, Na, K, in several molar ratios (1:1 to 4:1) only succeeded in producing **KCd**(**BH_4_**)**_3_** and **K_2_Cd**(**BH_4_**)**_4_** [167]. The last product, K_2_Cd(BH_4_)_4_, is composed of tetrahedral [Cd(BH_4_)_4_]^−2^ ions and eight-coordinated K^+^ cations. KCd(BH_4_)_3_ has a structure that can be considered a framework intermediate between those of α-Cd(BH_4_)_2_ and β-Cd(BH_4_)_2_ [167]. Decomposition of the K-Cd borohydrides was proposed to be sequential [167]:

2KCd(BH_4_)_3_ → K_2_Cd(BH_4_)_4_ + Cd + B_2_H_6_ + H_2_, ~353 K (72)

K_2_Cd(BH_4_)_4_ → 2KBH_4_ + Cd + B_2_H_6_ + H_2_, ~358 K (73)

For the last element in group 12, i.e., Hg, Nöth mentioned that the reaction of LiBH_4_ with HgCl_2_ at 195 K produced a reduction in Hg without the formation of mercury borohydride [166].

## 5. Main-Group Borohydrides

### 5.1. Group 13: Aluminum, Gallium, Indium, and Thallium

**Al**(**BH_4_**)**_3_** is an “old” borohydride which has been known since 1939 [172,173]. Its standard formation enthalpy is 301.6 kJ/mol liquid Al(BH_4_)_3_ [174]. It is a very hazardous and volatile liquid at room temperature (melting point ~208 K), with an average heat of vaporization of 29.9 kJ/mol [173] that detonates on contact with air containing traces of moisture [175]. Al(BH_4_)_3_ can be produced by reactions (74) and (75) [172,173,175], and recovered by proper condensing (cooling) of the volatile product:

Al_2_(CH_3_)_6_ + 4B_2_H_6_ → 2B(CH_3_)_3_ + 2Al(BH_4_)_3_, 178–353 K (74)

AlX_3_ + 3MBH_4_ → Al(BH_4_)_3_↑ + 3MX, X = Cl or Br, M = Li, Na or K (75)

Al(BH_4_)_3_ was used to prepare other borohydrides (Th, U, Hf, Ti, etc.) in the 1940s [72]. Many years later, the low temperature α- and β-Al(BH_4_)_3_ crystalline structures were discovered (Table 9) [176]: α-Al(BH_4_)_3_ presents a monoclinic space group *C2/c*, while β-Al(BH_4_)_3_ presents an orthorhombic space group of *Pna2_1_*. Both polymorphs are composed of discrete molecular Al(BH_4_)_3_ units. The α- to β-Al(BH_4_)_3_ phase change occurs between 180 and 195 K [176]. In the gas phase, Al(BH_4_)_3_ presents a *D_3h_* symmetry with a planar Al-B_3_ skeleton and η^2^-[BH_4_]^−1^ ligands [176,177,178,179,180]. The heats of formation were calculated in 2006 by first principles, as −132 kJ/mol and −131 kJ/mol (without zero-point correction) for α- and β-Al(BH_4_)_3_, respectively [181]. The calculations also demonstrated the non-metallic nature of α-Al(BH_4_)_3_, with a calculated band gap of 6.0 eV [181]. Decomposition of Al(BH_4_)_3_ was predicted to be a spontaneous process above approximately 400 K, occurring with the release of B_2_H_6_ [70], but the experimentally observed decomposition occurred at room temperature [172,173]. In 2016, Harrison et al. proposed reducing diborane production, at least theoretically, by including a second cation, as in Al_1-x_Sc_x_(BH_4_)_3_ [182].

Thermal stabilization was achieved by the formation of double cation borohydrides of Al with group 1 alkali metals, i.e., the formation of [Al(BH_4_)_4_]^−1^ ions [183,184]. MAl(BH_4_)_4_, M = Li, Na, K, Rb, and Cs, can be produced by the following reaction [184]:

Al(BH_4_)_3_ + MBH_4_ → MAl(BH_4_)_4_ (76)

Reaction (76) was obtained by transferring the liquid Al(BH_4_)_3_ into the vessel that contains solid MBH_4_, and further purification was achieved by evaporation of the newly formed aluminum borohydride [185]. **Li_4_Al_3_**(**BH_4_**)**_13_** was always produced as a side product of reaction (76) when using LiBH_4_ [184]. Li_4_Al_3_(BH_4_)_13_ can be considered a decomposition product of **LiAl**(**BH_4_**)**_4_** because, upon the heating of LiAl(BH_4_)_4_ at 333 K, the following reaction occurs [184]:

4LiAl(BH_4_)_4_ → Li_4_Al_3_(BH_4_)_13_ + Al(BH_4_)_3_ (77)

**Li_4_Al_3_**(**BH_4_**)**_13_** can also be produced by the following reaction (under ball-milling with careful temperature and pressure monitoring) [186]:

3AlCl_3_ + 13LiBH_4_ → Li_4_Al_3_(BH_4_)_13_ + 9LiCl (78)

The crystal structures of LiAl(BH_4_)_4_ and Li_4_Al_3_(BH_4_)_13_ are monoclinic *P2_1_/c* and cubic *P-43n*, respectively (Table 9) [184]. The crystal structure of Li_4_Al_3_(BH_4_)_13_ is composed of the complex cation [(BH_4_)Li_4_]^+3^ and the [Al(BH_4_)_4_]^−1^ anion [186]. The decomposition of Li_4_Al_3_(BH_4_)_13_ occurred at 363 K, producing more Al(BH_4_)_3_ and LiBH_4_ [184,186,187].

**NaAl**(**BH_4_**)**_4_** was briefly mentioned by Dovgaliuk et al.; this material crystallized in the monoclinic space group *C2/c* and decomposed at 363 K to produce the parent Na and Al borohydrides [184]. **KAl**(**BH_4_**)**_4_** can be produced by direct contact of K and Al borohydrides at room temperature [185,188]:

KBH_4 (s)_ + Al(BH_4_)_3 (l)_ → KAl(BH_4_)_4 (s)_ (79)

Reaction (79) was reported to occur slowly for several days under high precautions due to the reactivity of Al(BH_4_)_3_ [188]. KAl(BH_4_)_4_ crystallized in the orthorhombic *Fddd* space group, where the [Al(BH_4_)_4_]^−1^ anion forms distorted tetrahedra [185].

Thermal decomposition of KAl(BH_4_)_4_ begins at 427 K, with a separation into the original borohydrides and an enthalpy of 36 kJ/mol [188]. Then, the decomposition of Al(BH_4_)_3_ was proposed to occur as [188]:

Al(BH_4_)_3 (g)_ → AlH(BH_4_)_2 (s)_ + 0.5B_2_H_6 (g)_ (80)

AlH(BH_4_)_2 (s)_ → AlH_2_(BH_4_)_(s)_ + 0.5B_2_H_6 (g)_ (81)

AlH_2_(BH_4_) _(s)_ → Al_(s)_ + 0.5B_2_H_6 (g)_ + 1.5H_2_ (82)

0.5B_2_H_6 (g)_ → (BH)*_n_*_(s)_ + H_2_ (83)

For their part, Dovgaliuk et al. proposed a one-step dehydrogenation of KAl(BH_4_)_4_ at 433 K (maximum DSC peak) [185]:

2KAl(BH_4_)_4_ → KBH_4_ + KAl_2_B_3_ + 2B_2_H_6_ + 8H_2_ (84)

Reaction (84) was proposed based on the combined results of several characterization techniques [185]. The decomposition product KAl_2_B_3_ is an amorphous material [185]. Decomposition of KBH_4_ must occur at higher temperatures. No re-hydrogenation was achieved up to 10 MPa hydrogen pressure and 593 K [185].

**RbAl**(**BH_4_**)**_4_***(*orthorhombic *Fddd)* and **CsAl**(**BH_4_**)**_4_** (tetragonal *I4_1_/amd*) were also briefly mentioned by Dovgaliuk et al.; these materials decomposed at 433 K and 423 K, respectively [184].

Homoleptic gallium borohydride does not exist. However, the related compounds, **GaH**(**BH_4_**)**_2_** and **GaH_2_**(**BH_4_**), were reported in [189,190]. GaH(BH_4_)_2_ can be produced in the absence of solvent at 228 K with an excess of LiBH_4_ [189,191,192]:

GaCl_3_ + 3LiBH_4_ → GaH(BH_4_)_2_ + 3LiCl + ½ B_2_H_6_, 228 K (85)

or

HGaCl_2_ + 2LiBH_4_ → GaH(BH_4_)_2_ + 2LiCl, 228 K (86)

These reactions are globally similar to the GaH_2_(BH_4_) synthesis [190]:

½ (H_2_GaC1)_2_ + LiBH_4_ → GaH_2_(BH_4_) + LiCl, 250 K, (87)

GaH(BH_4_)_2_ is volatile (melting point at approximately 203 K and vapor pressure of 10 mmHg at 228 K [189]), unstable, and decomposes above 228 K [189]. GaH_2_(BH_4_) is also unstable and decomposes upon heating [190]. Decomposition products of both compounds are Ga, H_2,_ and B_2_H_6_ [189,190].

**In**(**BH_4_**)**_3_** was reported in 1957, and this borohydride was obtained by the reaction in ether at 228 K [193]:

InR_3_ + (3 + 3/*n*)BH_3_ → In(BH_4_)_3_ + (3/*n*)BH_3−n_R_n_, R = methyl (88)

This reaction is slow (2 days). Another option (less efficient) is the following reaction in ether at 248 K [193]:

InCl_3_ + 3LiBH_4_ → In(BH_4_)_3_ + 3LiCl (89)

In(BH_4_)_3_ crystalized as In(BH_4_)_3_·3THF at 233 K and as In(BH_4_)_3_·2THF at 243 K, and decomposed at 263 K to produce metallic indium, hydrogen, and borane [193].

**TlBH_4_** was reported in 1957 [194]; no additional reports have been located. TlBH_4_ was synthesized by the reaction between thallium ethylate and lithium borohydride in ether at room temperature [194]:

TlOR + LiBH_4_ → TlBH_4_ + LiOR (90)

Purification of the TlBH_4_ was not possible, and decomposition of the whole mixture, TlBH_4_ + LiOR, was registered at 423 - 443 K [194]. The homoleptic borohydride of Tl(III) cannot be prepared, even at a low temperature; instead, TlCl(BH_4_)_2_ was produced at 163 K [195]:

TlCl_3_ + 2LiBH_4_ → TlCl(BH_4_)_2_ + 2LiCl (91)

TlCl(BH_4_)_2_ decomposed at 178 K producing hydrogen, borane, and TlCl [195].

### 5.2. Group 14: Lead

**CsPb**(**BH_4_**)**_3_** and **CsPb**(**BH_4_**)**_2_Cl** were briefly described in a recent report [69]. CsPb(BH_4_)_3_ is a semiconducting borohydride with an experimental band gap of ~1.5 eV at RT. CsPb(BH_4_)_3_ releases 4.3 wt%. hydrogen at 360 K [69].

## 6. Rare Earth Metal Borohydrides

In 1959, a report described the reactions between RE(OCH_3_)_3_, RE = Y, Sm, Eu, Gd, Td, Dy, Ho, Er, Tm, Yb, and Lu, with diborane in THF [51]. The products were solvated borohydrides, RE(BH_4_)_3_. As stated in the introductory section, the synthesis of these borohydrides currently follows a new approach in the sense of combining mechanical milling with solvent extraction, solvent and reactant function of precursors, or the use of mixtures of solvents to extract products selectively. As the products are frequently solvated, the release of solvent molecules such as S(CH_3_)_2_ has been confirmed by TGA/DSC or in-situ diffraction characterization. However, some materials cannot be dried without decomposing. Additionally, the emission of some B_2_H_6_ is also observed during heating to temperatures close to those of solvent release. Besides decomposition, the release of B_2_H_6_ could be related to occluded impurities from the synthesis process. Stadie et al. demonstrated that the elimination of solvents and B-H impurities with supercritical N_2_ processing (roughly, exposing to liquid N_2_ at 110 K and 2 MPa and degassing several times) practically eliminated B_2_H_6_ release from Mg(BH_4_)_2_ [196]. Another issue to be noticed is that the metathesis reaction between RECl_3_ and LiBH_3_ frequently leads to the formation of mixtures of RE(BH_4_)_3_, LiRE(BH_4_)_4_ and LiRE(BH_4_)_3_Cl [197]. The relative amount of these products might depend on synthesis conditions, such as the excess of reactants or milling time and temperature [198].

### 6.1. Lanthanides

**La**(**BH_4_**)**_3_** was produced by mechanical milling of anhydrous LaCl_3_ and LiBH_4_ using different milling times (1 to 48 h) [199]. Zhang et al. indicated that a similar reaction with LaF_3_ was not as responsive as with LaCl_3_ [199]. The dehydrogenation of these mixtures indicated a multistep process; the main dehydrogenation event occurred at approximately 523 K for the LaCl_3_ precursor and 573 K for LaF_3_ [199]. The difference was explained by the formation of La(BH_4_)_3_ in the first case and the in-situ reaction between LiBH_4_ and LaF_3_ in the second case [199]. In both cases, the dehydrogenation products were LaH_2_, LaB_6_, H_2,_ and LiCl or LiF, respectively. Standard dehydrogenation enthalpies were estimated as 48.2 and 49.3 kJ/mol H_2_, respectively [199]. Partial hydrogen uptake of the dehydrogenated materials was observed (at 623 K or 723 K, 10 MPa, 24 h) [199]. Recently, pure La(BH_4_)_3_ was produced by the reaction of (pre-milled) LaCl_3_ and LiBH_4_ in toluene with further solvent extraction with S(CH_3_)_2_ [200]. Using EtO_2_ as a solvent or by means of ball-milling, the mixed compound LiLa(BH_4_)_3_Cl was produced [200]. La(BH_4_)_3_·*n*S(CH_3_)_2_ released S(CH_3_)_2_ upon heating between 333 and 382 K and hydrogen at 531 K [200]. In general, LiLa(BH_4_)_3_X, X = Cl, Br, and I, can be produced and possess high Li-conductivity [201].

**NaLa**(**BH_4_**)**_4_** was obtained by the reaction between pure La(BH_4_)_3_ and NaBH_4_, induced by ball-milling [202]. However, the same procedure using LiBH_4_ does not give LiLa(BH_4_)_4_ [202]. Any remaining La(BH_4_)_3_ and NaBH_4_ continue to react between 355 and 463 K and 0.1 MPa argon pressure to form more NaLa(BH_4_)_4_ [202]. NaLa(BH_4_)_4_ crystallizes in orthorhombic space group *Pbcn* (Table 10) [202]. NaLa(BH_4_)_4_ decomposed in several steps, starting at 413–483 K with the release of the remaining solvent (dimethyl sulfide) and some diborane (as read from the TGS-DSC-MS plot in ref. [202]). The subsequent (proper) dehydrogenation reactions can be summarized as [202]:

NaLa(BH_4_)_4_ → La(BH_4_)_3_ + NaBH_4_, 463–483K (92)

La(BH_4_)_3_ → ½ LaH_2_ + ½ LaB_6_ + 11/2 H_2_, 538–623 K (93)

Partial reversibility was achieved by the re-formation of NaBH_4_ at 10 MPa hydrogen pressure, and up to 723 K [202].

**Figure 7:**
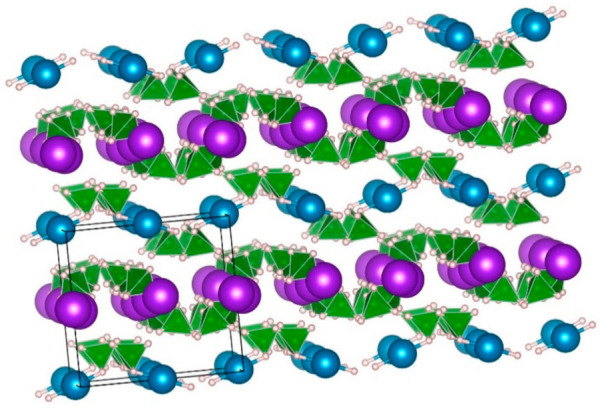
Crystal structure of K_3_La(BH_4_)_6_. Detailed crystal structure can be found in Table 10.

**K_3_La**(**BH_4_**)**_6_** was produced by the same procedure as NaLa(BH_4_)_4_, i.e., the addition reaction between La(BH_4_)_3_ and 3KBH_4_, assisted by ball-milling [202]. K_3_La(BH_4_)_6_ and NaLa(BH_4_)_4_ behave in a similar way [202]. K_3_La(BH_4_)_6_ crystallized in a monoclinic *P2_1_/n* (Table 10), double-perovskite structure (Figure 7), and also decomposed at 532–563 K into the precursor borohydrides (La(BH_4_)_3_ and KBH_4_) [202].

At these temperatures, the decomposition of La(BH_4_)_3_ is immediate (Equation (93)) [202]. Between 573 and 673 K, a reaction between LaH_2_ (the decomposition product of La(BH_4_)_3_) and KBH_4_ occurred:

½ LaH_2_ + 3KBH_4_ → 3KH + 1/2LaB_6_ + 5H_2_ (94)

Partial reversibility was also observed in this case, with the hydrogenation reaction being attributed to the reverse reaction of (94) at 10 MPa H_2_-pressure and 723 K [202].

**Li_3_K_3_La_2_**(**BH_4_**)**_12_** was observed due to the contamination with small quantities of LiBH_4_ during the synthesis of K_3_La(BH_4_)_6_ [202]. The crystal structure of Li_3_K_3_La_2_(BH_4_)_12_ was reported in [202] (Table 10). Li_3_K_3_La_2_(BH_4_)_12_ is a ionic Li^+^ conductor (6 × 10^−7^ S/cm) [203].

**Ce**(**BH_4_**)**_3_** was also produced by mechanical milling of anhydrous CeCl_3_ and LiBH_4_ for different milling times (1 to 48 h) [199]. The behavior of Ce(BH_4_)_3_ is very similar to La(BH_4_)_3_ [199]: CeF_3_ was not as reactive as LaCl_3_, and dehydrogenation of these mixtures indicates a multistep process; the main dehydrogenation event occurred at approximately 498 K for the CeCl_3_ precursor and 563 K for CeF_3_. The difference was also explained by the formation of Ce(BH_4_)_3_ in the first case, and the in-situ reaction between LiBH_4_ and CeF_3_ in the second case [199]. In both cases, the dehydrogenation products were CeH_2_, CeB_6_, H_2,_ and LiCl or LiF. Standard dehydrogenation enthalpies were estimated as 23.2 and 25.6 kJ/mol H_2_, respectively [199]. A partial hydrogen uptake of the dehydrogenated materials was observed (623 K or 723 K, 10 MPa, 24 h) [199]. Gennari et al. reported the production of Ce(BH_4_)_3_ by the mechanical ball-milling of CeCl_3_ + 3LiBH_4_ [205]. Ce(BH_4_)_3_ (plus LiCl) desorbed hydrogen in three endothermic events at approximately 503, 533, and 533 K (as read from DSC curves in [205]). The proposed global dehydrogenation products are CeH_2_, B or CeB_6,_ and H_2_, with Ce_2_(B_12_H_12_)_3_ as an intermediary [205]. Ce(BH_4_)_3_ was also produced in the same way as La(BH_4_)_3_, i.e., in toluene with further solvent extraction with S(CH_3_)_2_ [200]. Ce(BH_4_)_3_, as La(BH_4_)_3_, crystalizes in rhombohedral (r-) (Figure 3) and cubic (c-) polymorphs, Table 11 [200]. Ce(BH_4_)_3_ released ~6 wt% between 473 K and 573 K, with an endothermic peak at 524 K, and simultaneous H_2_ and B_2_H_6_ release [200]. **LiCe**(**BH_4_**)**_3_Cl** was produced as a by-product, and it is reported to have Li-ion conductivity [200].

**NaCe**(**BH_4_**)**_4_** was produced by mechanical milling of Ce(BH_4_)_3_ and NaBH_4_, and the milling was performed with careful control of the milling time and pauses [204]. Ce(BH_4_)_3_ was produced first as described above, and then used to produce NaCe(BH_4_). The NaCe(BH_4_)_4_ is isostructural to NaLa(BH_4_)_4_ [204] (Table 11). Heating of the ball-milled mixture of Ce(BH_4_)_3_ and NaBH_4_ induced the supplementary addition reaction between the (initially unreacted) Ce(BH_4_)_3_ and NaBH_4_ at approximately 373 K [204]. The maximum amount of NaCe(BH_4_)_4_ occurred at 446–460 K [204]. However, the presence of two unknown compounds that might be solvates of Ce-borohydride was reported [204]. The release of solvents (S(CH_3_)_2_), B_2_H_6_, and H_2_ occurred over a narrow temperature range, 453–498 K, with a second release of B_2_H_6_ and H_2_ at 523 K (as read from TGA-DSC-MS plots) [204]. The decomposition of NaCe(BH_4_)_4_ is understood to first form Ce- and Na-borohydrides [204].

**K_3_Ce**(**BH_4_**)**_6_** (*P2_1_/n* [22]) was briefly mentioned as a by-product of the synthesis of Li_3_K_3_Ce_2_(BH_4_)_12_ [203]. **Li_3_K_3_Ce_2_**(**BH_4_**)**_12_** is an ionic Li^+^ conductor; its bulk conductivity was reported to be nearly seven orders of magnitude higher (3 × 10^−7^ S/cm) than in Li_3_Tb_3_Te_2_O_12_ (Table 11) [203]. Its conductivity is explained by a “paddle-wheel” (combined size and ionic dynamical disorder) effect of the [BH_4_]^−1^ on Li^+^ [203]. Li substitution by Na, K, or Rb, Ce substitution by Ca, Sr or Eu, or [BH_4_]^−1^ substitution by Cl^-^ could modify the conductivity properties [203]. As stated in the introductory section, borohydrides might be an option in batteries, providing high ionic conductivity at room temperature. Thus, ionic conductivity is studied and data are reported recently [206]. However, we must perform a brief parenthesis to link ion conductivity (ion mobility) to dehydrogenation properties. Wu proposed that tuning the mobility of small ions can help to improve the hydrogen storage properties of metal hydride materials, as do particle size reduction, catalyst, or destabilization [207]. Luo et al. proposed that ionic conductivity is influenced by ionic radius and structure [206]. Wu describes that materials with a perovskite structure might accelerate hydrogen mobility [207], while Davies et al. [208] and Anderson et al. [209] suggest that halide substitution in amide materials improves ionic conductivity and hydrogen release properties. Unfortunately, not enough data on borohydrides are available to perform a correlation.

**Rb_3_Ce**(**BH_4_**)**_6_** (monoclinic *P2_1_/n*) was briefly mentioned in a recent report [69].

The formation of reactive hydride composites of Ce-Li was studied by Gennari et al. [210]. They produced, using mechanical milling, the following mixture [210]:

6LiBH_4_ + CeCl_3_ → Ce(BH_4_)_3_ + 3LiCl + 3LiBH_4_ (95)

The mixture decomposed in a multistep pathway starting at 493 K and going up to 673 K [210]. The mixture presented partial reversibility at 673 K and 6 MPa. After the second dehydrogenation, the products included CeB_6_, CeH_2_, CeB_4_, LiH, and H_2_, with the first two being confirmed by X-ray diffraction [210].

**Pr**(**BH_4_**)**_3_** was reported recently; this material was produced by the reaction of pre-milled PrH_3_ with S(CH_3_)BH_3_ in toluene, with further extraction/purification with DMS and drying [31]. LiPr(BH_4_)_3_Cl can also be produced during the synthesis of Pr(BH_4_)_3_ when using LiBH_4_ and PrCl_3_ as precursors [211]. Pr(BH_4_)_3_ presents different phases when heated under hydrogen pressure (4 MPa): α-, β-, β′-, β″-, and r- Pr(BH_4_)_3_ (Table 12). α-Pr(BH_4_)_3_ is stable at room temperature and isostructural to α-Y(BH_4_)_3_ and α-Gd(BH_4_)_3_. β-Pr(BH_4_)_3_ appeared along with α-Pr(BH_4_)_3_ during the drying of Pr(BH_4_)_3_·S(CH_3_)_2_, i.e., from the solvated material. β-Pr(BH_4_)_3_ is a high-pressure polymorph (4 MPa H_2_ pressure and 463 K) [31]. At ~449 K, β-Pr(BH_4_)_3_ transformed to β′-Pr(BH_4_)_3_, with contraction of the unit cell. At 463 K, a further contraction of the β′-phase occurred, producing β″-Pr(BH_4_)_3_ [31]. Further heating produces the disappearance of α-Pr(BH_4_)_3_ and the emergence of r-Pr(BH_4_)_3_. The decomposition of Pr(BH_4_)_3_ was registered at 520 K, with practically no B_2_H_6_ evolution; the reaction is [31]:

2Pr(BH_4_)_3_ → PrB_6_ + PrH_2_ + 11H_2_ (96)

**NaPr**(**BH_4_**)**_4_** was produced by the addition reaction between NaBH_4_ and halide-free Pr(BH_4_)_3_. The addition reaction was carried out by ball-milling, with careful control of the milling time/pause [204]. NaPr(BH_4_)_4_ crystallizes in an orthorhombic unit cell (*Pbcn*), and is isostructural to NaLa(BH_4_)_4_ (Table 12) [204]. In-situ diffraction studies during heating of the NaBH_4_ and Pr(BH_4_)_3_ mixture, i.e., NaPr(BH_4_)_4_, demonstrated the formation of two unidentified crystalline materials and the decrease in intensity of NaPr(BH_4_)_4_ XRD reflections at about 463 K (as read from an in-situ synchrotron radiation x-ray diffraction pattern) [204]. Further heating led to the decomposition of the unidentified materials. Partial reversibility (low hydrogenation levels over several cycles) was achieved in this material [204].

**Figure 8:**
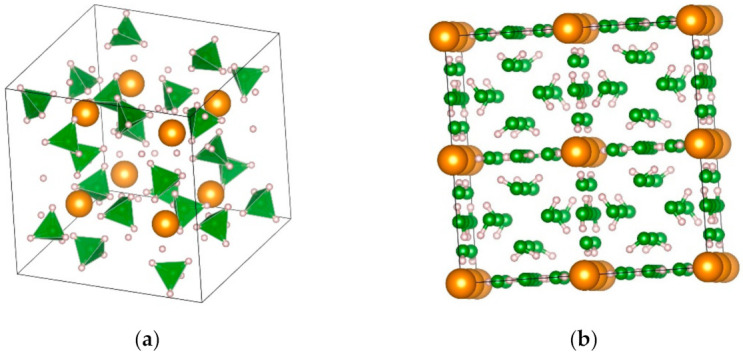
Crystal structure of (**a**) α-Nd(BH_4_)_3_ (isostructural to α-Y(BH_4_)_3_ and α-Gd(BH_4_)_3_ [31]); and (**b**) β-Nd(BH_4_)_3_ (isostructural to β-RE(BH_4_)_3_, RE = Ce, Sm, Ho, Y, Er, Tm, Yb).

**Nd**(**BH_4_**)**_3_** was produced by the reaction of pre-milled NdH_3_ with S(CH_3_)BH_3_ in toluene, with further extraction/purification with DMS and drying at 453 K under vacuum [31]. α-Nd(BH_4_)_3_ is isostructural to the room temperature polymorphs of α-Y(BH_4_)_3_ and α-Gd(BH_4_)_3_ [31], with cubic group symmetry *Pa-3,* and a = 11.2462(2) Å [17]. β-Nd(BH_4_)_3_ is also a high-pressure polymorph (543 K, 9.8 MPa) and is isostructural to β-RE(BH_4_)_3_, RE = Ce, Sm, Ho, Y, Er, Tm, Yb (Figure 8) [31]. Nd(BH_4_)_3_ also undergoes phase transitions when heated under pressure β- to β′- at 447–463 K, and then to -β″—463–543 K [31]. Non-solvated Nd(BH_4_)_3_ decomposed at approximately 523 K, in a reaction similar to (97) [17,31].

No Pm-borohydrides were located during the investigation for this review.

A few reports about **Sm**(**BH_4_**)**_2_** were located during the preparation of this review; in one report, SmCl_3_ reacted with LiBH_4_ (1:3) in anhydrous diethyl ether. During the reaction, Sm^+3^ was reduced to Sm^+2^ to form Sm(BH_4_)_2_. Adding S(CH_3_)_2_, and further drying/annealing allowed the production of non-solvated Sm(BH_4_)_2_ material above 438 K [212]. In another report, Sm(BH_4_)_2_ was produced by the reaction between SmH_2_ and THF·BH_3_ (1M) in THF for 48 h at 338 K [213]. In another type of synthesis, ball-milling/mixing of LiBH_4_ and SmCl_3_ (6:1) produced two Sm-borohydrides: α-**Sm**(**BH_4_**)**_3_** and β-Sm(BH_4_)_3_ [214].

Sm(BH_4_)_2_ crystallized in the orthorhombic *Pbcn* space group (Table 13) [17,212]. Sm(BH_4_)_2_ is isostructural to Sr(BH_4_)_2_ and Eu(BH_4_)_2_ [213]. It is worth mentioning that Eu and Sm can appear as divalent and trivalent cations, and some of their corresponding borohydrides have been reported. The structure of Sm(BH_4_)_2_ was described as [Sm(BH_4_)_6_] sharing-edges octahedra (with other two octahedra), building chains in the c-direction [214]. Each chain of octahedra is connected via corner-sharing to four others; each [BH_4_]^−1^ tetrahedra is surrounded by 3 Sm atoms in a distorted trigonal planar environment [214].

The decomposition of pure Sm(BH_4_)_2_ occurred at ~608 K, with no evidence of the formation of crystalline SmB_6_ [212]. In the 6:1 ball-milled mixture of LiBH_4_ and SmCl_3_ at 473 K, the reduction of trivalent borohydride to divalent borohydride with diborane release was proposed [214]. TG measurements of the 6:1 ball-milled mixture of LiBH_4_ and SmCl_3_ presented two mass loss events at approximately 448 K and 598 K [214]. Crystalline Sm(BH_4_)_2_ from the reduced ball-milled mixture decomposed at lower temperatures than the solvent-extracted material. This difference in decomposition temperatures was explained by the presence of LiCl and other Sm-compounds that modify/destabilize the Sm-borohydrides [212]. Additionally, in the 6:1 ball-milled mixture of LiBH_4_ and SmCl_3_, the formation of **LiSm**(**BH_4_**)**_3_Cl** as a secondary reaction between LiCl and Sm(BH_4_)_2_ was proposed upon heating at 453 K [214].

MSm(BH_4_)_3_ were produced by the ball-milling of Sm(BH_4_)_2_ and M(BH_4_)_3_ where, for M = K, the bimetallic borohydride was observed right after ball milling and, for M = Rb and Cs, further heating of the milled mixture led to the formation of the bimetallic borohydrides [213]. The MSm(BH_4_)_3_ compounds M = K, Rb, and Cs are isostructural to MSr(BH_4_)_3_; i.e., they have perovskite-type structures [213]. The similarities are attributed to similar cation size [213]. **CsSm**(**BH_4_**)**_3_** presented two second-order polymorphic transitions, the first one at 354 K, possibly related to the formation of α′-CsSm(BH_4_)_3_, and the second at ~618 K, involving the formation of β- CsSm(BH_4_)_3_, which only exists between 635 and 655 K [213]. All of the MSm(BH_4_)_3_ phases, M = K, Rb, and Cs, decomposed at T > 553 K, and the proposed reaction is [213]:

3MSm(BH_4_)_3_ → 3MBH_4_ + 2SmH_2_ + SmB_6_ + 10H_2_ (97)

Very importantly, partial rehydrogenation has been achieved for the MSm(BH_4_)_3_ materials by heating at 623 K and 19 MPa hydrogen pressure; the authors mentioned that it is probable that the original double-cation borohydrides were re-formed [213].

Recently, the study of Eu-borohydrides has gained attention due to the luminescence of these compounds. **Eu**(**BH_4_**)**_2_** can be produced by the reaction of freshly ball-milled EuH_2_ with triethylamine borane [215]:

EuH_2_ + 2(C_2_H_5_)_3_N-BH_3_ → Eu(BH_4_)_2_ + 2N(C_2_H_5_)_3_ (98)

The initial mixture was kept at 373 K overnight under reflux, and then at 418 K for 5 h [215]. After that, Eu(BH_4_)_2_ was extracted and dried. Eu(BH_4_)_2_ can also be prepared using ball-milling of LiBH_4_ and EuCl_2_ or EuCl_3_ with or without purification/solvent extraction [212]. Several polymorphs have been reported: *o*-Eu(BH_4_)_2_, which transforms to *t*-Eu(BH_4_)_2_ upon heating at 668 K and coexists with *c*-Eu(BH_4_)_2_ (Table 14) [215]. This last compound is considered (probably) metastable [215]. *t*-Eu(BH_4_)_2_ melts at 698 K. Humphries et al. identified an amorphization process at 461 K [212]. Eu(BH_4_)_2_ presents a single emission band at 463 nm [215]. Decomposition of Eu(BH_4_)_2_ at 668 K is reported as a complex process, with the initial thermal decomposition described as [215]:

2Eu(BH_4_)_2_ → Eu_2_(BH_4_)H_3_ + B_3_H_9_ (99)

Eu_2_(BH_4_)H_3_ melts at 698 K; its crystalline structure is reported in [215].

Another way to reduce the dehydrogenation temperature of actinide borohydrides is through the production of mixtures. For example, a ball-milled mixture of EuCl_3_ + 6LiBH_4_ (i.e., Eu(BH_4_)_2_, LiBH_4_, and LiCl) decomposed between approximately 348 and 398 K (as observed in a TG/DSC plot) [214].

The synthesis of **Eu**(**BH_4_**)**_3_** was achieved only recently, by way of mechanical milling at low temperatures, 77–273 K, and a large excess of LiBH_4_ [7]. Two polymorphs of Eu(BH_4_)_3_ were produced [7]:

EuCl_3_ + 3LiBH_4_ → α-Eu(BH_4_)_3_ + 3LiCl (100)

EuCl_3_ + 12LiBH_4_ → β-Eu(BH_4_)_3_ + 3LiCl + 9LiBH_4_ (101)

The Eu(BH_4_)_3_ polymorphs are unstable at room temperature, and compared to other RE(BH_4_)_3_ decomposition products; Eu(BH_4_)_3_ decomposition did not involve the formation of Eu(BH_4_)_2_. Wegner et al. explain this peculiarity as due to the redox potential of the Eu^+3^/Eu^+2^ pair [7].

Other compounds with Eu and [BH_4_]^−1^ ligands have been reported, but their existence was not corroborated due to poor characterization or synthesis details. Halide substitution was reported as early as 1966; EuBr(BH_4_) was produced by the reaction of EuBr_2_ (reduced in-situ from EuBr_3_) and LiBH_4_ in THF [216]. In a 1999 report, NaEu(BH_4_)_4_·4DME was used as a precursor to Eu(BH_4_)_2_ [217]. **CsEu**(**BH_4_**)**_3_** was reported recently (with few details of its synthesis); it shows intense blue luminescence centered around 485 nm, which is 20 nm red-shifted from Eu(BH_4_)_2_ [69]. **RbEu**(**BH_4_**)**_3_** was also briefly mentioned in a recent report as having luminescence properties [69].

Reports on gadolinium borohydrides indicated a rich chemistry, several different crystal structures (Table 15), and structural modifications by means of double cation or anion substituted materials. Ball-milling produced **Gd**(**BH_4_**)**_3_** (+3LiCl) presented two relevant endothermic processes upon heating: i) at 484 K (peak temperature), a phase transition to a high-temperature Gd(BH_4_)_3_ polymorph (cubic structure with a = 11.418 Å [218]). ii) Decomposition started at 503 K, with a peak temperature at 531 K [218]. Among the dehydrogenation products, GdB_4_ [7] and GdH_2_ were observed; thus, two dehydrogenation reactions were proposed [218]:

Gd(BH_4_)_3_ → 0.25GdH_2_ + 0.75GdB_4_ + 5.75H_2_ (102)

Gd(BH_4_)_3_ → GdH_2_ + 3B + 5H_2_ (103)

Solvated gadolinium borohydride, Gd(BH_4_)_3_·S(CH_3_)_2_, released S(CH_3_)_2_ upon heating at 408 K (peak temperature) [219], and then decomposed at 508 K [14]–543 K [17], with a small release of B_2_H_3_ [219].

Several double cation Gd-borohydrides have been reported: **NaGd**(**BH_4_**)**_4_** was formed after heat treatment of NaBH_4_-Gd(BH_4_)_3_; it was so amorphous and unstable that it decomposed at RT in one day [204]. **A_n_Gd**(**BH_4_**)**_n+3_, A = K, and Cs**, were produced by ball-milling mediated addition reactions between KBH_4_ or CsBH_4_ and Gd(BH_4_)_3_; the available crystallographic information is collected in Table 15 [20,69,204]. The structure of **Cs_3_Gd**(**BH_4_**)**_6_** is double-perovskite-type with luminescence properties [69].

Cation- and anion-substituted LiGd(BH_4_)_3_Cl can be produced as a by-product of mechanical milling and further heating to 466 K of GdCl_3_ and LiBH_4_ [14]:

Gd(BH_4_)_3_ + LiCl → LiGd(BH_4_)_3_Cl (104)

LiGd(BH_4_)_3_Cl possesses high Li-ion conductivity at 293 K; 3.5 × 10^−4^ S cm^−1^ [14]. Decomposition of LiGd(BH_4_)_3_Cl was reported at 534 K to form GdH_2_, GdB_4_, H_2,_ and LiCl. Essentially, there is no difference in dehydrogenation products as compared to other millings of GdCl_3_/LiBH_4_ or reactive mixtures.

The reactive mixture of Gennari et al., Gd(BH_4_)_3_ + 3LiBH_4_ + 3LiCl (from mechanical milled 6LiBH_4_ + GdCl_3_), present similarities with the analogous mixture with Ce; dehydrogenation is a multistep process, with hydrogen desorption occurring at 493 K, 548K, and 588K [210]. Among the dehydrogenation products, crystalline GdB_4_ and GdH_2_ were identified [210]. The proposed dehydrogenation pathway is, first, the decomposition of Gd(BH_4_)_3_ to produce GdH_2_, GdB_4,_ and H_2_; then, GdH_2_ reacts with LiBH_4_ to produce additional GdB_4_ and H_2_, as well as LiH [210]. In line with the possible formation of LiRE(BH_4_)_3_Cl during the ball-milling of 6:1 mixtures of LiBH_4_ and RECl_3_, Olsen et al. proposed another general dehydrogenation reaction occurring in this kind of reactive mixture [214]:

LiRE(BH_4_)_3_Cl + 3LiBH_4_ → LiCl + REB_6_ + 3LiH + (21/2)H_2_ (105)

Partial rehydrogenation of the dehydrogenated Gd(BH_4_)_3_ + 3LiBH_4_ + 3LiCl mixture was achieved at 673 K and 6 MPa [210].

For the late lanthanide borohydrides of RE = Tb, Dy, Ho, Er, Tb, Tm, Yb, and Lu, the synthesis is the same as that described above, i.e., pre-milled REH_3_ reacted with dimethyl sulfide-borane in toluene at 318 K for up to seven days, followed by solvent extraction [13]. Yet, mechanical milling of LiBH_4_ and RECl_3_ can also be an option, with or without temperature control [7]. The α- and β-RE(BH_4_)_3_ polymorphs share the same cubic group symmetry, α: *Pa-3,* and β: *Fm-3* [13], with smooth size changes of the crystallographic cell upon the cation radii [7]. In the mechanical-milling route, an excess of LiBH_4_ favors the formation of the β-RE(BH_4_)_3_ polymorph [7]. Wegner et al. reported that most of the RE(BH_4_)_3_ prepared by mechanical milling decomposed with the fasted rate at 523 K, producing very pure hydrogen [7]. The decomposition products of RE(BH_4_)_3_ have the formation of REB_4_ in common, except for the Lu-containing compound, which forms LuB_2_ [7].

Two Ho-borohydrides have been reported—**Ho**(**BH_4_**)**_3_** and **KHo**(**BH_4_**)**_4_** [220]. Ho(BH_4_)_3_ behaved like the usual RE(BH_4_)_3_ compounds described above, i.e., this material presents two polymorphs, α- and β-, whose relative amount depends on the excess of LiBH_4_ during the ball milling [220]. The peak decomposition temperature of 523 K was preceded by two smaller endothermic peaks [220]. KHo(BH_4_)_4_ was prepared by means of the solid-state reaction [220]:

HoCl_3_ + 3LiBH_4_ + KBH_4_ → KHo(BH_4_)_4_ + 3LiCl (106)

KHo(BH_4_)_4_ crystallizes in an orthorhombic lattice (*Cmcm*). Upon heating, this material melts at 448 K, and decomposition occurs at 531 K [220].

**Er**(**BH_4_**)**_3_** (from ball-milling ErCl_3_ + 3LiBH_4_, extraction with solvent and heat treatment) decomposed at 559 K (peak temperature), with low B_2_H_6_ emission [211]. Rehydrogenation at 613 K, 10 MPa hydrogen pressure during 21 h did not succeed [211]. **NaEr**(**BH_4_**)**_4_** was formed by the assisted ball-milled reaction between NaBH_4_ and Er(BH_4_)_3_ (crystal structure at Table 16) [204]. Melting was observed between 427 K and 435 K.

Yb (as in the case of Sm and Eu) can form **Yb**(**BH_4_**)**_2_** by the reduction in **Yb**(**BH_4_**)**_3_** [7]:

2Yb(BH_4_)_3_ → 2Yb(BH_4_)_2_ + B_2_H_6_ + H_2_ (107)

Ytterbium (II) borohydride has four reported polymorphs: α-Yb(BH_4_)_2_, α′-Yb(BH_4_)_2_, β-Yb(BH_4_)_2_, and γ-Yb(BH_4_)_2_ (Table 17) [17]. For their part, the Ytterbium (III) borohydrides α-Yb(BH_4_)_3_ and β-Yb(BH_4_)_3_ can be produced by mechanical milling of 3LiBH_4_ and YbCl_3_ [198]. α-Yb(BH_4_)_3_ is isostructural to the low temperature α-Y(BH_4_)_3_; however, β-Yb(BH_4_)_3_ is not similar to the high-temperature polymorph of Y(BH_4_)_3_ (Table 17) [198] because of a disordered [BH_4_]^−1^ orientation. α-Yb(BH_4_)_3_ and β-Yb(BH_4_)_3_ can coexist because the two modifications are close in energy [198]. Decomposition of Yb(BH_4_)_3_ occurred in two steps: the first one at 418 K and the second at 618 K (temperature of maximum gas evolution) [198]. As in other RE-borohydrides, chlorine substituted Yb-borohydrides have been reported: **LiYb**(**BH_4_**)**_4-x_Cl_x_** and **Yb**(**BH_4_**)**_2-x_Cl_x_** [198].

Mechanically milled LiYb(BH_4_)_4_ and NaYb(BH_4_)_4_ were briefly mentioned in a recent report; they presented lower Auzel´s crystal field strength properties as compared to Tb, Dy, Tm, and Ho-borohydrides [19]. **NaYb**(**BH_4_**)**_4_** and **KYb**(**BH_4_**)**_4_** were produced by a reaction analogous to (106); both compounds are isostructural to NaSc(BH_4_)_4_ and KY(BH_4_)_4_, i.e., orthorhombic *Cmcm* space group [221].

### 6.2. Actinides

Some of the actinide borohydrides were first synthesized during the Manhattan Project [222]. Today, the interest on actinide homoleptic borohydrides has notably decreased, yet they are summarized in this section. Actinide borohydrides, from Th to Pu, are volatile molecules near room temperature. Th(BH_4_)_4_, Pa(BH_4_)_4_, and U(BH_4_)_4_ are isomorphic, and their volatility increases with the increasing atomic number, whereas Np(BH_4_)_4_ and Pu(BH_4_)_4_ resemble the Zr-borohydride [223]. Additionally, Th, Pa, and U borohydrides have double-bridged [BH_4_]^−1^ groups that link metal atoms in a low-symmetry polymeric structure in the crystalline phase [223,224].

**Th**(**BH_4_**)**_4_** was produced in the Th-analogous reaction (109) at room temperature [72], and crystallized in the tetragonal *P4_3_2_1_2* space group. It is a polymeric material, melting at 476 K [222].

**Pa**(**BH_4_**)**_4_** was produced by the following reaction in the absence of solvent, followed by heating to 328 K, and recovered by condensation in a cold tramp at 273 K [222]:

PaF_4_ + 2Al(BH_4_)_3_*_(l)_* → Pa(BH_4_)_4_ + 2AlF_2_BH_4_ (108)

Pa(BH_4_)_4_ behaves like U(BH_4_)_4_ and Th(BH_4_)_4_ [222].

**U**(**BH_4_**)**_4_** was first produced during the Manhattan Project [225]. It can be prepared by stirring UCl_4_ and an excess of LiBH_4_ at room temperature for two weeks [81]. Old reports indicate that the reaction of UF_4_ with liquid Al(BH_4_)_3_ (Equation (109)) produces the uranium borohydride [226,227]. U(BH_4_)_4_, being volatile at room temperature, can be collected in a cold tramp and also purified in this way [81]. U(BH_4_)_4_ forms lustrous dark-green crystals; it is polymeric in the solid-state and monomeric in the gas phase [228]. U(BH_4_)_4_ crystallizes in the tetragonal *P4_3_2_1_2* space group, and its melting point is 399 K [222]. U(BH_4_)_4_ possesses high symmetry (*T_d_*), efficient screening of the metal atom, and the limited bridging ability of the [BH_4_]^−1^ ion [81]. The optical spectrum of U(BH_4_)_4_ can be revised at [223,227] and, relatively recently, the f-orbital covalency in U(BH_4_)_4_ was demonstrated [87]. U(BH_4_)_4_ is fairly stable at room temperature [229], but can decompose upon the action of ultraviolet photons of 253.5 nm (4.8 eV) at 295 K, with B_2_H_6_ being a decomposition product [228]:

2U(BH_4_)_4_ → 2U(BH_4_)_3_ + B_2_H_6_ + H_2_ (109)

or

U(BH_4_)_4_ → U(BH_4_)_2_ + B_2_H_6_ + H_2_ (110)

Thermal decomposition of U(BH_4_)_4_ was reported in 1981; Ghiassee et al. followed the decomposition of U(BH_4_)_4_, utilizing infrared spectroscopy in the gas phase [229]. U(BH_4_)_4_ decomposes thermally in two ranges of temperature: at 373 K, the proposed reaction is (109) [226,229,230]; at a temperature superior to 423 K, decomposition is rapid with the formation of a black-silver mirror [229]:

U(BH_4_)_4_ → U + 4B + 8H_2_ (111)

Decomposition kinetics was of first-order between 403 and 443 K and of second-order between 373 and 393 K [229].

**Np**(**BH_4_**)**_3_** is a dark green, pyrophoric, volatile, and reactive liquid that decomposes at 298 K [225], and whose physical properties resemble those of Hf and Zr borohydrides. Its optical spectrum was reported in detail in [231]. Np(BH_4_)_3_ was produced in a reaction type analogous to (108), but is unstable at room temperature, so that the reaction was carried out at 273 K [222]. Np(BH_4_)_3_ crystallizes in the tetragonal *P4_2_/nmc* space group; structural single-crystal studies were performed at 130 K. The melting point is 287 K and the boiling point is 426 K [222].

**Pu**(**BH_4_**)**_3_** is also an unstable and volatile compound; its synthesis was similar to that of Np(BH_4_)_3_ [222]. Pu(BH_4_)_3_ melts at 287 K and decomposes roughly at 293 K [225].

## 7. Thermodynamics (Thermal Stability)

As with any hydrogen storage material, formation enthalpy is the best predictor of a borohydride’s stability [232]. However, such information is only available for a small number of borohydrides. Because of that, other predictors have been considered. Nakamori et al. proposed an equation that relates the Pauling electronegativity, $\chi_{P}$, of the metal counter-ion with the formation enthalpy (per [BH_4_]^−1^ ion) [30,181]:

(112) $$\Delta H_{boro}=248.7\chi_{P}-390.8$$

**Figure 9:**
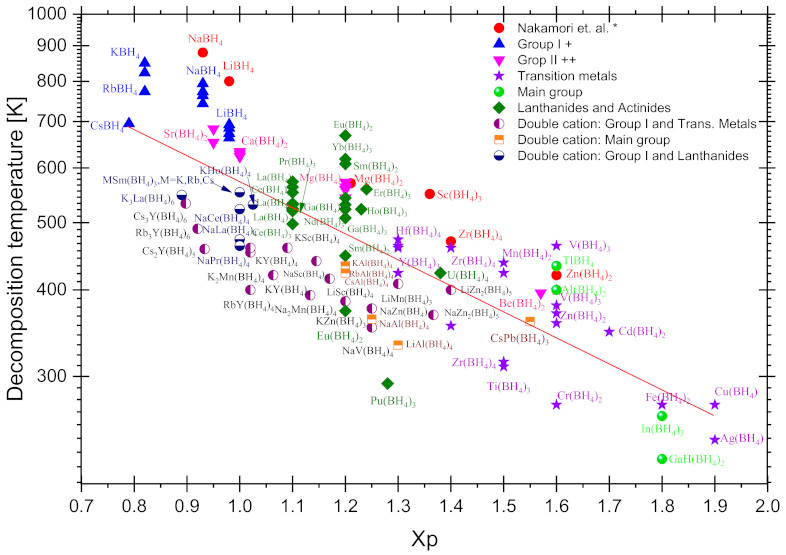
Compilation of dehydrogenation temperature (peak temperature whenever available, otherwise, onset temperature) versus Pauling electronegativity or weighted average Pauling electronegativity. Source data: * (red dots) Nakamori et al. (2006) [30]. + Group I borohydrides: LiBH_4_ [236,237,238,239,240], NaBH_4_ [241,242,243,244,245,246], KBH_4_ [4,247,248,249], RbBH_4_, and CsBH_4_ [50]. + + Group II borohydrides: Be(BH_4_)_2_ [250], Mg(BH_4_)_2_ [251,252,253,254], Ca(BH_4_)_2_ [255,256,257,258], and Sr(BH_4_)_2_ [215,259,260].

However, borohydrides can form double or even triple cation compounds. Thus, an adjustment must be made to Equation (112). In a series of ZrLi_m−4_(BH_4_)_m_, Li et al. proposed an average value of $\chi_{P}$ as a predictor of the dehydrogenation temperature [233]:

(113) $$\chi_{P}=\frac{1.4+1.0(m-4)}{1+(m-4)}$$

m = 0–4, in ZrLi_m−4_(BH_4_)_m_. However, an extension to other bi-cationic borohydrides is necessary. In Figure 9, we collected the dehydrogenation temperatures of the borohydrides (in logarithmic scale) encountered during the preparation of this review, and plotted as a function of the Pauling electronegativity. For bi-cation borohydrides of the general form M1_x_M2_y_(BH_4_)_z_, a weighted average was used:

(114) $$\chi_{P}=\frac{x*\chi_{PM1}+y*\chi_{PM2}}{x+y}$$

where $\chi_{PM1}$ and $\chi_{PM2}$ are the Pauling electronegativities of the respective cations in the borohydride. For visual reference, some previously reported data were included. Globally, a general tendency of reducing dehydrogenation temperature with the increase in cation electronegativity is confirmed. The red line in Figure 9 indicates the fitting of all collected data:

(115) $$\log T=3.1529-0.3859\chi_{p}$$

where $\chi_{P}$ is the Pauling electronegativity of the cation, or the average (Equation (114)) for bicationic borohydrides. The r-square of Equation (115) is rather low: 0.63866. However, it must be mentioned that there is no homogeneity in the way of reporting dehydrogenation temperatures; researchers have reported onset temperatures, peak temperatures, or ranges from the beginning and end of the reaction. Additionally, some (minor) fractions of the data were taken directly from the original plots in the reports; from DSC plots, for example. Moreover, data are reported from different techniques; for example, DSC, TGA, temperature-programmed desorption, and in-situ X-ray diffraction, and each technique shows differences in heating ramps or reaction atmospheres. Moreover, for the same borohydride, several temperatures of dehydrogenation have been reported, depending on different experimental parameters, particularly for the most studied Group I and II-borohydrides.

Other proposed predictors include the following: (i) The polarizing ability of the cationic bonding component [234], where the decreasing stability of the metal borohydride correlating with its increasing polarizing ability, as found in ab-inito studies [234]; and (ii) the relation between the Pauling electronegativity and the boron chemical NMR shift of metal borohydrides [235].

## 8. The Boron Problem

Particularly for hydrogen applications, the release of diborane and the formation of stable B-H species is a problem, as it compromises re-hydrogenation reactions. On the other hand, the release of hydrogen at low temperatures (from RT to roughly 373 K) is required for compatibility with PEM (polymer exchange membrane) fuel cell applications. However, diborane release is frequently associated with the low-temperature decomposition of borohydrides. The last facts seem entirely incompatible. Yet, along with the borohydrides listed in this review, several factors appear to influence the release of diborane. These factors include, but are not limited to, the synthesis method, purification, elimination of occluded molecules with supercritical N_2_ processing [196], thermal history of the material, storage conditions, temperature and pressure of dehydrogenation, existence (intentional or not) of other materials such as salts, hydrides or other borohydrides, etc. Among the consulted papers, different researchers reported from null or undetectable to high amounts of diborane release for the same material. Thus, several authors have proposed theories to understand the factors influencing diborane release. Diborane release can be explained in three ways: (i) Diborane is released but decomposes at high temperatures (before being detected); (ii) diborane is an intermediary in the formation and decomposition of borohydrides; and (iii) diborane is not emitted. [261].

The accepted explanation of diborane emission during dehydrogenation reactions is that the amount of B_2_H_6_ released scales inversely with borohydride stability, which depends on the corresponding cation’s electronegativity [262]. Callini et al. mentioned that the difference in electronegativity results in a charge transfer from the cation to the anion that destabilizes and distorts the [BH_4_]^−1^ units [262]. [BH_4_]^−1^ distortion can be detected and quantified utilizing infrared spectroscopy; Callini et al. proposed the stretching span (Δs) as a way to classify B_2_H_6_-releasing borohydrides. In this approach, the stretching span is the difference in energies of the stretching vibrations of hydrogen atoms with different bonding lengths [262]. In this model, a threshold of Δs > 200 cm^−1^ is proposed as the predictor of B_2_H_6_-releasing borohydrides; materials such as LiSc(BH_4_)_4_, Zr(BH_4_)_4_, and Al(BH_4_)_3_ will release B_2_H_6_, while LiBH_4_ will not [262]. Yet, LiBH_4_ releases B_2_H_6_ under several reaction conditions [262]. Harrison et al. mentioned that diborane production did not follow any clear trend but, for threshold values between 1.36 and 1.55 of electronegativity of the corresponding metal in the Pauling scale, $\chi_{P}$, diborane production is observed [182].

Furthermore, the dehydrogenation mechanism is frequently unresolved for transition metal borohydrides, in contrast to Group I and II borohydrides, where the formation of intermediaries such as M_x_(B_12_H_12_) M = Li, K, Rb, Cs has been identified [263,264,265]. However, some models and reactions of B-H compounds can be used to explain the dehydrogenation reaction. In the model of Callini, the distorted [BH_4_]^−1^ unit is the precursor for the formation of (H^δ−^)_2_ and BH_2_^δ+^, with the formation of diborane proposed as [262]:

2M^δ1+^ + 2(BH_4_)^δ1−^ → 2M^δ1+^ + (H^δ−^)_2_ + BH_2_^δ1+^BH_4_^δ1−^ → 2M + H_2_ + B_2_H_6_ (116)

Other B–H compounds or ions have been reported from the reaction between B_2_H_6_ and borohydrides or hydrides in the context of studying the borane chemistry [266]:

B_2_H_6_ + MBH_4_ → MB_3_H_8_ + H_2_ (117)

B_5_H_9_ + MH → M(B_5_H_8_) + H_2_ (118)

M(B_5_H_8_) + ½B_2_H_6_ → M(B_6_H_11_) (119)

B_10_H_14_ + MBH_4_ → MB_11_H_14_ + 2H_2_, M = Li, Na (120)

Reaction (117) requires a temperature of about 373 K to proceed at a reasonable rate [266]. Meanwhile, Equations (118)–(120) correspond to different reactions for a growing B–H cluster [266]. Those reactions were performed in solvent; however, some of these reactions might occur during heating of the metal borohydrides. Indeed, the formation of [B_3_H_8_]^−^ intermediaries in Y borohydride and [B_12_H_12_]^2−^ in Sc borohydride [235] (and references within) has been reported. Other possible intermediaries include [B_10_H_12_]^2−^ and [B_11_H_14_]^−^ [267,268]. For its part, the rapid dissociation of a BH_4_^•^ radical into BH_3_ an H^•^ above 250 K was predicted by means of ab-initio MO calculations [269].

It is also worth mentioning that, during the dehydrogenation of borohydrides or their composites, the formation of MB_x_ species seems to favor re-hydrogenation [270,271]. In turn, the increase in back pressure during dehydrogenation favors the formation of MB_x_ compounds [271]. Thus, a competition between the formation of metal boride compounds and B_2_H_6_ might be established.

Strategies for reducing the release of B-H compounds include increasing the hydrogen back-pressure during dehydrogenation [269], modification of the dehydrogenation pathway by using, for example, the formation of reactive composites [269], the use of catalysts (such as Mo(100) surface) for the decomposition of diborane [261], or other B-H intermediaries, etc.

Nano- and meso-confinement could be beneficial; until now, this was mostly employed for Group I borohydrides [272]. For example, Xia et al. reported the complete suppression of the release of B_x_H_y_ by-products and a reduction in the decomposition temperature (a decrease of about 50 K) in SBA-15- confined NaZn(BH_4_)_3_ [159]. Stabilization of volatile Ti(BH_4_)_3_ was achieved by nano-confinement in MOF UiO-66 (Zr_6_O_4_(BDC)_6_, BDC =1,4-benzenedicarboxylate) [76]. In this way, Ti(BH_4_)_3_-UiO-66 was stabilized at room temperature under an argon atmosphere and, during dehydrogenation, there was no emission of diborane [76]. Instead, the decomposition involved B_5_H_9_ that recombined at an increased temperature to form solid higher boranes [76].

Finally, other essential data of [BH_4_]^−1^ must be mentioned: the electronegativity of [BH_4_]^−1^ is considered as 2.13 [22,108], and its electron affinity is 3.18 eV [188], while its ionic size is 2.03 Å [22].

## 9. Conclusions and Perspectives

Beyond Groups I and II, borohydrides present interesting properties related to chemistry, structure, hydrogen release, and, in an emerging trend, ionic conductivity and photoluminescence. Among the materials reviewed in the present work the transition metals and lanthanide borohydrides stand out.

Several strategies have been tried to generate new bimetallic and trimetallic borohydrides with tailored decomposition temperatures and mechanisms of diborane release in materials intended for hydrogen storage. However, many of these new materials decompose into the “parent” borohydrides, and then high temperatures are required to release a substantial amount of hydrogen. Thus, decomposition of double or triple cation borohydrides into the “parent” borohydrides is not recommendable in the hydrogen storage area. Re-hydrogenation is still to be reached in most revised compounds. The ideal procedure would be a one-step decomposition into simple (and re-hydrogenable) compounds. To achieve this goal, several routes, such as the addition of catalysts, the use of different ligands (take advantage of organometallic chemistry), or the formation of ammonia–borane compounds, are still to be explored or are under exploration.

Additionally, the nanoconfinement of borohydrides into MOFs, polymers, zeolites, etc., could soon be a strategy for the development of new composite materials. Otherwise, porous borohydrides can be used for the confinement of H_2_ or other small molecules.

Anion substitution is another opportunity area that should be thoroughly studied because of the possibilities for generating new materials with high ionic conductivity for battery applications. Along with this review, we observed the formation of several Cl-substituted borohydrides, i.e., their synthesis is facile and their niche application can be developed.

## Figures and Tables

**Table 1:** Infrared and Raman bands typically observed in covalent metal–borohydrides [21].

Coordination Modes of [BH_4_]^−1^	Frequency [cm^−1^] and Type of Vibration
η^1^	2300–2450: B-H(terminal) stretching ~2000: B-H(bridge) stretching ~2000–1700: M-H(bridge) stretching 1000–1150: BH_3_ deformation
η^2^	2400–2600: B-H(terminal) stretching 1650–2150: B-H(bridge) stretching 1300–1500: Bridge stretching 1100–1200: BH_2_ deformation
η^3^	2450–2600: B-H(terminal) stretching 2100–2200: B-H(bridge) stretching 1200–1250: Bridge deformation data

**Table 2:** Crystal structures of Sc-borohydrides.

Material and Hydrogen Content [wt.%]	Structural Parameters [Å, °]	Atomic Positions	Reference and Comments
Sc(BH_4_)_3_, 13.51	Space group: trigonal *R-3* (148) a = b = 7.262; c = 18.194 α = β = 90; γ = 120	**Sc:** 0, 0, 0.3210. **B:** 0.3503, 0.3123, 0.4079. **H1:** 0.4002, 0.3671, 0.3423. **H2:** 0.3691, 0.4611, 0.4432. **H3:** 0.1636, 0.1647, 0.4130. **H4:** 0.4588, 0.2370, 0.4329	Theoretical calculation [30]
	Space group: orthorhombic *C222_1_* (20) a = 8.34, b = 11.94; c = 7.90 α = β = γ = 90	**Sc:** 0.1882, 0, 0. **B1:** 0.3254, 0.1593, 0.9642. **B2:** 0.5, 0.4978, 0.25. **H1:** 0.4045, 0.2411, 0.9457. **H2:** 0.3191, 0.0983, 0.8355. **H3:** 0.3770, 0.0950, 0.0770. **H4:** 0.1854, 0.1775, 0.0036. **H5:** 0.1167, 0.0597, 0.2407. **H6:** 0.5192, 0.4369, 0.3732	Theoretical calculation [44]
LiSc(BH_4_)_4_ 14.49	Space group: tetragonal *P-42c* (112) a = b = 6.0759(1); c = 12.0338(1) α = β = γ = 90	**Li:** 0, 0, 0.104(2). **Sc:** 0.5, 0.5, 0.25. **B:** 0.75, 0.6722(6), 0.6386(3). **H1:** 0.7939, 0.6688, 0.7251. **H2:** 0.8960, 0.6926, 0.5891. **H3:** 0.6394, 0.8072, 0.6234. **H4:** 0.6705, 0.5201, 0.6166	Refinement of high-resolution synchrotron powder diffraction and comparison to DFT calculations [42]
	Space group: tetragonal *I-4* (82) a = b = 6.479; c = 12.043 α = β = γ = 90	**Li:** 0, 0.5, 0.25. **Sc:** 0, 0, 0.5. **B:** 0.3534, 0.2469, 0.8889. **H1:** 0.7621, 0.6206, 0.3339. **H2:** 0.5416, 0.2551, 0.8711. **H3:** 0.2852, 0.4221, 0.8705. **H4:** 0.3325, 0.2111, 0.9890	Theoretical calculation [44]
NaSc(BH_4_)_4_ 12.67	Space group: orthorhombic *Cmcm* (63) a = 8.170(2), b = 11.875(3), c = 9.018(2). α = β = γ = 90	**Na:** 0, 0, 0.5. **Sc:** 0.5, 0.1462(7), 0.75. **B1:** 0.5, 0.255(1), 0.957(2). **H11:** 0.5, 0.309(1), 1.060(2). **H12:** 0.5, 0.163(1), 0.989(2). **H13:** 0.613(2), 0.274(1), 0.889(2). **B2:** 0.724(2), 0.036(1), 0.75 **H21:** 0.837(2), −0.020(1), 0.75. **H22:** 0.762(2), 0.127(1), 0.75. **H23:** 0.649(2), 0.017(1), 0.852(2)	Experimental synchrotron powder diffraction [43]
	Space group: orthorhombic *C222_1_* (20) a = 8.318, b = 11.827, c = 9.117 α = β = γ = 90	**Na:** 0.07322, 0, 0. **Sc:** 0, 0.34577, 0.25. **B1:** −0.49941, 0.26614, −0.4569. **B2:** 0.27125, 0.04152, −0.25031. **H1:** −0:49327, 0.32151, 0.43233. **H2:** 0.39351, 0.29602, −0.37049. **H3:** 0:12897, 0.23042, 0.38793. **H4:** −0.35012, 0.48568, −0.25753. **H5:** 0.24323, 0.14288, −0.27103. **H6:** 0.3702, 0.01058, −0.34298. **H7:** 0.16075, 0.46621, 0.3703. **H8:** 0.47365, 0.16592, −0.4863	Theoretical calculation [45]
KSc(BH_4_)_4_ 11.24	Space group: orthorhombic *Pnma* (62) a = 11.8558(47), b = 7.7998(34); c = 10.1258(63) α = β = γ = 90	**K:** 0.1947(16), 0.25, 0.6527(17). **Sc:** 0.0640(16), 0.25, 0.2152(15). **B1:** 0.1647(32), 0.25, 0.0150(23). **H11:** 0.2342(48), 0.25, 0.0939(74). **H12:** 0.2050(75), 0.25, −0.0881(39). **H13:** 0.1097(33), 0.37018(21), 0.0271(41). **B2:** 0.3819(21), 0.25, 0.3840(45). **H21:** 0.3790(37), 0.25, 0.2706(45). **H22:** 0.2917(28), 0.25, 0.4251(69). **H23:** 0.4286(26), 0.37018(21), 0.4201(43). **B3:** 0.0901(15), 0.0137(21), 0.3431(18). **H31:** 0.080(13), −0.1071(38), 0.4069(53). **H32:** 0.058(12), −0.0137(51), 0.2383(28). **H33:** 0.183(10), 0.052(18), 0.339(10). **H34:** 0.0386(90), 0.124(22), 0.3886(94)	Experimental data [49]
RbSc(BH_4_)_4_ 8.50	Space group: orthorhombic *Pbcm* (57) a = 7.6514, b = 11.1821, c = 11.2443 α = β = γ = 90	**B1:** 0.23519, 0.02670, −0.58184. **H11:** 0.36413, 0.04382, −0.64732. **H12:** 0.27076, −0.02575, −0.49111. **H13:** 0.17172, 0.12656, −0.55910. **H14:** 0.12514, −0.02621, −0.64381. **H21:** 0.94449, 0.21868, −0.83832. **H32:** 0.22785, 0.29877, −0.66164. **Rb1:** 0.55522, 0.25, −0.5. **Sc1:** 0.16021, 0.13182, −0.75. **B2:** 0.86828, 0.17635, −0.75. **H22:** 0.71314, 0.19760, −0.75. **H23:** 0.89853, 0.06708, −0.75. **B3:** 0.32462, 0.30552, −0.75. **H31:** 0.41624, 0.21334, −0.75. **H33:** 0.41306, 0.39492, −0.75	Computationally optimized [50]
CsSc(BH_4_)_4_ 6.80	Space group: monoclinic *P2_1_/c* 14 a = 9.5870, b = 10.7270, c = 12.2280 α = 90, β = 126.3510, γ = 90	**Cs22:** 0.82694, 0.3427, 0.75062. **B1:** 0.21177, 0.01695, 0.27563. **H2:** 0.23203, 0.12082, 0.32887. **H3:** 0.15239, −0.05817, 0.31159. **H4:** 0.11686, 0.03374, 0.15052. **H5:** 0.3566, −0.01335, 0.30707. **B6:** 0.59468, 0.22457, 0.38529. **H7:** 0.53578, 0.28562, 0.27965. **H8:** 0.74057, 0.25675, 0.47509. **H9:** 0.48976, 0.23768, 0.41267. **H10:** 0.58853, 0.11248, 0.35595. **B11:** 0.13689, 0.33158, 0.1196. **H12:** 0.20824, 0.31906, 0.24318. **H13:** 0.03689, 0.41819, 0.07083. **H14:** 0.25418, 0.34173, 0.10504. **H15:** 0.06144, 0.23137, 0.06534. **B16:** 0.33930, 0.08443, 0.04155. **H17:** 0.39476, 0.19319, 0.07405. **H18:** 0.35291, 0.04517, −0.04403. **H19:** 0.42198, 0.02115, 0.14752. **H20:** 0.18734, 0.08764, 0.00469. **Sc21:** 0.32111, 0.1628, 0.20685	Computationally optimized [50]

**Table 3:** Crystal structures of Y-borohydrides.

Material and Hydrogen Content [wt.%]	Structural Parameters [Å, °]	Atomic Positions	Reference and Comments
α-Y(BH_4_)_3_ 9.06	Space group: cubic *Pa-3* (205) a = b = c = 10.894 α = β = γ = 90	**Y:** 0.2165, 0.2165, 0.2165. **B:** 0.1920, 0.2475, 0.9671. **H1:** 0.2900, 0.2540, 0.0241. **H2:** 0.1030, 0.2245, 0.0340. **H3:** 0.1737, 0.3481, 0.9181. **H4:** 0.2018, 0.1626, 0.8923	Theoretical calculation (predicted DFT) [53]. Isostructural to Gd(BH_4_)_3_ and Dy(BH_4_)_3_
	Space group: cubic *Pa-3* (205) a = b = c= 10.8522(7) α = β = γ = 90	**Y:** 0.2187(5), 0.2187(5), 0.2187(5). **B:** 0.1908(5), 0.2455(8), 0.9659(6). **D1:** 0.2849(6), 0.2525(8), 0.0272(7). **D2:** 0.1024(7), 0.2215(8), 0.0334(6). **D3:** 0.1781(7), 0.3450(7), 0.9189(7). **D4:** 0.1920(7), 0.1626(7), 0.8961(7)	Experimental [58]
β-Y(BH_4_)_3_ 9.06	Space group: cubic *Fm-3c* (226) a = b = c = 11.0086(1) α = β = γ = 90	**Y:** 0, 0, 0. **B:** 0, 0, 0.25. **D1:** 0, 0.4075(1), 0.3104(1)	Experimental, heat treatment in D at 10MPa and 475 K [58]
LiY(BH_4_)_4_ 10.39	Space group: tetragonal *P-42c* (112) a = b = 6.2360(9); c = 12.491(3) α = β = γ = 90	**B:** 0.7453, 0.7453, 0.6535. **H1:** 0.7642, 0.7596, 0.7527. **H2:** 0.8643, 0.8647, 0.6063. **H3:** 0.5562, 0.7854, 0.6331. **H4:** 0.7818, 0.5566, 0.6307. **Li**: 0, 0, 0.25. **Y**: 0.5, 0.5, 0.25.	[62]
NaY(BH_4_)_4_ 9.42	Space group: orthorhombic *C222_1_* (20) a = 8.5263(4), b = 12.1357(5), c = 9.0535(4) α = β = γ = 90_	**B1**: 0.4983, 0.2701, −0.0300. **B2**: 0.7375, 0.0357, 0.7498. **H11**: 0.5007, 0.3256, 0.0800. **H12:** 0.4682, 0.1735, 0.0018. **H13:** 0.6262, 0.2717, −0.0945. **H14**: 0.6025, 0.3000, 0.6200. **H21:** 0.3559, 0.4813, 0.7445. **H22**: 0.7652, 0.1337, 0.7245. **H23**: 0.6732, 0.0308, 0.8723. **H24:** 0.6408, 0.0026, 0.6589. **Na1:** −0.0604, 0, 0. **Y1:** 0, 0.3478, 0.25.	[62]
m-KY(BH_4_)_4_ 8.61	Space group: monoclinic *C2/c* (15) a = 14.8947(18), b = 7.8012(10), c = 8.1130(10) α = γ = 90.00, β = 110.167(2)	**Y:** 0.5, 0.7966(5), 0.25. **K:** 0.25, 0.25, 0. **B1:** 0.1338(13), 0.881(2), 0.767(2). **H11:** 0.078(10), 0.90(5), 0.625(7). **H21:** 0.104(16), 0.94(4), 0.872(10). **H31:** 0.21(2), 0.95(7), 0.78(2). **H41:** 0.15(4), 0.733(13), 0.79(2). **B2:** 0.0792(11), 0.5812(18), 0.108(3). **H12:** 0.146(5), 0.58(4), 0.238(9). **H22:** 0.102(10), 0.62(3), −0.011(10). **H32:** 0.02(2), 0.68(4), 0.123(18). **H42:** 0.05(3), 0.44(2), 0.082(15)	460 K [68]
o-KY(BH_4_)_4_ 8.61	Space group: orthorhombic *Cmcm* (63) a = 8.59314(10), b = 12.59917(15), c = 9.78460(12) α = β = γ = 90	**K:** 0, 0, 0.5. **Y:** 0.5, 0.13211(7), 0.75. **B1:** 0.5, 0.2416(4), 0.9513(6). **H11:** 0.6074(10), 0.2595(4), 0.8887(8). **H13:** 0.5, 0.2924(6), 1.0464(11). **H14:** 0.5, 0.1550(9), 0.9813(6). **B2:** 0.7244(5), 0.0127(4), 0.75. **H21:** 0.8318(11), −0.0390(6), 0.75. **H22:** 0.7602(6), 0.0990(9), 0.75. **H23:** 0 0.6529(9), −0.0045(4), 0.8443(9)	420 K [68]
o-RbY(BH_4_)_4_ 6.90	Space group: orthorhombic *Pnma* (62) a = 12.3406(3), b = 8.2482(2), c = 10.5934(3) α = β = γ = 90	**Rb:** 0.1816(3), 0.25, 0.6609(5). **Y:** 0.0415(3), 0.25, 0.1771(6). **B1:** 0.056(3), 0.027(3), 0.315(3). **H11:** 0.06(4), −0.084(12), 0.386(10). **H12:** 0.06(3), −0.022(14), 0.211(7). **H13:** 0.13(4), 0.11(7), 0.330(14). **H14:** −0.02(3), 0.10(8), 0.336(13). **B2:** 0.212(3), 0.25, 0.070(4). **H21:** 0.214(19), 0.25, 0.181(7). **H22:** 0.167(7), 0.367(6), 0.033(11). **H23:** 0.302(9), 0.25, 0.03(2). **B3:** 0.389(4), 0.25, 0.454(4). **H31:** 0.364(14), 0.25, 0.346(8). **H32:** 0.440(5), 0.367(6), 0.476(10). **H33:** 0.310(10), 0.25, 0.517(15)	400 K [68]
c-Rb_3_Y(BH_4_)_6_ 5.57	Space group: cubic *Fm-3* (202) a = b = c = 11.5998(3) α = β = γ = 90	**Rb:** 0.31, 0.25, 0.25. **Y1a** (**Y**)**:** 0.04, 0, 0. **Y1b** (**Rb**)**:** 0.04, 0, 0. **Y2a** (**Rb**)**:** 0.5, 0.5, 0.5. **Y2b** (**Y**)**:** 0.5, 0.5, 0.5. **B:** 0.5, −4.930381e−032, 0.269(6) **H:** 0.580(18), 0.00000017(4), 0.325(14)**H:** 0.5, −0.080(18), 0.212(14)	490 K, cubic [68]
Cs_3_Y(BH_4_)_6_ 4.19	Space group: cubic *Fm-3* (202) a = b = c = 12.2541(2) α = β = γ = 90	**Cs:** 0.31, 0.25, 0.25. **Y1a** (**Y**)**:** 0.029(8), 0, 0. **Y1b** (**Cs**)**:** 0.029(8), 0, 0. **Y2a** (**Cs**)**:** 0.452(3), 0.5, 0.5. **Y2b** (**Y**)**:** 0.452(3), 0.5, 0.5. **B:** 0.5, 0, 0.254(11). **H:** 0.575(14) 0.00000016(3) 0.308(15). **H:** 0.5, −0.075(14), 0.201(15)	553 K [68]
Rb_2_LiY(BH_4_)_6_ 6.80	Space group: cubic *Fm-3* (202) a = b = c = 11.44541(7) α = β = γ = 90	**Rb:** 0.25, 0.25, 0.25. **Y1a** (**Y**)**:** 0, 0, 0. **Y1b** (**Li**)**:** 0, 0, 0. **Li2a** (**Li**)**:** 0.5, 0.5, 0.5. **Li2b** (**Y**)**:** 0.5, 0.5, 0.5. **B:** 0.5, 0, 0.2605(7). **H:** 0.584(3), 0.000000176(7), 0.320(2). **H:** 0.5, −0.084(3), 0.201(2)	415 K [68]
Cs_2_LiY(BH_4_)_6_ 5.37	Space group: cubic *Fm-3* (202) a = b = c = 11.25215(19) α = β = γ = 90	**Cs:** 0.25, 0.25, 0.25. **Y:** 0, 0, 0. **Li:** 0.5, 0.5, 0.5. B:0.5, 0, 0.2508(2). **H:** 0.5824(5), 0.0000001720(11), 0.3091(4). **H:** 0.5, −0.0824(5), 0.1925(4), 0.5	Room temperature [68]

**Table 4:** Molecular parameters of Ti-borohydride.

Material and Hydrogen Content [wt.%]	Symmetry	Atomic Positions [Å]	Comments
Ti(BH_4_)_3_ 13.09	*C3h*	**Ti:** 0, 0, 0. **B1:** 0.14648, 1.91483, −0.00744. **B2:** −1.61184, −0.84502, 0.00457. **B3:** 1.53773, −0.97338, 0.00253. **H1:** −1.10173, 1.64998, −0.00544. **H2:** 0.67735, 1.51811, 1.08297. **H3:** 0.67563, 1.50978, −1.09559. **H4:** −0.78907, −1.82032, 0.00764. **H5:** −1.61691, −0.23520, 1.12541. **H6:** −1.61875, −0.24383, −1.12091. **H7:** 1.97099, 0.22681, −0.00244. **H8:** 1.01724, −1.27698, 1.1273. **H9:** 1.01546, −1.28564, −1.11902. **H10:** 0.22774, 3.07799, −0.01194. **H11:** −2.65980, −1.35621, 0.00740. **H12:** 2.50443, −1.62533, 0.00429	Atomic positions were generated by symmetry arguments from selected bond distances reported in [71]

**Table 5:** Crystal structures of Zr-borohydrides.

Material and Hydrogen Content [wt.%]	Structural Parameters [Å, °]	Atomic Positions	Reference and Comments
Zr(BH_4_)_4_ 10.71	Space group: cubic *P-43m* (215)a = b = c = 5.8387(4) α = β = γ = 90	**Zr:** 1, 1, 1. **B:** 0.7714(2), 0.7714(2), 0.7714(2). **H1**: 0.668(3), 0.668(3), 0.668(3). **H2:** 0.956(4), 0.748(2), 0.748(2)	At 100 K [83]

**Table 6:** Crystal structures of Hf-borohydrides.

Material and Hydrogen Content [wt.%]	Structural Parameters [Å, °]	Atomic Positions	Reference and Comments
Hf(BH_4_)_4_ 6.78	Space group: cubic *P-43m* (215)a = b = c = 5.8387(4) α = β = γ = 90	**Hf:** 0, 0, 0. **B:** 0.226(2), 0.226, 0.226. **H1:** 0.340(5), 0.340, 0.340. **H2:** 0.258(2), 0.258, 0.019(2)	At 100 K [93]

**Table 7:** Crystal structures of Mn-borohydrides.

Material and Hydrogen Content [wt.%]	Structural Parameters [Å, °]	Atomic Positions	References and Comments
α-Mn(BH_4_)_2_ 9.53	Space group: trigonal *P3_1_12* (151) a = 10.4349(1), c = 10.835(2) α = β = 90, γ = 120	**Mn1:** 0.23130(50), 0.91807(72), 0.12532(40). **Mn2:** 0.56272(32), 2x, 0.6666667. **B1:** 0.0403(34), 0.6990(30), 1.0056(58). **H11:** −0.0824(33), 0.6582(70), 1.0014(67). **H12:** 0.0712(79), 0.6455(79), 0.9292(92). **H13:** 0.0685(66), 0.6689(49), 1.0980(89) **H14:** 0.1041(50), 0.8233(31), 0.9937(44). **B2:** 0.4708(31), 2x, 0.1666667. **H21:** 0.4285(44), 1.0140(63), 0.206(41). **H22:** 0.393(40), 0.8688(61), 0.0914(60). **B3:** 2y, 0.10206, 0. **H31:** 0.1323(30), 0.1145(36), −0.0717(24). **H32:** 0.2766(30), 0.0642(23), −0.0463(37). **B4:** 0.1329(17), 1-x, 0.3333333. **H41:** 0.0199(28), 0.8265(39), 0.3754(44). **H42:** 0.1250(54), 0.7875(42), 0.2588(29). **B5:** 0.7056(23), 1-x, 0.8333333. **H51:** 0.7531(36), 0.4149(28), 0.8590(98). **H52:** 0.716(11), 0.232(10), 0.9146(22)	[115]
δ-Mn(BH_4_)_2_ 9.53	Space group: tetragonal *I4_1_/acd* (142) a = 7.85254(6), b = 7.85254(6),c = 12.14548(17) α = β = γ = 90	**Mn:** 0, 0.25, 0.125. **B:** 0.0197(17), 0, 0.25, **H1:** −0.0833(17), −0.01726, 0.17047. **H2:** 0.1247(17), 0.86217, 0.24890	[111]
δ’-Mn(BH_4_)_2_ 9.53	Space group: orthorhombic *Fddd* (70) a = 12.638(15), b = 9.321(10), c = 9.205(17) α = β = γ = 90	**Mn:** −0.08981, 0.125, 0.125, **B1:** 0.52633, 0.44943, 0.21955. **H11:** 0.55862, 0.32065, 0.21651. **H12:** 0.31244, 0.30784, 0.26337. **H13:** 0.17630, 0.20909, 0.29581. **H14:** 0.53619, 0.49567, 0.09304	[111]
γ-Mn(BH_4_)_2_ 9.53	Space group: cubic *Ia-3d* (230) a = b = c= 16.2094(13) α = β = γ = 90	**Mn:** ¼, 1/8, ½. **B1:** 0.3090(11), 0.0590(11), 3/8. **H1:** 0.2849(11), 0.0199(11), 0.43730. **H2:** 0.2966(11), 0.1347(11), 0.38560	[109]
K_2_Mn(BH_4_)_4_ 8.38	Space group: monoclinic *P2_1_/c* (14) a = 8.1375(7), b = 9.8456(7), c = 12.7420(12) α = 90, β = 100.552(6), γ = 90	**K1:** 0.2813(8), 0.3548(5), 0.5547(6). **K2:** 0.1974(11), 0.5047(7), 0.1980(5). **Mn1:** 0.7793(6), 0.2180(3), 0.4143(5). **B1:** 0.495(3), 0.676(3), 0.638(2). **H11:** 0.454(6), 0.74(2), 0.56(1). **H12:** 0.458(9), 0.73(2), 0.71(1). **H13:** 0.64(1), 0.66(1), 0.653(4). **H14:** 0.43(2), 0.57(2), 0.63(1). **B2:** 0.503(3), 0.270(2), 0.811(2). **H21:** 0.558(6), 0.241(5), 0.735(3). **H22:** 0.578(5), 0.213(6), 0.886(3). **H23:** 0.361(6), 0.239(7), 0.799(3). **H24:** 0.516(9), 0.389(2), 0.826(4). **B3:** 0.866(3), 0.357(3), 0.578(2). **H31:** 0.948(8), 0.354(7), 0.51(1). **H32:** 0.76(1), 0.436(8), 0.556(6). **H33:** 0.95(1), 0.388(8), 0.660(6). **H34:** 0.808(9), 0.248(5), 0.587(5). **B4:** 0.210(3), 0.524(2), 0.918(2). **H41:** 0.195(7), 0.449(3), 0.844(4). **H42:** 0.345(6), 0.513(8), 0.97(1). **H43:** 0.11(2), 0.498(9), 0.971(6). **H44:** 0.19(1), 0.637(7), 0.886(6)	[108]
Li_3_MnZn_5_(BH_4_)_15_ 9.67	Space group: hexagonal *P6_3_/mcm* (193) a = 15.391(3), c = 8.590(2) α = β = 90, γ = 120	**Zn1:** 1/3, 2/3, ¼. **Zn2:** 0.2861(4), 0, ¼. **Li/Mn:** 0.6089(7), 0, ¼. **Li:** 0, 0, 0. **B1:** 0.131(1), 0, ¼. **H11:** 0.089(2), 0, 0.358(2). **H12:** 0.139(2), −0.069(2), ¼. **B2:** 0.332(2), 0, 0.002(2). **H21:** 0.296(2), 0, −0.113(2). **H22:** 0.405(2), 0, −0.021(2). **H23:** 0.279(2), −0.069(2), 0.071(2). **B3:** 0.525(1), 0.344(2), ¼. **H31:** 0.443(2), 0.284(2), ¼. **H32:** 0.532(2), 0.420(2), 1/4. **H33:** 0.563(2), 0.336(2), 0.142(2)	Refined from synchrotron radiation powder diffraction data at room temperature [129]

**Table 8:** Crystal structures of Zn-borohydrides.

Material and Hydrogen Content [wt.%]	Structural Parameters [Å, °]	Atomic Positions	References and Comments
Zn(BH_4_)_2_ 8.48	Space group: triclinic *P-1* (2) a = 6.877, b = 5.440, c =7.842 α = 89.5, β = 76.15, γ = 89.98	**Zn:** 0.2498, 0.0001, 0.9998. **B1:** 0.0567, 0.8903, 0.7939. **B2:** 0.5497, 0.1754, 0.8166. **H1:** 0.0997, 0.8534, 0.6382. **H2:** 0.5892, 0.2640, 0.6710. **H3:** 0.1789, 0.7718, 0.8612. **H4:** 0.3316, 0.7331, 0.0999. **H5:** 0.1031, 0.2267, 0.1397. **H6:** 0.3863, 0.2679, 0.9011. **H7:** 0.0498, 0.1135, 0.8210. **H8:** 0.4481, 0.0506, 0.1928	Theoretical [30]
	Space group: orthorhombic *Pmc2_1_* (26) a = 4.118, b = 4.864, c = 7.916 α = β = γ = 90	**Zn:** 0, 0.28459, 0.0089. **B1:** 0.5, −0.06481, 0.4706. **B2:** 0, −0.46194, 0.24842. **H1:** 0.5, −0.30735, 0.42745. **H2:** 0.27449, −0.0050, −0.42983. **H3:** 0, 0.39706, 0.37973. **H4:** 0, 0.21066, −0.24017. **H5:** 0.25820, −0.48360, −0.32151. **H6:** 0.5, 0.08396, 0.3488	Optimized structure [144]
LiZn(BH_4_)_3_ 10.35	Space group: monoclinic *P2_1_/c* (14) a = 10.59, b = 14.74, c = 8.66 α = γ = 90, β = 111.056	**Zn1:** 0.76982, 0.11283, 0.7512. **Zn2:** 0.40867, 0.26754, 0.60812. **Li1:** 0.08413, 0.12904, 0.25099. **Li2:** 0.77464, 0.09463, 0.25701. **B1:** 0.51747, 0.39654, 0.64566. **B2:** 0.24290, 0.26206, 0.72420. **B3:** 0.84715, 0.05769, 1.01282. **B4:** 0.84433, −0.00056, 0.57012. **B5:** 0.53022, 0.13104, 0.62602. **B6:** 0.87262, 0.25874, 0.80977. **H11:** 0.48781, 0.46308, 0.70437. **H12:** 0.60520, 0.40322, 0.58737. **H13:** 0.41470, 0.3777, 0.52066. **H14:** 0.5496, 0.33635, 0.75538. **H21:** 0.12228, 0.25280, 0.65129. **H22:** 0.27910, 0.33967, 0.71248. **H23:** 0.29189, 0.19988, 0.6632. **H24:** 0.27506, 0.25791, 0.37092. **H31:** 0.81526, 0.01114, 0.88397. **H32:** 0.78587, 0.12997, 0.9924. **H33:** 0.96820, 0.07003, 1.05745. **H34:** 0.81149, 0.00813, 1.10142. **H41:** 0.76794, −0.04277, 0.62085. **H42:** 0.79042, 0.00405, 0.4204. **H43:** 0.95622, −0.03529, 0.61027. **H44:** 0.87034, 0.07826, 0.62903. **H51**: 0.43883, 0.07855, 0.59858. **H52:** 0.50460, 0.19684, 0.52625. **H53:** 0.61326, 0.09429, 0.5739. **H54:** 0.57432, 0.15595, 0.77025. **H61:** 0.95757, 0.20272, 0.87941. **H62:** 0.77207, 0.22980, 0.69158. **H63:** 0.91771, 0.31006, 0.73238. **H64:** 0.83042, 0.29690, 0.90608	DFT optimization [155]
LiZn_2_(BD_4_)_5_ 9.51 (calculated as H)	Space group: orthorhombic *Cmca* (64) a = 8.6031(13), b = 17.8876(4), c = 15.3598(3) α = β = γ = 90	**Zn1:** 0, 0.6440(10), 0.7665(11). **Zn2:** 0, 0.4252(12), 0.6300(16). **Li1:** 0, 0.138(6), 0.434(6). **B1:** 0, 0.2580(4), 0.3166(5). **D11:** 0, 0.1912(4), 0.3101(17). **D12:** 0, 0.2807(10), 0.3903(5). **D13:** 0.1153(7), 0.2798(7), 0. 2804(7). **B2:** 0, 0.3513(4), 0.0903(5). **D21:** 0, 0.2971(5), 0.0442(8). **D22:** 0, 0.4090(5), 0.0505(9). **D23:** 0.1136(7), 0.3488(10), 0.1355(5). **B3:** 0, 0.5320(4), 0.7016(4). **D31:** 0, 0.4746(5), 0.7421(9). **D32:** 0, 0.5264(14), 0.6238(5). **D33:** 0.1145(7), 0.5662(5), 0.7223(9). **B4:** 0.2284(8), 0.3825(4), 0.5882(4). **D41:** 0.1551(14), 0.4234(6), 0.5415(7). **D42:** 0.3368(10), 0.4186(7), 0.6142(11). **D43:** 0.1591(14), 0.3629(10), 0.6520(6). **D44:** 0.2550(19), 0.3297(5), 0.5424(8)	295 K, ^11^B [156]
NaZn(BH_4_)_3_ 9.10	Space group: triclinic P1 (1) a = 7.125, b = 7.246, c = 4.688 α = 99.254 β = 91.097 γ = 71.422	**Li:** −0.353, 0.225, −0.465. **Zn:** −0.022, −0.471, 0.277. **B:** −0.139, −0.150, 0.339. **B:** 0.257, 0.353, 0.442. **B:** −0.241, 0.396, 0.047. **H:** −0.052, −0.034, 0.428. **H:** −0.045, −0.248, 0.112. **H:** −0.134, −0.247, −0.46. **H:** −0.31, −0.086, 0.276. **H:** 0.343, 0.18, 0.356. **H:** 0.251, 0.439, 0.229. **H:** 0.092, 0.359 −0.465. **H:** 0.328, 0.438, −0.363. **H:** −0.282, 0.485, 0.298. **H:** −0.069, 0.374, −0.041. **H:** −0.242, 0.227, 0.036. **H:** −0.355, 0.495, −0.107	PEGS + DFT calculations [146]
NaZn_2_(BH_4_)_5_ 8.84	Space group: monoclinic *P2_1_/c* (14) a = 9.397(2), b = 16.635(3), c = 9.1359(16) α = γ = 90, β = 112.658(19).	**Na1:** 0.245(6), 0.436(3), 0.117(8). **Zn1:** 0.2873(16), 0.7660(11), 0.643(3). **Zn2:** 0.8412(17), 0.6268(8), 0.395(2). **B1:** 0.5212, 0.3234, 0.2606. **B2:** 0.7102, 0.0954, 0.3551. **B3:** 0.0844, 0.6934, 0.5422. **B4:** 0.7766, 0.5874, 0.6112. **B5:** 0.7766, 0.9126, 0.6654. **H11:** 0.3982, 0.3357, 0.1991. **H12:** 0.5824, 0.3869, 0.2912. **H13:** 0.5530, 0.2145, 0.6707. **H13:** 0.5530, 0.2855, 0.3823. **H21:** 0.6098, 0.1394, 0.3049. **H22:** 0.8140, 0.1363, 0.4070. **H23:** 0.7076, 0.4452, 0.7476. **H23:** 0.7076, 0.0548, 0.4600. **H31:** 0.9880, 0.7394, 0.4940. **H32:** 0.0428, 0.6250, 0.5214. **H33:** 0.1548, 0.7038, 0.6833. **H33:** 0.1548, 0.7962, 0.9715. **H41:** 0.8304, 0.5354, 0.5677. **H42:** 0.8574, 0.6148, 0.7372. **H43:** 0.7370, 0.6415, 0.5129. **H44:** 0.6750, 0.5617, 0.6206. **H51:** 0.8304, 0.9646, 0.7627. **H52:** 0.8574, 0.8852, 0.6202. **H53:** 0.7370, 0.8585, 0.7241. **H54:** 0.6750, 0.9383, 0.5544	[147]
KZn(BH_4_)_3_ 8.12	Space group: trigonal R3 (146) a = b = 7.6291(8), c = 10.977(2)α = γ = 90, β = 120	**K:** 0, 0, 0.49936. **Zn:** 0, 0, 0.00309. **B:** 0.31006, 0.05088, 0.01153. **H1:** 0.19196, 0.98384, 0.10009. **H2:** 0.44232, 0.21786, 0.03989. **H3:** 0.36275, 0.92929, 0.98755. **H4:** 0.22942, 0.06605, 0.91529	Atomic parameters at T = 100 K [160]
Li_3_MgZn_5_(BH_4_)_15_ 10.17	Space group: hexagonal *P6_3_/mcm* (193) a = b = 15.371(3), c = 8.586(2) α = γ = 90, β = 120	**Zn1:** 1/3, 2/3, ¼. **Zn2:** 0.2832(5), 0, ¼. **Li/Mg:** 0.598(1), 0, ¼. **Li:** 0, 0, 0. **B1:** 0.131(1), 0, ¼. **H11:** 0.089(2), 0, 0.358(2). **H12:** 0.139(2), −0.069(2), ¼. **B2:** 0.338(1), 0, 0.004(2). **H21:** 0.302(2), 0, −0.111(2). **H22:** 0.410(2), 0, −0.019(2). **H23:** 0.285(2), −0.069(2), 0.073(2). **B3:** 0.5286(6), 0.346(2), ¼. **H31:** 0.447(2), 0.286(2), ¼. **H32:** 0.535(2), 0.423(2), ¼. **H33:** 0.566(2), 0.338(2), 0.143(2)	At RT. Occupation of the Li/Mg site Li: occ = 0.66(2), Mg: (1 − occ) = 0.34(2) Occupation of the Li site Li: 3*occ − 1 = 1 [129]

**Table 9:** Crystal structures of Al-borohydrides.

Material and Hydrogen Content [wt.%]	Structural Parameters [Å, °]	Atomic Positions	References and Comments
α-Al(BH_4_)_3_ 16.91	Space group: monoclinic *C2/c* (15) a = 21.917(4), b = 5.9860(12), c = 21.787(4) α = 90, β = 111.90(3), γ = 90	**Al:** 0.3795(2), 0.5901(7), 0.8361(2). **B1:** 0.3206(8), 0.3030(32), 0.8238(9). **H1A:** 0.3757(22), 0.2993(63), 0.8366(46). **H1B:** 0.3013(20), 0.4814(73), 0.8173(47). **H1C:** 0.3027(47), 0.2727(183), 0.8584(38). **H1D:** 0.3046(48), 0.2249(160), 0.7796(31). **B2:** 0.3866(8), 0.7476(31), 0.7508(8). **H2A:** 0.4169(33), 0.5987(109), 0.7778(30). **H2B:** 0.3557(34), 0.8117(91), 0.7806(28). **H2C:** 0.3642(41), 0.7334(182), 0.7017(23). **H2D:** 0.4205(40), 0.8679(136), 0.7655(48). **B3:** 0.4304(8), 0.7262(30), 0.9308(8). **H3A:** 0.3763(23), 0.7595(127), 0.9008(25). **H3B:** 0.4537(21), 0.6121(126), 0.9038(25). **H3C:** 0.4382(48), 0.6799(171), 0.9768(28). **H3D:** 0.4517(46), 0.8796(97), 0.9376(49)	[176] CCDC (Cambridge Crystallographic Data Centre) identification number 230830
β-Al(BH_4_)_3_ 16.91	Space group: orthorhombic *Pna2* (33) a = 18.021(3), b = 6.138(2), c = 6.1987(14) α = β = γ = 90	**Al:** 0.86775(6), 0.1592(2), 0.20350(9). **B1:** 0.7780(5), 0.0040(15), 0.0503(15). **H1:** 0.8398(18), −0.0226(76), 0.0215(80). **H1B:** 0.7744(19), 0.1321(67), 0.1790(80). **H1C:** 0.7576(29), −0.1277(74), 0.1214(93). **H1D:** 0.7568(32), 0.0678(98), −0.0833(79). **B2:** 0.9113(5), 0.0203(17), 0.4915(15). **H2:** 0.9320(23), −0.0107(69), 0.3238(56). **H2B:** 0.8667(21), 0.1458(67), 0.4853(78). **H2C:** 0.9534(26), 0.0912(84), 0.5627(93). **H2D:** 0.8887(32), −0.1166(69), 0.5420(101). **B3:** 0.9122(5), 0.4460(15), 0.0629(21). **H3A:** 0.8716(23), 0.4427(57), 0.1979(76). **H3B:** 0.9300(25), 0.2732(53), 0.0287(83). **H3C:** 0.9542(26), 0.5038(87), 0.1428(94). **H3D:** 0.8912(33), 0.4975(103), −0.0728(77)	[176] CCDC (Cambridge Crystallographic Data Centre) identification number 230829
Li_4_Al_3_(BH_4_)_13_ 17.37	Space group: cubic *P-43n* (218) a = b = c = 11.3640(3) Å α = β = γ = 90	**Li:** 0.1315(11), 0.1315(11), 0.1315(11). **B1:** 0, 0, 0. **H11:** 0.9424(5), 0.9424(5), 0.9424(5). **Al:** ¼, ½, 0. **B2:** 0.1731(5), 0.6680(5), 0.4547(4). **H21:** 0.1892(15), 0.6358(15), 0.3636(8). **H22:** 0.1595(15), 0.7671(6), 0.4548(15). **H23:** 0.0876(10), 0.6266(14), 0.4893(15). **H24:** 0.2463(11), 0.6455(15), 0.5176(12)	At 100 K Rietveld refinement [186]
KAl(BH_4_)_4_ 12.85	Space group: orthorhombic Fddd (70) a = 9.7405(3), b = 12.4500(4), c = 14.6975(4) α = β = γ = 90	**K:** 0.125, 0.125, 0.125. **Al:** 0.375, 0.375, 0.375. **B:** 0.7760(4), 0.6939(3), 0.7263(3). **H1:** 0.6927(10), 0.7372(10), 0.6864(8). **H2:** 0.7559(12), 0.7164(11), 0.7984(5). **H3:** 0.7654(12), 0.6077(5), 0.7198(8). **H4:** 0.8767(8), 0.7204(8), 0.7007(8)	[185]

**Table 10:** Crystal structures of La-borohydrides.

Material and Hydrogen Content [wt.%]	Structural Parameters [Å, °]	Atomic Positions	Comments
NaLa(BH_4_)_4_ 7.29	Space group: orthorhombic *Pbcn* (60) a = 6.79865, b = 17.31073,c = 7.26547 α = β = γ = 90.00	**Na:** 0.50044, 0.07200, 0.75028. **La:** 0.00006, 0.17197, 0.24971. **B:** 0.26676, 0.43014, 0.88057. **H1:** 0.34758, 0.36477, 0.88347. **H2.** 0.24611, 0.45458, 1.03337. **H3:** 0.10366, 0.42625, 0.80070. **H4:** 0.37496, 0.47548, 0.79356. **B:** 0.75157, 0.19400, 0.51886. **H1:** 0.63613, 0.20423, 0.38704. **H2:** 0.70323, 0.13930, 0.60198. **H3:** 0.74575, 0.25066, 0.62265. **H4:** 0.92434, 0.18235, 0.45697	[202]. NaLa(BH_4_)_4_ is isostructural to NaCe(BH_4_)_4_ and NaPr(BH_4_)_4_ [204]
K_3_La(BH_4_)_6_ 7.01	Space group: monoclinic *P2_1_/n* (14) a = 7.93840, b = 8.35246, c = 11.57068 α = γ = 90, β = 90.18977	**K1:** 0.5, 0.5, 0.5. **K2:** 0.47777, 0.05175, 0.25048. **La:** 0, 0, 0.5. **B1:** 0.34697, 0.83332, 0.0777. **H1:** 0.40173, 0.79562, 0.16487. **H2:** 0.28607, 0.95646, 0.08622. **H3:** 0.45213, 0.83873, 0.01072. **H4:** 0.24803, 0.74232, 0.04903. **B2:** 0.16081, 0.32239, 0.07051. **H1:** 0.09016, 0.29934, 0.15464. **H2:** 0.23328, 0.43994, 0.07762. **H3:** 0.25285, 0.21998, 0.05400. **H4:** 0.06681, 0.33015, −0.00418. **B3:** 0.63098, 0.43325, 0.22717. **H1:** 0.72435, 0.37192, 0.28787. **H2:** 0.55241, 0.52310, 0.27813. **H3:** 0.70283, 0.49845, 0.15596. **H4:** 0.54446, 0.33942, 0.18673	[202]
Li_3_K_3_La_2_(BH_4_)_12_ 8.14	Space group: cubic *Ia-3d* (230) a = 17.60563, b = 17.60563, c = 17.60563 α = β = γ = 90	**K:** 0.25, 0.8750, 0.5. **Li:** 0.3750, 0, 0.25. **La:** 0.5, 0, 0. **B:** 0.4, 0.7, 0.28. **H1:** 0.36161, 0.75067, 0.28790. **H2:** 0.45847, 0.71997, 0.26308. **H3:** 0.40365, 0.66722, 0.33496. **H4:** 0.37619, 0.66215, 0.23408	[202]

**Table 11:** Crystal structures of Ce-borohydrides.

Material and Hydrogen Content [wt.%]	Structural Parameters [Å, °]	Atomic Positions	Comments
r-Ce(BH_4_)_3_ 6.55	Space group: trigonal *R-3c* (167) a = b = 7.3745(1), c = 20.1567(2) α = β = 90, γ = 120	**Ce:** 0, 0, 0. **B:** 0.632(2), 0, ¼. **H1:** 0.461(2), −0.094(5), 0.261(2). **H2:** 0.759(2), 0.026(5), 0.288(2)	[200]. Isostructural to r-La(BH_4_)_3_
c-Ce(BH_4_)_3_ 6.55	Space group: cubic *Fm-3c* (226) a = b = c = 11.7106(6) α = β = γ =90	**Ce:** 0, 0, 0. **B:** 0, 0, ¼. **H:** 0, 0.4075(1), 0.3104(1)	[200]. Isostructural to c-La(BH_4_)_3_
NaCe(BH_4_)_4_ 7.25	Space group: orthorhombic *Pbcn* (60) a = 6.8028(5), b = 17.5181(13), c = 7.2841(5) α = β = γ =90	**Na1:** 0.50044, 0.0720, 0.75028. **Ce2:** 0.00006, 0.17197, 0.24971. **B3:** 0.26676, 0.43014, 0.88057. **H14:** 0.34758, 0.36477, 0.88347. **H15:** 0.24611, 0.45458, 0.03337. **H16:** 0.10366, 0.42625, 0.80070. **H17:** 0.37496, 0.47548, 0.79356. **B8:** 0.75157, 0.1940, 0.51886. **H19:** 0.63613, 0.20423, 0.38704. **H110:** 0.70323, 0.13930, 0.60198. **H111:** 0.74575, 0.25066, 0.62265. **H112:** 0.92434, 0.18235, 0.45697	[204]
Li_3_K_3_Ce_2_(BH_4_)_12_ 8.11	Space group: cubic *Ia-3d* (230) a = 17.60756(4), b = 17.60756(4), c = 17.60756(4) α = β = γ = 90	**Ce:** 0.5, 0, 0. K: 0.25, 0.8750, 0.5. **B:** 0.39971, 0.70333, 0.28374. **H13:** 0.3513, 0.68574, 0.32305. **H14:** 0.40777, 0.76754, 0.28684. **H15:** 0.45434, 0.67373, 0.30222. **H16:** 0.38538, 0.68622, 0.22287. **Li:** 0.3750, 0, 0.25	[203]

**Table 12:** Crystal structures of Pr-borohydrides.

Material and Hydrogen Content [wt.%]	Structural Parameters [Å, °]	Atomic Positions	References and Comments
α-Pr(BH_4_)_3_ 6.52	Space group: cubic *Pa-3* (205) a = b = c = 11.2941(5) α = β = γ = 90	**Pr:** 0.2179, 0.2179, 0.2179. **B:** 0.1930, 0.2473, 0.9682. **H13:** 0.2909, 0.2534, 0.0243. **H14:** 0.1043, 0.2257, 0.0351. **H15:** 0.1752, 0.3475, 0.9192. **H16:** 0.2014, 0.1623, 0.8942	[31]
β-Pr(BH_4_)_3_ β´-Pr(BH_4_)_3_ β´´-Pr(BH_4_)_3_ 6.52	Space group: cubic *Fm-3c* (226) a = b = c = 11.458(2) a = b = c = 11.3283(6) a = b = c = 11.1438(2) α = β = γ = 90	**Pr:** 0, 0, 0. **B:** 0, 0, 0.25. **H13:** 0, 0.4069, 0.3116	[31]
r -Pr(BH_4_)_3_ 6.52	Space group: trigonal *R-3c* (167) a = b = 7.373(6), c = 19.89(2) α = β = 90, γ = 120	**Pr:** 0, 0, 0. **B:** 0.6202(2), 0, 0.25. **D13:** 0.4835(4), −0.1056(10), 0.2881(1). **D14:** 0.7699(3), 0.1072(8), 0.2834(1)	[31]
NaPr(BH_4_)_4_ 7.22	Space group: orthorhombic *Pbcn* (60) a = 6.7617(2), b = 17.4679(7), c = 7.2523(3) α = β = γ = 90.00	**Na:** 0.50044, 0.0720, 0.75028. **Pr:** 0.00006, 0.17197, 0.24971. **B:** 0.26676, 0.43014, 0.88057. **H14:** 0.34758, 0.36477, 0.88347. **H15:** 0.24611, 0.45458, 0.03337. **H16:** 0.10366, 0.42625, 0.8007. **H17:** 0.37496, 0.47548, 0.79356. **B8:** 0.75157, 0.1940, 0.51886. **H19:** 0.63613, 0.20423, 0.38704. **H110:** 0.70323, 0.13930, 0.60198. **H111:** 0.74575, 0.25066, 0.62265. **H112:** 0.92434, 0.18235, 0.45697	[204]

**Table 13:** Crystal structures of Sm-borohydrides.

Material and Hydrogen Content [wt.%]	Structural Parameters [Å, °]	Atomic Positions	Comments
Sm(BH_4_)_2_ 4.48	Space group: orthorhombic *Pbcn* (60) a = 6.97129(14), b = 8.43870(17), c = 7.56841(14) α = β = γ = 90	**Sm:** 0, 0.15216(14), 0.25. **B:** 0.2544(14), 0.3710(18), 0.4218(14). **H1:** 0.384(4), 0.292(4), 0.369(6). **H2:** 0.153(6), 0.292(5), 0.514(7). **H3:** 0.164(7), 0.421(6), 0.300(4). **H4:** 0.316(6) 0.479(5) 0.506(7)	[212]

**Table 14:** Crystal structures of Eu-borohydrides.

Material and Hydrogen Content [wt.%]	Structural Parameters [Å, °]	Atomic Positions	References and Comments
*o*-Eu(BH_4_)_2_ 4.44	Space group: orthorhombic *Pbcn* (60) a = 6.90343(16), b = 8.37272(18), c = 7.48321(16) α = β = γ = 90	**Eu:** 0, 0.15042(20), 0.25. **B:** 0.2459(21), 0.3837(31),0.4335(21). **H1:** 0.327(11), 0.280(8), 0.352(11). **H2:** 0.138(11), 0.325(10), 0.540(10). **H3:** 0.155(12), 0.465(9), 0.332(11). **H4:** 0.363(10), 0.464(9), 0.509(13)	[212]
*t*-Eu(BH_4_)_2_ 4.44	Space group: tetragonal *P4_1_2_1_2* (92) a = 5.4091(6), b = 5.4091(6), c = 11.6201(17) α = β = γ = 90	**Eu1:** 0.0627(16), 0.0624(16), 0. **B2:** 0.9401, 0.4249, 0.3648. **H3:** 0.0998, 0.4164, 0.4199. **H4:** 0.9590, 0.58450, 0.3101. **H5:** 0.9311, 0.2550, 0.3159. **H6:** 0.7705, 0.4436, 0.41330	[215]
*c*-Eu(BH_4_)_2_ 4.44	Space group: cubic *Fm-3m* (225) a = b = c = 7.0602(17) α = β = γ = 90	**Eu1:** 0, 0, 0. **B2:** 0.25, 0.25, 0.25. **H3:** 0.15789, 0.15716, 0.34228	[215]

**Table 15:** Crystal structures of Gd-borohydrides.

Material and Hydrogen Content [wt.%]	Structural Parameters [Å, °]	Atomic Positions	Reference and Comments
α-Gd(BH_4_)_3_ 5.99	Space group: cubic *Pa-3* (205) a = b = c = 11.008 α = β = γ = 90	**Gd:** 0.2169, 0.2169, 0.2169. **B:** 0.1919, 0.2475, 0.9670. **H1:** 0.2892, 0.2539, 0.0231. **H2:** 0.1039, 0.2248, 0.0335. **H3:** 0.1736, 0.3472, 0.9186. **H4:** 0.2012, 0.1633, 0.8931	Theoretical calculation (predicted DFT). [53]
K_2_Gd(BH_4_)_5_ 6.51	Space group: monoclinic *P2_1_/m* (11) a = 8.7001(3), b = 12.1241(5), c = 11.9893(5) α = 90, β = 105.009(1), γ = 90	**K1:** 0.5957(9), 0.5399(6), 0.3382(7). **K2:** 0.8353(14), 0.7500, 0.6833(11). **K3:** 0, 0.5, 0. Gd1: −0.1357(3), 0.25, 0.6319(3). **Gd2:** −0.4610(3), 0.25, 0.0824(3). **B1:** −0.433(5), 0.25, 0.474(4). **H11:** −0.37(2), 0.25, 0.402(14). **H12:** −0.34(2), 0.25, 0.563(11). **H13:** −0.512(6), 0.172(3), 0.466(12). **B2:** 0.155(6), 0.25, 0.540(4). **H21:** 0.293(8), 0.25, 0.573(16). **H22:** 0.10(2), 0.25, 0.618(11). **H23:** 0.114(13), 0.172(3), 0.485(5). **B3:** −0.050(6), 0.25, 0.850(4). **H31:** 0.064(16), 0.25, 0.926(14). **H32:** −0.161(17), 0.25, 0.887(18). **H33:** −0.052(16), 0.172(3), 0.794(5). **B4:** 0.493(6), 0.25, 0.862(5). **H41:** 0.356(8), 0.25, 0.827(17). **H42:** 0.55(2), 0.25, 0.785(12). **H43:** 0.533(13), 0.172(3), 0.917(6). **B5:** −0.206(4), −0.515(2), 0.592(3). **H51:** −0.20(3), −0.477(12), 0.505(7). **H52:** −0.11(3), −0.48(2), 0.667(8). **H53:** −0.19(6), −0.609(7), 0.591(9). **H54:** −0.331(13), −0.50(4), 0.605(13). **B6:** −0.653(4), 0.100(2), 0.130(3). **H61:** −0.788(8), 0.116(16), 0.119(14). **H62:** −0.621(19), 0.119(9), 0.044(8). **H63:** −0.62(2), 0.009(7), 0.157(14). **H64:** −0.580(19), 0.158(14), 0.202(11). **B7:** −0.212(4), 0.108(2), 0.179(3). **H71:**−0.17(2), 0.017(6), 0.185(13). **H72:** −0.204(13), 0.145(11), 0.092(7). **H73:** −0.132(19), 0.158(11), 0.254(8). **H74:** −0.343(8), 0.111(15), 0.186(12)	[20] experimental 298 K
KGd(BH_4_)_4_ 6.31	Space group: monoclinic *P2_1_/c* (14) a = 7.1051(6), b = 7.7365(6), c = 8.1049(6), α = γ = 90, β = 102.192(4)	**K1:** 0.5, 0, 0.5. **Gd1:** 0, 0.5, 0.5. **B1:** −0.107(8), 0.771(7), 0.328(7). **H11:** −0.10(6), 0.71(3), 0.46(2). **H12:** 0.01(6), 0.70(6), 0.27(4). **H13:** −0.07(11), 0.92(2), 0.34(5). **H14:** −0.26(3), 0.75(10), 0.24(4). **B2:** 0.653(8), 0.146(5), 0.900(7). **H21:** 0.56(3), 0.27(2), 0.92(3). **H22:** 0.55(3), 0.03(2), 0.86(3). **H23:** 0.76(3), 0.12(3), 1.027(18). **H24:** 0.74(3), 0.17(3), 0.80(2)	[20] experimental 450 K
Cs_3_Gd(BH_4_)_6_ 3.75	Space group: cubic *F23* (196) a = b = c = 11.3000(1),α = β = γ = 90	**Cs1:** 0, 0, 0. **Cs2:** 0, 0.5, 0. **Cs3:** 0.75, 0.75, 0.75. **Gd:** 0.2503, 0.7495, 0.7483. **B:** 0.7498, 0.7499, 0.4965. **H1:** 0.8059, 0.8100, 0.5550. **H2:** 0.6900, 0.8062, 0.4380	[20] experimental 298 K

**Table 16:** Crystal structures of Er-borohydrides.

Material and Hydrogen Content [wt.%]	Structural Parameters [Å, °]	Atomic Positions	Comments
NaEr(BH_4_)_4_ 6.46	Space group: orthorhombic *Cmcm* (63) a = 8.5379(2), b = 12.1570(4), c = 9.1652(3) α= β= γ = 90	**Na11:** 0, 0, 0.5. **Er12:** 0.5, 0.1430, 0.75. **B13:** 0.5, 0.2630, 0.9630. **H14:** 0.5, 0.3200, 0.0640. **H15:** 0.5, 0.1720, 1.0020. **H16:** 0.6113, 0.2800, 0.8930. **B17:** 0.7390, 0.0410, 0.75. **H18:** 0.8660, 0.0060, 0.75. **H19:** 0.7440, 0.1360, 0.75. **H110:** 0.6730, 0.0110, 0.85368	[204]

**Table 17:** Crystal structures of Yb-borohydrides.

Material and Hydrogen Content [wt.%]	Structural Parameters [Å, °]	Atomic Positions	Comments
α- Yb(BH_4_)_2_ 5.56	Space group: cubic *Pa-3* (205) a = b= c = 10.70715(15) α= β= γ = 90	**Yb:** 0.71615(5), 0. 71615(5), 0. 71615(5). **B:** −0.0391(12), 0.6968(11), 0.7536(18). **H1:** −0.104(6), 0.640(6), 0.684(6). **H2:** 0.064(3), 0.652(5), 0.753(8). **H3:** −0.079(7), 0.693(7), 0.857(3). **H4:** −0.031(6), 0.803(2), 0.720(7)	[198]
β-Yb(BH_4_)_2_ 5.56	Space group: cubic *Pm-3m* (221) a = b = c = 5.44223(3) α = β = γ = 90	**Yb:** 0, 0, 0. **B:** ½, 0, 0. **H:** 0.3745(4), 0, 0.1826(3)	[198]

## Data Availability

The data presented in this review was collected from the cited articles.

## Supplementary Materials

The following are available online at https://www.mdpi.com/article/10.3390/ma14102561/s1, Figure S1: Editable version of the Figure 1. Figure S2: Editable version of the Figure 2.
