# Supplementary material for: Metal Borohydrides beyond Groups I and II: A Review
Source: Materials (Basel). 2021 May 14;14(10):2561. doi: 10.3390/ma14102561 (PMC8156325; doi:10.3390/ma14102561)
Supplement: Supplementary file 1 [file materials-14-02561-s001.zip › materials-1192398-supplementary.pptx]

## Slide 1
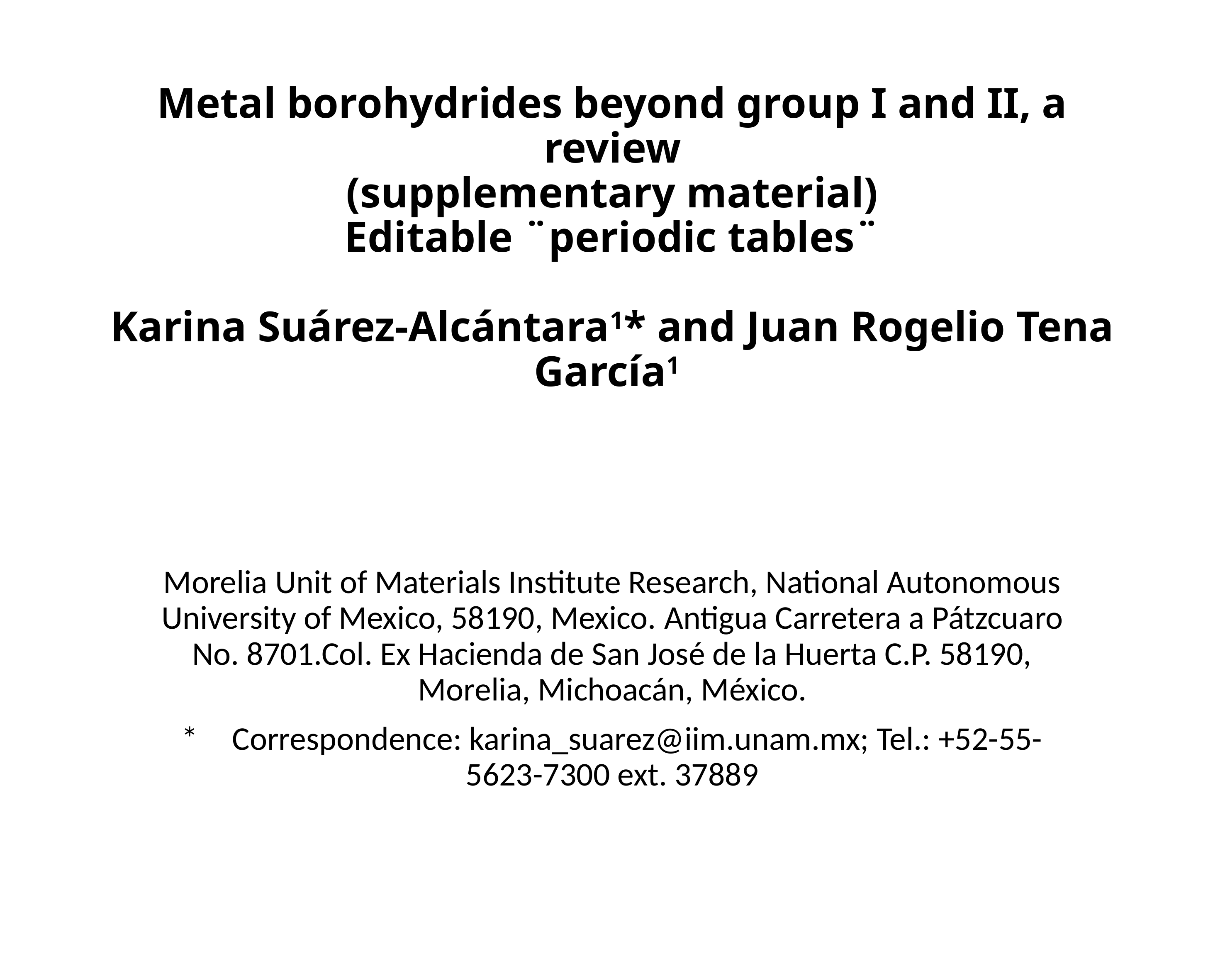

# Metal borohydrides beyond group I and II, a review (supplementary material) Editable ¨periodic tables¨ Karina Suárez-Alcántara1* and Juan Rogelio Tena García1
Morelia Unit of Materials Institute Research, National Autonomous University of Mexico, 58190, Mexico. Antigua Carretera a Pátzcuaro No. 8701.Col. Ex Hacienda de San José de la Huerta C.P. 58190, Morelia, Michoacán, México.
*	Correspondence: karina_suarez@iim.unam.mx; Tel.: +52-55-5623-7300 ext. 37889

## Slide 2
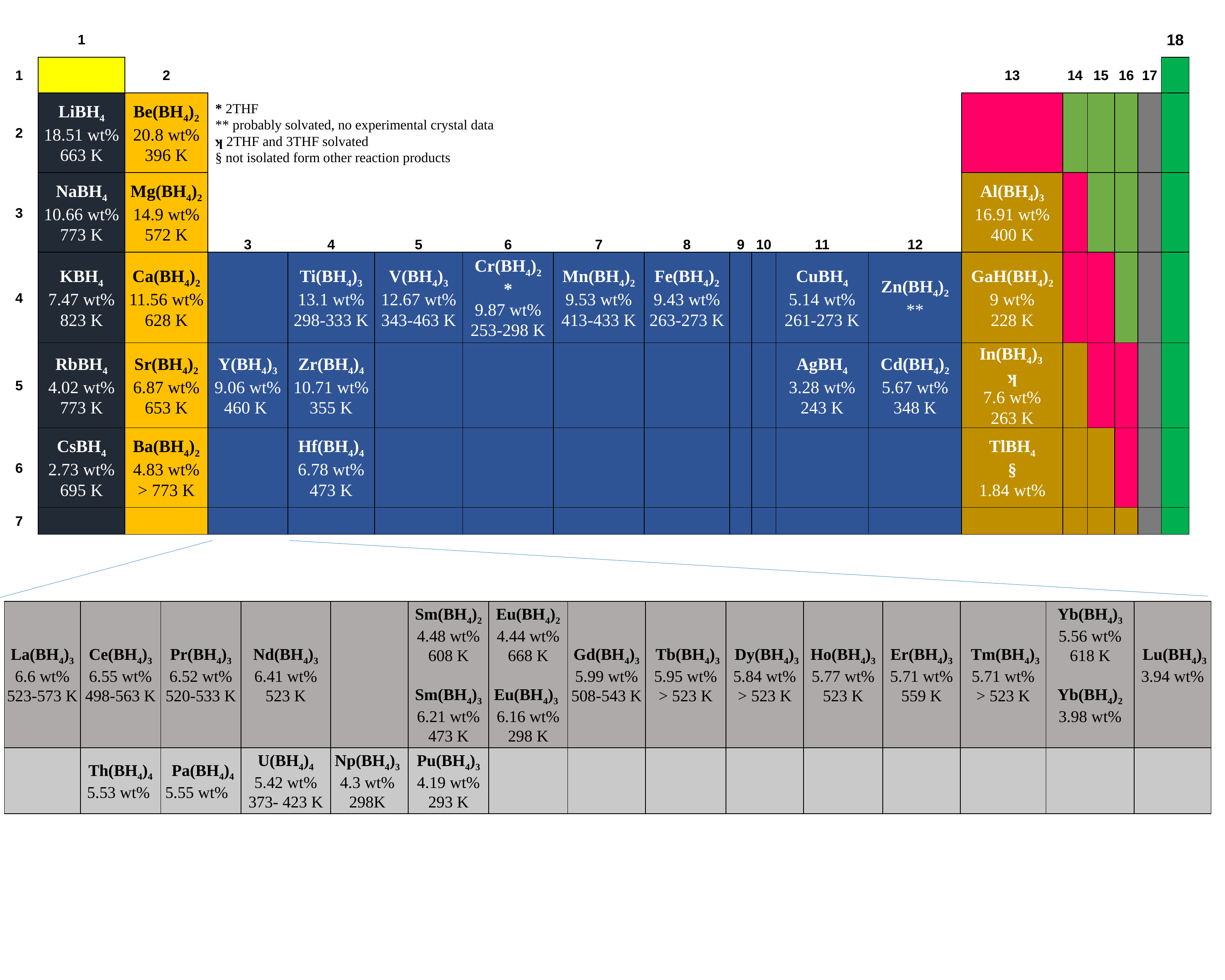

| | 1 | | | | | | | | | | | | | | | | | | | | 18 |
| --- | --- | --- | --- | --- | --- | --- | --- | --- | --- | --- | --- | --- | --- | --- | --- | --- | --- | --- | --- | --- | --- |
| 1 | | 2 | | | | | | | | | | | | | | 13 | 14 | 15 | 16 | 17 | |
| 2 | LiBH4 18.51 wt% 663 K | Be(BH4)2 20.8 wt% 396 K | \* 2THF  \*\* probably solvated, no experimental crystal data ʞ 2THF and 3THF solvated § not isolated form other reaction products | | | | | | | | | | | | | | | | | | |
| 3 | NaBH4 10.66 wt% 773 K | Mg(BH4)2 14.9 wt% 572 K | 3 | 4 | | 5 | | 6 | | 7 | 8 | 9 | 10 | 11 | 12 | Al(BH4)3 16.91 wt% 400 K | | | | | |
| 4 | KBH4 7.47 wt% 823 K | Ca(BH4)2 11.56 wt% 628 K | | Ti(BH4)3 13.1 wt% 298-333 K | | V(BH4)3 12.67 wt% 343-463 K | | Cr(BH4)2 \* 9.87 wt% 253-298 K | | Mn(BH4)2 9.53 wt% 413-433 K | Fe(BH4)2 9.43 wt% 263-273 K | | | CuBH4 5.14 wt% 261-273 K | Zn(BH4)2 \*\* | GaH(BH4)2 9 wt% 228 K | | | | | |
| 5 | RbBH4 4.02 wt% 773 K | Sr(BH4)2 6.87 wt% 653 K | Y(BH4)3 9.06 wt% 460 K | Zr(BH4)4 10.71 wt% 355 K | | | | | | | | | | AgBH4 3.28 wt% 243 K | Cd(BH4)2 5.67 wt% 348 K | In(BH4)3 ʞ 7.6 wt% 263 K | | | | | |
| 6 | CsBH4 2.73 wt% 695 K | Ba(BH4)2 4.83 wt% > 773 K | | Hf(BH4)4 6.78 wt% 473 K | | | | | | | | | | | | TlBH4 § 1.84 wt% | | | | | |
| 7 | | | | | | | | | | | | | | | | | | | | | |
| La(BH4)3 6.6 wt% 523-573 K | Ce(BH4)3 6.55 wt% 498-563 K | Pr(BH4)3 6.52 wt% 520-533 K | Nd(BH4)3 6.41 wt% 523 K | | Sm(BH4)2 4.48 wt% 608 K Sm(BH4)3 6.21 wt% 473 K | Eu(BH4)2 4.44 wt% 668 K Eu(BH4)3 6.16 wt% 298 K | Gd(BH4)3 5.99 wt% 508-543 K | Tb(BH4)3 5.95 wt% > 523 K | Dy(BH4)3 5.84 wt% > 523 K | Ho(BH4)3 5.77 wt% 523 K | Er(BH4)3 5.71 wt% 559 K | Tm(BH4)3 5.71 wt% > 523 K | Yb(BH4)3 5.56 wt% 618 K Yb(BH4)2 3.98 wt% | Lu(BH4)3 3.94 wt% |
| --- | --- | --- | --- | --- | --- | --- | --- | --- | --- | --- | --- | --- | --- | --- |
| | Th(BH4)4 5.53 wt% | Pa(BH4)4 5.55 wt% | U(BH4)4 5.42 wt% 373- 423 K | Np(BH4)3 4.3 wt% 298K | Pu(BH4)3 4.19 wt% 293 K | | | | | | | | | |

## Slide 3
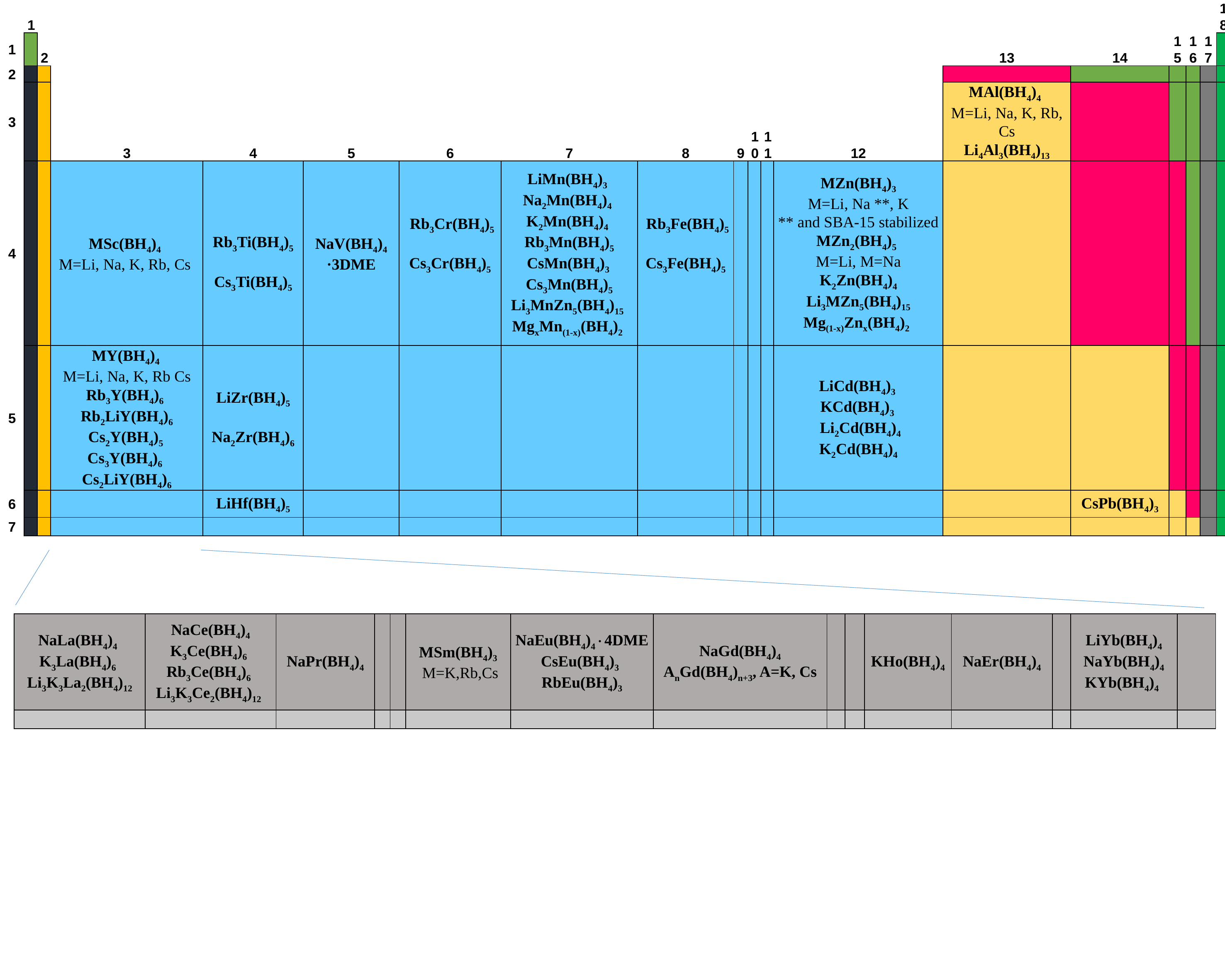

| | 1 | | | | | | | | | | | | | | | | | 18 |
| --- | --- | --- | --- | --- | --- | --- | --- | --- | --- | --- | --- | --- | --- | --- | --- | --- | --- | --- |
| 1 | | 2 | | | | | | | | | | | 13 | 14 | 15 | 16 | 17 | |
| 2 | | | | | | | | | | | | | | | | | | |
| 3 | | | 3 | 4 | 5 | 6 | 7 | 8 | 9 | 10 | 11 | 12 | MAl(BH4)4 M=Li, Na, K, Rb, Cs Li4Al3(BH4)13 | | | | | |
| 4 | | | MSc(BH4)4 M=Li, Na, K, Rb, Cs | Rb3Ti(BH4)5 Cs3Ti(BH4)5 | NaV(BH4)4 ·3DME | Rb3Cr(BH4)5 Cs3Cr(BH4)5 | LiMn(BH4)3 Na2Mn(BH4)4 K2Mn(BH4)4 Rb3Mn(BH4)5 CsMn(BH4)3 Cs3Mn(BH4)5 Li3MnZn5(BH4)15 MgxMn(1-x)(BH4)2 | Rb3Fe(BH4)5 Cs3Fe(BH4)5 | | | | MZn(BH4)3 M=Li, Na \*\*, K \*\* and SBA-15 stabilized MZn2(BH4)5 M=Li, M=Na K2Zn(BH4)4 Li3MZn5(BH4)15 Mg(1-x)Znx(BH4)2 | | | | | | |
| 5 | | | MY(BH4)4 M=Li, Na, K, Rb Cs Rb3Y(BH4)6 Rb2LiY(BH4)6 Cs2Y(BH4)5 Cs3Y(BH4)6 Cs2LiY(BH4)6 | LiZr(BH4)5 Na2Zr(BH4)6 | | | | | | | | LiCd(BH4)3 KCd(BH4)3  Li2Cd(BH4)4 K2Cd(BH4)4 | | | | | | |
| 6 | | | | LiHf(BH4)5 | | | | | | | | | | CsPb(BH4)3 | | | | |
| 7 | | | | | | | | | | | | | | | | | | |
| NaLa(BH4)4 K3La(BH4)6 Li3K3La2(BH4)12 | NaCe(BH4)4 K3Ce(BH4)6 Rb3Ce(BH4)6 Li3K3Ce2(BH4)12 | NaPr(BH4)4 | | | MSm(BH4)3 M=K,Rb,Cs | NaEu(BH4)44DME CsEu(BH4)3  RbEu(BH4)3 | NaGd(BH4)4 AnGd(BH4)n+3, A=K, Cs | | | KHo(BH4)4 | NaEr(BH4)4 | | LiYb(BH4)4 NaYb(BH4)4 KYb(BH4)4 | |
| --- | --- | --- | --- | --- | --- | --- | --- | --- | --- | --- | --- | --- | --- | --- |
| | | | | | | | | | | | | | | |

## Slide 4
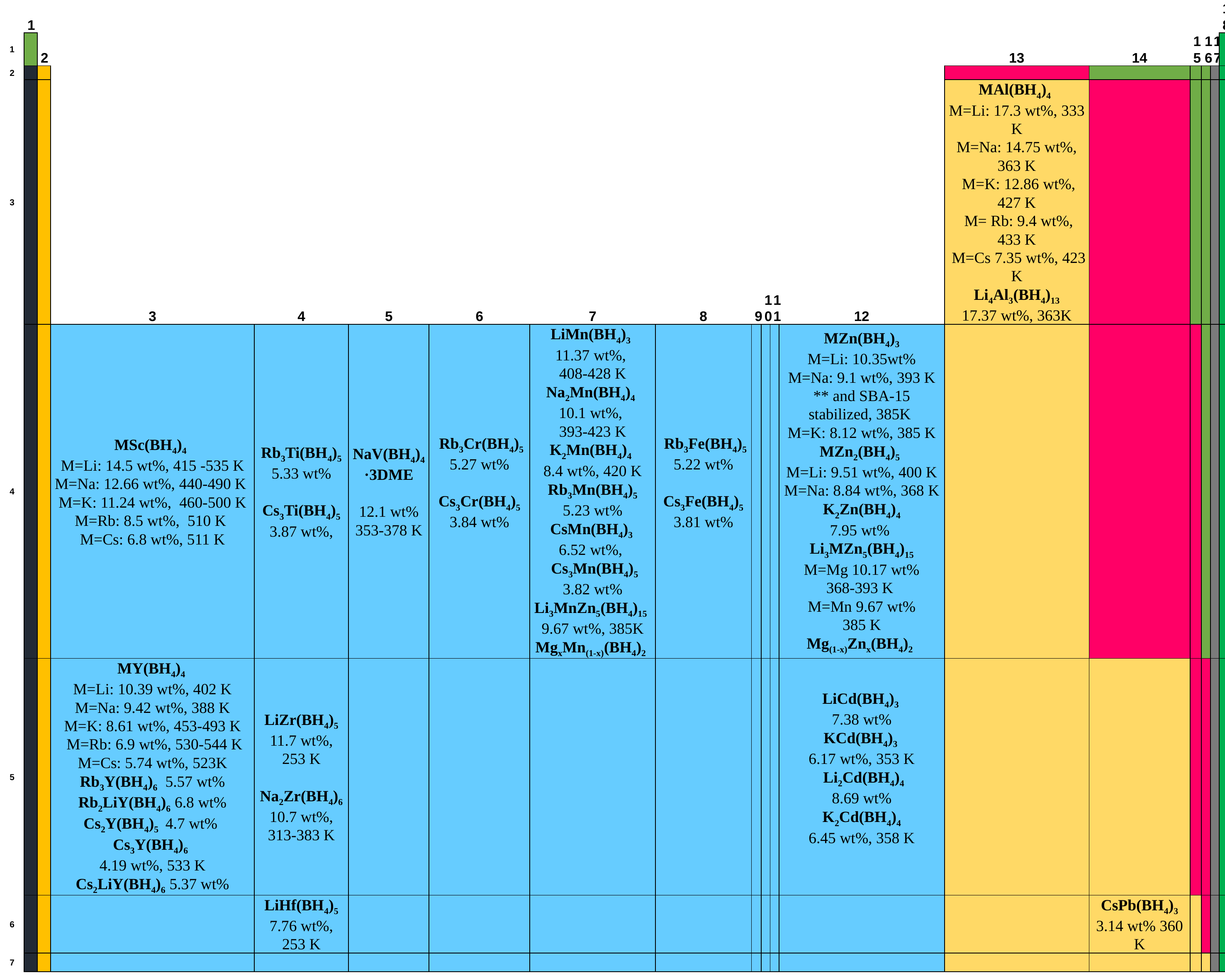

| | 1 | | | | | | | | | | | | | | | | | 18 |
| --- | --- | --- | --- | --- | --- | --- | --- | --- | --- | --- | --- | --- | --- | --- | --- | --- | --- | --- |
| 1 | | 2 | | | | | | | | | | | 13 | 14 | 15 | 16 | 17 | |
| 2 | | | | | | | | | | | | | | | | | | |
| 3 | | | 3 | 4 | 5 | 6 | 7 | 8 | 9 | 10 | 11 | 12 | MAl(BH4)4 M=Li: 17.3 wt%, 333 K M=Na: 14.75 wt%, 363 K  M=K: 12.86 wt%, 427 K  M= Rb: 9.4 wt%, 433 K  M=Cs 7.35 wt%, 423 K Li4Al3(BH4)13 17.37 wt%, 363K | | | | | |
| 4 | | | MSc(BH4)4 M=Li: 14.5 wt%, 415 -535 K M=Na: 12.66 wt%, 440-490 K M=K: 11.24 wt%, 460-500 K M=Rb: 8.5 wt%, 510 K M=Cs: 6.8 wt%, 511 K | Rb3Ti(BH4)5 5.33 wt%   Cs3Ti(BH4)5 3.87 wt%, | NaV(BH4)4 ·3DME 12.1 wt% 353-378 K | Rb3Cr(BH4)5 5.27 wt%   Cs3Cr(BH4)5 3.84 wt% | LiMn(BH4)3 11.37 wt%, 408-428 K Na2Mn(BH4)4 10.1 wt%, 393-423 K K2Mn(BH4)4 8.4 wt%, 420 K Rb3Mn(BH4)5 5.23 wt% CsMn(BH4)3 6.52 wt%,  Cs3Mn(BH4)5 3.82 wt% Li3MnZn5(BH4)15 9.67 wt%, 385K MgxMn(1-x)(BH4)2 | Rb3Fe(BH4)5 5.22 wt%   Cs3Fe(BH4)5 3.81 wt% | | | | MZn(BH4)3 M=Li: 10.35wt% M=Na: 9.1 wt%, 393 K \*\* and SBA-15 stabilized, 385K M=K: 8.12 wt%, 385 K MZn2(BH4)5 M=Li: 9.51 wt%, 400 K M=Na: 8.84 wt%, 368 K K2Zn(BH4)4 7.95 wt%  Li3MZn5(BH4)15 M=Mg 10.17 wt% 368-393 K M=Mn 9.67 wt% 385 K Mg(1-x)Znx(BH4)2 | | | | | | |
| 5 | | | MY(BH4)4 M=Li: 10.39 wt%, 402 K M=Na: 9.42 wt%, 388 K M=K: 8.61 wt%, 453-493 K  M=Rb: 6.9 wt%, 530-544 K M=Cs: 5.74 wt%, 523K Rb3Y(BH4)6 5.57 wt% Rb2LiY(BH4)6 6.8 wt% Cs2Y(BH4)5 4.7 wt% Cs3Y(BH4)6 4.19 wt%, 533 K Cs2LiY(BH4)6 5.37 wt% | LiZr(BH4)5 11.7 wt%, 253 K   Na2Zr(BH4)6 10.7 wt%, 313-383 K | | | | | | | | LiCd(BH4)3 7.38 wt% KCd(BH4)3 6.17 wt%, 353 K  Li2Cd(BH4)4 8.69 wt% K2Cd(BH4)4 6.45 wt%, 358 K | | | | | | |
| 6 | | | | LiHf(BH4)5 7.76 wt%, 253 K | | | | | | | | | | CsPb(BH4)3 3.14 wt% 360 K | | | | |
| 7 | | | | | | | | | | | | | | | | | | |

## Slide 5
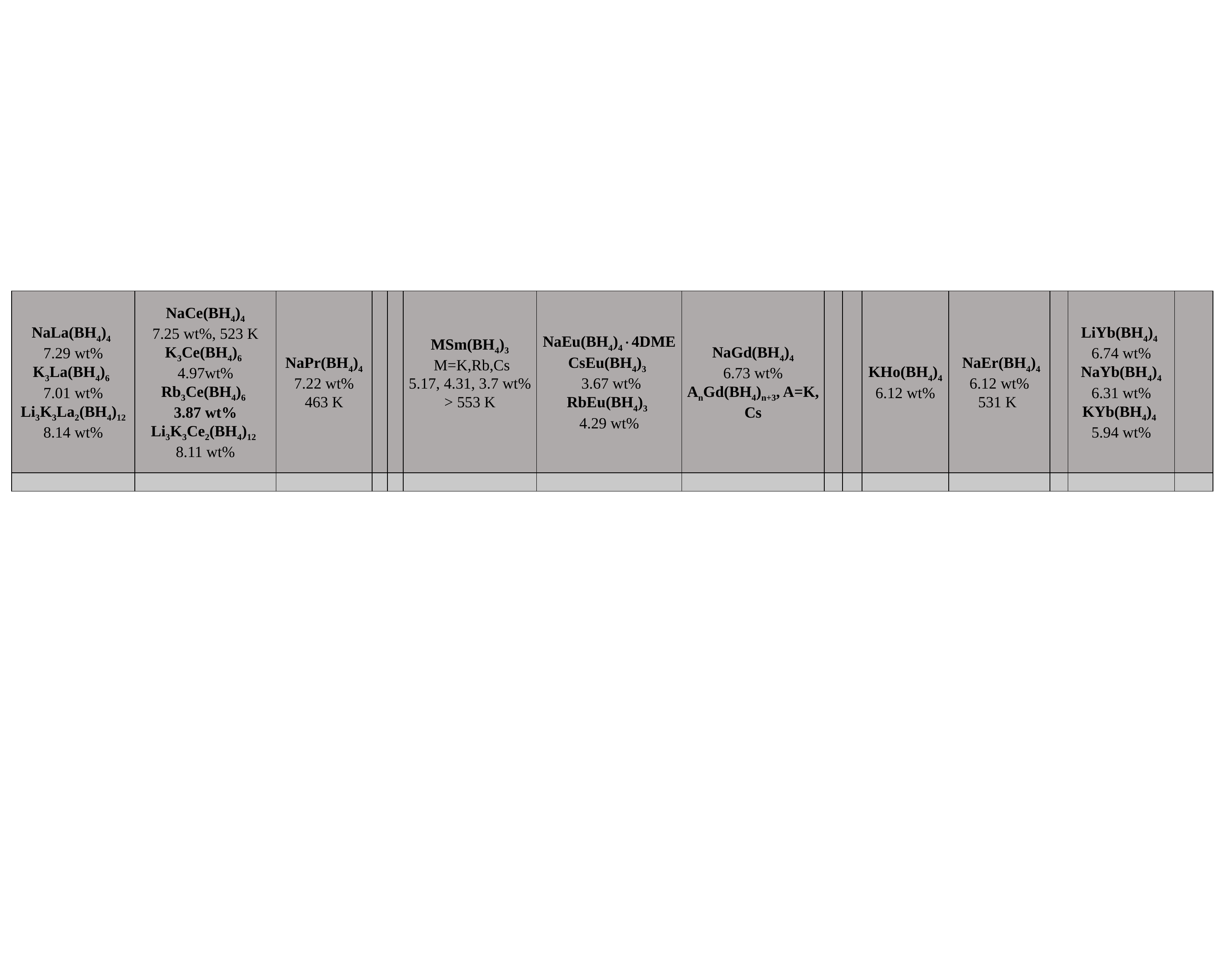

| NaLa(BH4)4 7.29 wt% K3La(BH4)6 7.01 wt% Li3K3La2(BH4)12 8.14 wt% | NaCe(BH4)4 7.25 wt%, 523 K K3Ce(BH4)6 4.97wt% Rb3Ce(BH4)6 3.87 wt% Li3K3Ce2(BH4)12 8.11 wt% | NaPr(BH4)4 7.22 wt% 463 K | | | MSm(BH4)3 M=K,Rb,Cs 5.17, 4.31, 3.7 wt% > 553 K | NaEu(BH4)44DME CsEu(BH4)3  3.67 wt% RbEu(BH4)3 4.29 wt% | NaGd(BH4)4 6.73 wt% AnGd(BH4)n+3, A=K, Cs | | | KHo(BH4)4 6.12 wt% | NaEr(BH4)4 6.12 wt% 531 K | | LiYb(BH4)4 6.74 wt% NaYb(BH4)4 6.31 wt% KYb(BH4)4 5.94 wt% | |
| --- | --- | --- | --- | --- | --- | --- | --- | --- | --- | --- | --- | --- | --- | --- |
| | | | | | | | | | | | | | | |
